# hzAnalyzer: detection, quantification, and visualization of contiguous homozygosity in high-density genotyping datasets

**DOI:** 10.1186/gb-2011-12-3-r21

**Published:** 2011-03-11

**Authors:** Todd A Johnson, Yoshihito Niimura, Hiroshi Tanaka, Yusuke Nakamura, Tatsuhiko Tsunoda

**Affiliations:** 1 Laboratory for Medical Informatics, Center for Genomic Medicine, RIKEN Yokohama Institute, Suehiro-cho, Tsurumi-ku, Yokohama, Kanagawa-ken, 230-0045, Japan; 2 Department of Bioinformatics, Medical Research Institute, Tokyo Medical and Dental University, Yushima, Bunkyo-ku, Tokyo, 113-8510, Japan; 3 Department of Bioinformatics, School of Biomedical Science, Tokyo Medical and Dental University, Yushima, Bunkyo-ku, Tokyo, 113-8510, Japan; 4 Human Genome Center, Institute of Medical Science, University of Tokyo, Shirokanedai, Minato-ku, Tokyo, 108-8639, Japan

## Abstract

The analysis of contiguous homozygosity (runs of homozygous loci) in human genotyping datasets is critical in the search for causal disease variants in monogenic disorders, studies of population history and the identification of targets of natural selection. Here, we report methods for extracting homozygous segments from high-density genotyping datasets, quantifying their local genomic structure, identifying outstanding regions within the genome and visualizing results for comparative analysis between population samples.

## Background

Homozygosity represents a simple but important concept for exploring human population history, the structure of human genetic variation, and their intersection with human disease. At its most basic level, homozygosity means that, for a particular locus, the two copies that are inherited from an individual's parents both have the same allelic value and are identical-by-state. However, if the two homologues originate from the same ancestor in their genealogic histories, then the two copies can be described as being identical-by-descent and the locus referred to as autozygous [1]. While autozygosity stems from recent relatedness between an individual's parents, shared ancestry from the much more distant past can nevertheless result in portions of any two homologous chromosomes being homozygous by descent, reflecting background relatedness within a population [2]. Researchers need to integrate information across multiple contiguous homozygous SNPs in an individual's genome to detect such homozygous segments, which, by their very nature, represent known haplotypes within otherwise phase-unknown datasets. As such, they potentially represent a higher-level abstraction of information than that which can be obtained from analysis of just single SNPs. Since this has potential for identifying shared haplotypes that harbor disease variants that escape current single-marker statistical tests, the field would benefit from additional software tools and methodologies for strengthening our understanding of the distribution and variation of homozygous segments/contiguous homozygosity within human population samples.

Early attempts to understand the contribution of contiguous homozygosity to the structure of genetic variation in modern human populations identified regions of increased homozygous genotypes in individuals that likely represented autozygosity [3]. However, due to technological limitations at the time, their micro-satellite-based scan limited resolution of segments to those of an appreciably large size: generally, much greater than one centimorgan (1 cM). Since then, the International HapMap Project, which was initiated in 2002, provided researchers with a high-density SNP dataset [4,5] consisting of genome-wide genotypes from 270 individuals in four world-wide human populations (YRI, Yoruba in Ibadan, Nigeria; CEU, Utah residents with ancestry from northern and western Europe; CHB, Han Chinese in Beijing, China; JPT, Japanese in Tokyo, Japan).

Using the HapMap Phase I dataset, Gibson *et al. *[6] searched for tracts of contiguous homozygous loci greater than 1 Mb in length and found 1,393 such tracts among the 209 unrelated HapMap individuals. Their analysis also showed that regions of high linkage disequilibrium (LD) harbored significantly more homozygous tracts and that local tract coverage was often correlated between the four populations. Our own analysis of the HapMap Phase 2 dataset further quantified the relative total levels of contiguous homozygosity between the four HapMap population samples and showed that average total length of homozygosity was highest and almost equal between JPT and CHB, lowest in YRI, and of an intermediate level in CEU (mean total megabase length of homozygous segments ≥106 kb: JPT = 520, CHB = 510, CEU = 410, YRI = 160) [5]. A number of groups have also examined extended homozygosity (that is, regions of contiguous homozygosity that appear longer than expected) using non-HapMap population samples with commercially available whole-genome genotyping platforms. Among these studies, a non-trivial percentage of several presumably outbred population samples were observed to possess long homozygous segments [7-12]. In addition, high frequency contiguous homozygosity was noted to reflect the underlying frequency of inferred haplotypes [9,13], and the total extent of contiguous homozygosity (segments greater than 1 Mb in length) was recently used to assist in the analysis of the population structure of Finnish subgroups [14]. Other recent reports have described methods for finding recessive disease variants by detecting regions of excess homozygosity in unrelated case/control samples in diseases such as schizophrenia, Alzheimer's disease, and Parkinson's disease [15-17]. As for available homozygous segment detection methods and computer programs, several studies have utilized their own in-house programs [7,9,10,13,15] while the genetic analysis application PLINK [18] has been used in several other reports [11,12,14] for detecting runs of homozygosity (ROH).

Here, we introduce hzAnalyzer, a new R package [19] that we have developed for detection, quantification, and visualization of homozygous segments/ROH in high-density SNP datasets. hzAnalyzer provides a comprehensive set of functions for analysis of contiguous homozygosity, including a robust algorithm for homozygous segment/ROH detection, a novel measure (termed ext_AUC _(extent-area under the curve)) for quantifying the local genomic extent of contiguous homozygosity, routines for peak detection and processing, and methods for comparing population differentiation (Fst/θ). Using the HapMap Phase 2 dataset, we compare hzAnalyzer with PLINK's ROH output and describe the advantages of using hzAnalyzer for performing homozyous segment detection. We then extend our previous analysis [5] by examining the relative contribution of different sized homozygous segments to chromosomal coverage, followed by mapping ext_AUC _and its associated statistics to the human genome. We examine the consistency of these analyses with the structure and frequency of phased haplotype data, their relationship with recombination rate estimates, and show how one can use ext_AUC _peak definition in combination with Fst/θ to extract genomic regions harboring long multi-locus haplotypes with large inter-population frequency differences. We additionally describe detection of candidate regions of fixation and highlight genes in these regions that appear to have been important during human evolutionary history. To show how these methods can be used for practical real-world applications, we introduce a method for searching for regions of excess homozygosity that could be used to compare case-control samples for genome-wide association studies.

## Results

In this report, we describe the methodology behind hzAnalyzer by examining variation in the local extent of contiguous homozygosity across the human genome using approximately 3 million SNPs from the 269 fully genotyped samples of the HapMap Phase 2 dataset [5,20]. For hzAnalyzer methods and implementation details, we refer readers to the Materials and methods section of this report as well as to the hzAnalyzer homepage [21], from which the R package, tutorials, and example datasets can be downloaded.

### Homozygous segment detection, validation, and annotation

After processing the HapMap release 24 SNPs for certain quality control parameters (see Materials and methods), we built a dataset of homozygous segments' coordinates and characteristics using hzAnalyzer's Java-based detection function to extract runs of contiguous homozygous loci (see Materials and methods). To remove the many short segments that were due simply to background random variation, we filtered this dataset prior to downstream analyses using a new cross-population version of the previously described homozygosity probability score (HPS_ex_; < = 0.01; see Materials and methods) [5].

To validate our detection algorithm, we compared ROH output between hzAnalyzer and PLINK [18], which is the only free, open source genetics analysis program that we found to contain an ROH detection routine. Table 1 shows that the majority of segments in each dataset intersected a single segment in the other dataset. However, 36.7% of PLINK ROHs overlapped two or more hzAnalyzer segments, whereas the reverse comparison showed only 101 (1.7%) multi-hit segments. Algorithmic differences for handling heterozygote 'error' and large inter-SNP gaps apparently accounted for the larger number of multi-hit PLINK runs, with PLINK joining shorter ROH (approximately <100 SNPs) broken by single heterozygotes. During our preliminary analyses, we had concluded that 1% was an appropriate maximum for ROH heterozygosity, but PLINK's default settings resulted in runs with up to 3% heterozygous loci. Analysis of multi-hit hzAnalyzer segments indicated that PLINK had split a number of runs with over several thousand loci into smaller ROHs. A likely cause of this discrepancy were random groups of no-call genotypes that exceeded PLINK's default settings (--homozyg-window-missing = 5). Furthermore, the hzAnalyzer segments (n = 440) that had no overlapping segments in the PLINK (>1 Mb) set appeared to possess levels of either no-calls or heterozygotes that exceeded PLINK's window cutoff values. All PLINK segments with no overlap with hzAnalyzer output were segments with less than 250 SNPs that had heterozygosity greater than hzAnalyzer's 1% maximum cutoff.

**Table 1:** Comparison of segment overlap counts between hzAnalyzer and PLINK homozygous segment/runs-of-homozygosity detection routines

		Number of intersecting segments in other dataset					
		
Dataset	Total segments	0	1	2	3-5	6-10	>10
hzAnalyzer	5,781	440	5,240	93	7	0	1
PLINK	8,040	30	5,059	1,777	1,108	66	0

Runs of homozygosity were detected using PLINK's default settings (ROH >1 Mb), and a corresponding set of homozygous segments with >1 Mb length selected from the complete hzAnalyzer dataset. The PLINK set was intersected with segments with ≥50 SNP/segment from the complete hzAnalyzer dataset, and the reverse intersection was performed between the hzAnalyzer (>1 Mb) and PLINK (>1 Mb) sets.

**Figure 1:**
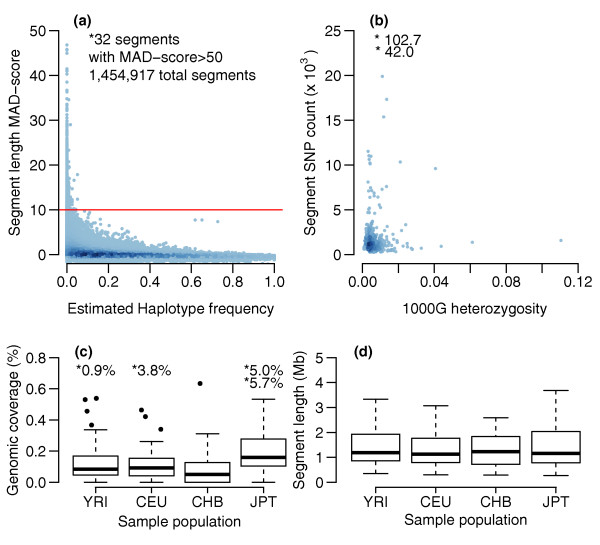
**Identification and summary of putative autozygous segments**. **(a) **High MAD score homozygous segments originate from low frequency haplotypes: for each homozygous segment, a length-based MAD score was calculated and the frequency of haplotypes matching a segment's founder haplotypes estimated within each sample population. A two-dimensional density estimate between the two variables used R's densCols function with nbin = 1,024. **(b) **Concordance between 1000G data and putative autozygous segments: putative autozygous segments' SNP counts in HapMap Phase 2 compared with heterozygosity in 1000 Genomes Project genotypes. **(c,d) **Boxplot summaries of putative autozygous segments: (c) genome-wide percent coverage by individual; (d) segment length (outliers not shown). Putative autozygous segments defined as MAD score >10 and founder haplotype frequency = 0.0000. Asterisks mark values that are above the y-axis limit.

Additional file 1, which shows greater confidence hzAnalyzer segments after applying a chromosome-specific minimum inclusive segment length threshold (MISL_chr_; see Materials and methods and Table S1 in Additional file 2), allows one to discern regions of apparent increased LD made up of co-localized segments that are common in a population (that is, of intermediate and high frequencies). In addition, some very long segments, likely representing autozygous segments, can be observed to span across multiple such regions of increased LD (for example: Chr 2, JPT 20 to 40 Mb; Chr 3, JPT 72 to 117 Mb; Chr 14, YRI 75 to 82 Mb). Since such long segments can affect some of the quantification methods described below, we developed a median-absolute deviation (MAD) score based on segment length analysis to identify and mask their effect on the dataset (see Materials and methods). Based on Figure 1a, which shows segments' MAD scores versus estimated founder haplotype frequency, we defined segments for masking as those with a MAD score >10 (904 segments; 253 samples) and defined putative autozygous segments for further analysis as the subset that also had estimated haplotype frequency equal to zero (636 segments; 231 samples). All high MAD score segments are colored green in Additional file 1 and their coordinates saved in Table S2a-d in Additional file 2. To further validate the set of putative autozygous segments, we intersected their coordinates with next-generation sequencing data from the 1000 Genomes Project (1000G; see Materials and methods) [22]. In Figure 1b, the low level of heterozygosity (0.7 ± 0.8%; mean ± standard deviation (SD), n = 413: YRI = 103, CEU = 102, CHB = 59, JPT = 149) in segments with 1000G data supports the validity of our approach for detecting putative autozygous segments, although a small number of those segments had relatively high heterozygosity levels (heterozygosity >1.48%, n = 26, 6.7%). Examination of the latter segments appeared to indicate a positive relationship between increasing 1000G heterozygosity and the proportions of large gaps, which likely reflect regions of structural variation. However, some segments with many thousands of loci, which fairly conclusively represent true autozygosity, nevertheless possessed greater than 4% heterozygosity in 1000G. Therefore, it is currently not possible to determine whether such discrepancies reflect false positive autozygous calls or rather regions of the genome that possess increased error rates in 1000G.

Sample variation in the amount of the genome and individual chromosomes covered by autozygous segments is of potential interest for both population geneticists as well as those interested in disease research. Figure 1c identifies several individuals in each population sample (YRI = 5/90, CEU = 4/90, CHB = 1/45, JPT = 2/44) who possessed markedly higher genome-wide coverage by autozygous segments. Of those samples, several extreme outliers were detected that were previously reported (YRI, NA19201; CEU, NA12874; JPT, NA18992, NA18987) [5,6]. In Additional file 3, chromosome profiles of autozygous coverage show that each of the two extreme JPT NA18987 and NA18992 samples possessed multiple chromosomes with coverage ranging from 6.0 to 43.7%, while YRI NA19201 and CEU NA12874 had only high coverage levels on single chromosomes, with 12.9% coverage on chromosome 5 and 41.3% coverage on chromosome 1, respectively. Scanning through the chromosome profiles shows that the majority of HapMap 2 samples possess one or more chromosomes containing some small proportion of autozygosity. These profiles may be evidence of a continuum of relatedness between individuals within the sample populations, with one end represented by a small group of individuals whose parents share ancestry from just several generations in the past, and the other by individuals with parents who have little or no measurable shared ancestry. Although short autozygous segments stemming from the distant past are, by their nature, random, their presence in a majority of the population could have a cumulative impact on disease when taken across large enough sample sizes.

### Extent of chromosome-specific coverage by homozygous segments

**Figure 2:**
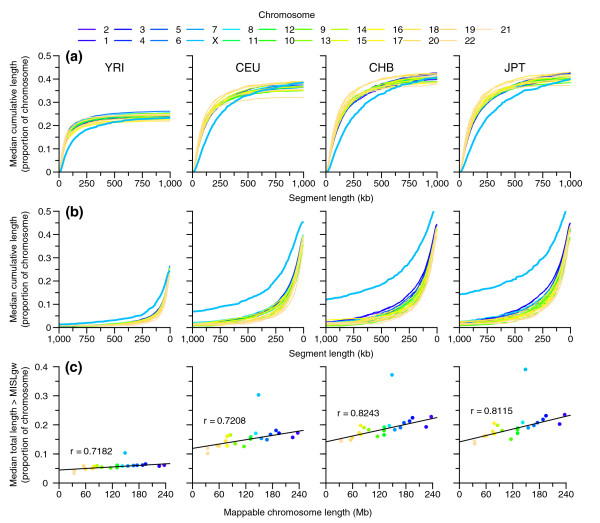
**Chromosomal coverage by homozygous segments as a function of segment size**. For each chromosome, the cumulative sum of segment length (sorted in decreasing or increasing order) was calculated for each individual, values interpolated for a set of length values between 0 and 1,000 kb, and the median value curve calculated across each sample population. **(a) **Cumulative total length (sorted by increasing segment size) as the proportion of mappable chromosomal length. **(b) **Cumulative total length (sorted by decreasing segment size) as the proportion of mappable chromosomal length. **(c) **Total segment length ≥MISL_gw _versus each chromosome's total mappable length (r shown excludes chromosome X).

In addition to coverage by autozygous segments, we were particularly interested in the distribution of homozygous segments that are common within a population. In Figure 2a,b, we examine the size distribution of homozygous segments in more detail than in our previous results [5] by calculating cumulative segment length as a proportion of each chromosome's mappable length for each individual and then computing the median values for each population evaluated at preset lengths between 0 and 1 Mb. Figure 2c shows a strong correlation (*r *between 0.7182 and 0.8243) within autosomes between mappable chromosome length and proportion coverage by long segments, defined using a genome-wide MISL (MISL_gw_; ≥131,431 bp; see Materials and methods), while, in contrast, all three Figure 2 panels show that longer segments make up a dramatically greater proportion of chromosome X compared to autosomes. Comparison of chromosome X with the closest sized autosomes (chromosome 7 and 8) using a chromosome 7, 8, and X specific MISL (MISL_chr7,8,X_; ≥315,796 bp) showed it to possess approximately two to three times greater contiguous homozygosity.

### Quantifying the local extent of contiguous homozygosity

**Figure 3:**
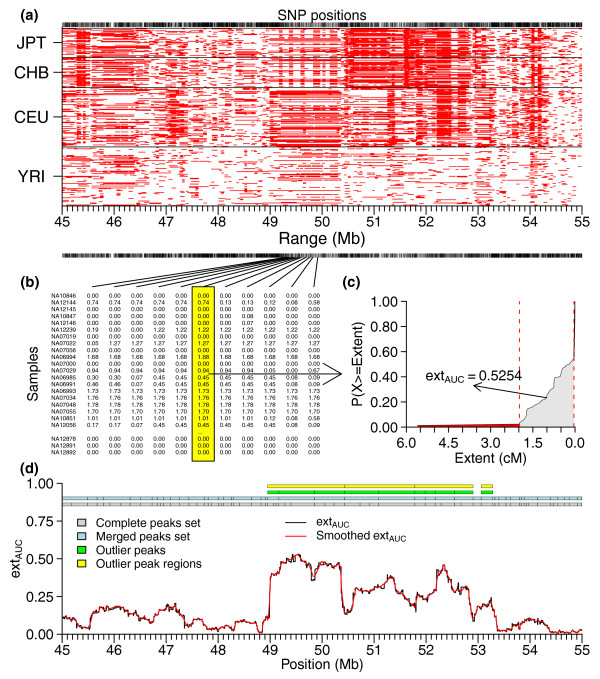
**Schematic workflow for summarizing and quantifying contiguous homozygosity**. Example 10-Mb region on chromosome 1 illustrating how observed patterns of homozygous segments are processed to create intersecting segment length matrix (ISLM_cm_), calculate ext_AUC_, and define peaks and outlier peak regions. **(a) **Coordinates of homozygous segments with length ≥49 kb (regional MISL). **(b) **Intersecting segment lengths for each locus are combined into ISLM_cm_. **(c) **An intersecting segment length vector (ISLV) is extracted from the ISLM, the sign of the values reversed, and ext_AUC _is calculated by integrating the area-under-the-curve of the empirical cumulative distribution function (ECDF) using those values. Dashed red lines mark interval of integration after masking. **(d) **ext_AUC _peak detection and processing: peaks are detected from a smooth spline function applied to ext_AUC _values, peaks with extreme peak heights selected (outlier peaks), and neighboring outlier peaks that are not well separated are merged into peak regions.

Figure 3 diagrams the hzAnalyzer workflow for quantifying local variation in the structure of contiguous homozygosity within each sample population. For each population's segments, we converted their length into centimorgans and intersected their coordinates with locus positions (Figure 3a), creating in Figure 3b what we term an intersecting segment length matrix (ISLM_cm_; see Materials and methods); each matrix column is abbreviated as ISLV for 'intersecting segment length vector'. We masked ISLV cell values that were derived from segments with a MAD score >10 (see Materials and methods), reversed the sign of ISLV values, and then calculated the empirical cumulative distribution function (ECDF) of each ISLV. We then computed the area-under-the-curve of the ECDF to derive our contiguous homozygous extent measure, which we termed ext_AUC _(Figure 3c; see Materials and methods). Pairwise comparisons of genome-wide ext_AUC _values between the four populations showed strong correlation (Pearson's correlation coefficient; two-sided test) between JPT and CHB (*r *= 0.92), moderate correlation between the two East Asian samples and CEU (*r *= 0.73 and 0.74 with CHB and JPT, respectively), and low-moderate correlation between YRI and the other three population samples (*r *= 0.64, 0.53, and 0.56 for CEU, CHB, and JPT, respectively). In addition to ext_AUC_, we calculated a related matrix, which we term the percentile-extent matrix (PE_mat_; lengths in either base pairs or converted to centimorgans), containing the percentile values for each ISLV. Additional file 4 displays a genome-wide map of the local variation of homozygous extent using the 75th percentile of PE_mat_, which we chose as representative of common variation in these population samples.

#### ext_AUC _peak detection for delineation of local haplotype structure

Earlier reports showed that homozygous segments with intermediate or high-frequencies correlate with LD statistics and co-locate with haplotype blocks [6,9]. Based on those past results, we considered that the peak/valley patterns that we observed in plotting ext_AUC _values could be used to delineate regions of the genome with locally similar structure for contiguous homozygosity; analogous to haplotype block [23] definition but using the information contained within overlapping, co-localized homozygous segments rather than statistical pairwise comparisons between loci.

**Figure 4:**
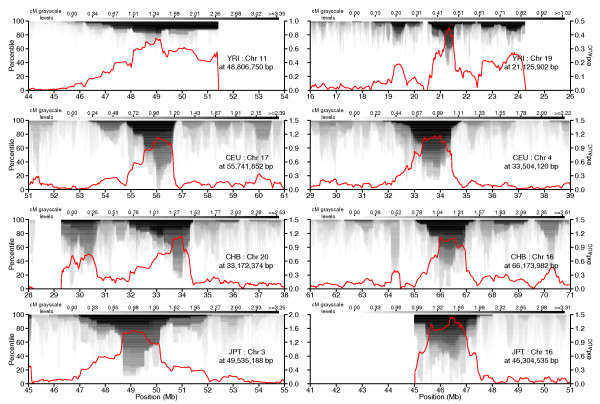
**Measures of contiguous homozygosity surrounding outlier peak regions**. Two of the top four peak regions were chosen from Table 3 for each population and centimorgan values from the percentile extent matrix (PE_mat_) for the surrounding 10-Mb chromosomal area plotted as a grayscale image. Grayscale levels are adjusted relative to the maximum centimorgan value in the 90th percentile and values above that level set to black; correspondence between gray levels and cM is indicated at the top of each panel. Red line: smoothed ext_AUC _values were down-sampled before plotting. The left-hand y-axis labels refer to percentile levels of the PE_mat _data, and the right-hand y-axis labels are for the line plot of ext_AUC _values.

To define such 'blocks' of similar ext_AUC _values, we developed peak detection and processing functions for hzAnalyzer, by which we detected peaks in each sample population's ext_AUC _values and then merged together adjoining peaks that had similar peak characteristics (see Materials and methods). To extract and analyze genomic regions with a higher likelihood of having been influenced by population historical events (that is, natural selection, migration, population bottlenecks, and so on), we extracted a set of outlier peaks that possessed extreme peak height, and we then merged together neighboring outlier peaks that were not well separated from one another into a set of outlier peak regions (see Materials and methods). Table 2 shows the peak counts after the different peak detection and merging steps (see Materials and methods) that are illustrated in Figure 3d. Statistics for ten of the top peak regions for each population are shown in Table 3, while statistics for all outlier peaks and peak regions are presented in Tables S3a-d and S4a-d in Additional file 2, respectively. To visually examine some of the most prominent regions within the genome, the 10-Mb areas surrounding two of the top autosomal outlier peak regions from each population are plotted in Figure 4, with PE_mat _(cM) values plotted in grayscale and smoothed ext_AUC _values as a superimposed line; Additional files 5, 6, 7 and 8 provide lower resolution genome-wide plots for each population.

**Table 2:** Genome-wide peak counts at different stages of peak processing

Peak dataset type	YRI	CEU	CHB	JPT
Complete peaks	25,723	25,142	25,413	25,418
Merged peaks	15,325	15,815	16,117	16,119
Outlier peaks	873	908	1,007	1,047
Outlier peak regions	349	358	401	416

**Table 3:** Examples of top outlier peak regions for each population

**Pop**.	Chr	Position	Low valley	High valley	**Pk.Ht**.	SNPct	W(bp)	W(cm)	Extent_min_	Freq_hap-max_	Top 5 gene(s)	GeneCt
YRI	11	48,984,887	46,179,339	51,434,161	0.7583	2,260	5,254,822	7.1256	1,944,689	0.2966	*BC142657*, *C11orf49*, *AMBRA1*, *PTPRJ*, *CKAP5*	48
	X	64,291,347	62,718,942	66,950,662	0.5043	1,283	4,231,720	4.8877	1,706,793	0.2045	*AR*, *MTMR8*, *ARHGEF9*, *HEPH*, *MSN*	12
	19	21,348,594	20,644,142	21,607,663	0.476	728	963,521	2.0049	278,309	0.4492	*ZNF431*, *ZNF714*, *ZNF708*, *ZNF430*, *ZNF429*	8
	15	42,487,755	40,203,270	42,922,650	0.4277	1,542	2,719,380	4.4896	280,917	0.6695	*FRMD5*, *TTBK2*, *UBR1*, *CASC4*, *TP53BP1*	47
	13	56,734,306	54,206,759	58,262,174	0.3587	3,933	4,055,415	5.588	543,152	0.4322	*PCDH17*, *PRR20*	2
	1	172,725,722	171,321,975	173,400,872	0.3306	1,303	2,078,897	2.7759	1,077,265	0.2288	*RABGAP1L*, *SLC9A11*, *TNN*, *KLHL20*, *RC3H1*	16
	3	49,131,477	46,848,671	51,894,338	0.3307	1,993	5,045,667	6.1529	646,401	0.3729	*DOCK3*, *MAP4*, *SMARCC1*, *CACNA2D2*, *RBM6*	108
	14	66,177,344	65,574,279	67,039,563	0.3157	901	1,465,284	2.1116	451,154	0.3814	*GPHN*, *MPP5*, *FAM71D*, *C14orf83*, *EIF2S1*	7
	2	62,928,490	62,640,536	64,139,379	0.3138	864	1,498,843	1.9062	665,460	0.3898	*LOC51057*, *EHBP1*, *VPS54*, *UGP2*, *MDH1*	7
	16	34,336,349	34,040,995	35,126,826	0.2836	446	1,085,831	1.8457	407,250	0.3559	No overlapping gene symbols	0
CEU	X	56,905,680	54,032,865	58,499,973	2.2637	1,588	4,467,108	7.0248	2,146,728	0.6705	*FAAH2*, *WNK3*, *FAM120C*, *PFKFB1*, *PHF8*	30
	4	33,984,210	32,226,578	34,781,662	1.1807	1,754	2,555,084	3.6574	1,063,099	0.5847	No overlapping gene symbols	0
	10	74,474,938	73,363,734	76,877,918	1.1538	1,994	3,514,184	4.2036	1,225,977	0.5847	*ADK*, *CBARA1*, *MYST4*, *CCDC109A*, *VCL*	45
	17	56,029,088	54,786,548	56,697,157	1.1855	950	1,910,609	2.8676	650,495	0.7542	*BCAS3*, *USP32*, *TMEM49*, *APPBP2*, *CLTC*	17
	11	47,998,372	46,181,418	51,434,161	1.021	2,256	5,252,743	7.1227	2,476,725	0.3898	*BC142657*, *C11orf49*, *AMBRA1*, *PTPRJ*, *CKAP5*	48
	2	136,299,164	134,916,186	137,368,521	0.8663	2,261	2,452,335	2.5254	983,710	0.7373	*ZRANB3*, *TMEM163*, *R3HDM1*, *RAB3GAP1*, *DARS*	13
	12	33,861,148	32,686,226	38,560,061	0.8322	3,319	5,873,835	10.0596	1,347,796	0.3898	*CPNE8*, *KIF21A*, *SLC2A13*, *PKP2*, *C12orf40*	12
	6	28,454,924	26,007,118	30,140,896	0.829	4,884	4,133,778	5.9709	1,925,294	0.1102	*AK309286*, *GABBR1*, *ZNF322A*, *ZNF184*, *TRIM38*	116
	1	35,416,859	35,088,324	36,679,373	0.8304	716	1,591,049	1.9898	957,224	0.6525	*ZMYM4*, *EIF2C3*, *KIAA0319L*, *THRAP3*, *EIF2C4*	27
	15	41,082,380	40,198,534	43,641,969	0.8478	2,088	3,443,435	5.6849	620,196	0.5424	*FRMD5*, *TTBK2*, *UBR1*, *CASC4*, *TP53BP1*	60
CHB	X	65,578,703	62,500,211	68,093,466	5.0603	1,811	5,593,255	6.4603	3,865,015	1	*OPHN1*, *AR*, *MTMR8*, *ARHGEF9*, *HEPH*	16
	16	46,816,951	45,019,628	47,582,293	1.7237	1,197	2,562,665	4.3969	1,239,919	0.5682	*ITFG1*, *PHKB*, *LONP2*, *FLJ43980*, *N4BP1*	16
	3	49,185,837	46,688,461	52,084,708	1.7352	2,120	5,396,247	6.5804	1,411,092	0.7614	*DOCK3*, *MAP4*, *SMARCC1*, *CACNA2D2*, *RBM6*	124
	20	33,906,145	31,887,721	34,457,027	1.1757	1,515	2,569,306	4.7755	641,849	0.6477	*PHF20*, *ITCH*, *PIGU*, *NCOA6*, *UQCC*	44
	17	56,257,611	54,786,430	56,802,151	1.0563	1,040	2,015,721	3.0254	563,530	0.7159	*BCAS3*, *USP32*, *TMEM49*, *APPBP2*, *CLTC*	17
	1	50,585,736	48,934,817	53,018,060	1.0833	2,299	4,083,243	5.1065	1,010,684	0.5227	*AGBL4*, *FAF1*, *ZFYVE9*, *OSBPL9*, *EPS15*	25
	2	72,358,317	72,139,001	73,325,161	1.0369	914	1,186,160	1.5085	785,525	0.8523	*EXOC6B*, *SFXN5*, *RAB11FIP5*, *EMX1*, *CYP26B1*	10
	15	61,947,157	61,420,087	63,327,879	1.0094	1,241	1,907,792	3.1497	757,196	0.6136	*HERC1*, *CSNK1G1*, *ZNF609*, *DAPK2*, *USP3*	27
	5	43,332,643	41,569,404	46,432,729	0.9655	3,211	4,863,325	7.1409	940,407	0.3182	*HCN1*, *GHR*, *OXCT1*, *NNT*, *MGC42105*	18
	4	33,761,093	32,224,418	34,856,502	0.9703	1,854	2,632,084	3.7676	705,955	0.4773	No overlapping gene symbols	0
JPT	X	65,574,377	62,541,924	68,119,789	4.6884	1,830	5,577,865	6.4425	2,974,147	1	*OPHN1*, *AR*, *MTMR8*, *ARHGEF9*, *HEPH*	16
	3	49,116,120	46,984,286	52,086,091	1.5554	1,926	5,101,805	6.2214	1,411,982	0.6744	*DOCK3*, *MAP4*, *SMARCC1*, *CACNA2D2*, *RBM6*	117
	16	46,405,964	45,019,628	47,589,442	1.4543	1,203	2,569,814	4.4092	1,013,458	0.6512	*ITFG1*, *PHKB*, *LONP2*, *FLJ43980*, *N4BP1*	16
	11	51,354,159	45,478,264	51,434,161	1.1994	2,899	5,955,897	8.0762	1,844,210	0.3837	*BC142657*, *C11orf49*, *AMBRA1*, *PHF21A*, *PTPRJ*	57
	1	50,634,574	49,236,023	52,884,998	1.1586	1,797	3,648,975	4.5634	727,447	0.7442	*AGBL4*, *FAF1*, *ZFYVE9*, *OSBPL9*, *EPS15*	22
	20	33,684,569	31,842,856	34,417,872	1.0984	1,512	2,575,016	4.7861	982,049	0.4186	*PHF20*, *ITCH*, *PIGU*, *NCOA6*, *UQCC*	45
	2	72,358,024	71,951,517	73,081,576	1.0483	956	1,130,059	1.4372	771,138	0.8721	*EXOC6B*, *SFXN5*, *EMX1*, *CYP26B1*, *SPR*	5
	15	62,853,070	61,443,788	63,335,076	1.0036	1,219	1,891,288	3.1224	868,839	0.5465	*HERC1*, *CSNK1G1*, *ZNF609*, *DAPK2*, *USP3*	27
	14	65,950,544	65,443,441	67,097,562	0.9667	1,112	1,654,121	2.3838	987,976	0.407	*GPHN*, *MPP5*, *C14orf83*, *FAM71D*, *PLEKHH1*	9
	17	55,887,456	53,750,682	56,801,264	0.9595	1,451	3,050,582	4.5786	555,447	0.8023	*BCAS3*, *PPM1E*, *USP32*, *TEX14*, *TMEM49*	33

Outlier peak regions were sorted by peak height and ten representative examples chosen from the top. Columns with abbreviated names not referred to in the text are: Pop., population; Chr, chromosome; Pk. Ht, peak region height; SNPct, number of SNPs in region; W(bp), peak region width in base pairs; W(cm), peak region width in centimorgans; GeneCt, number of genes in region. Genes listed are sorted by length and the top five shown.

**Figure 5:**
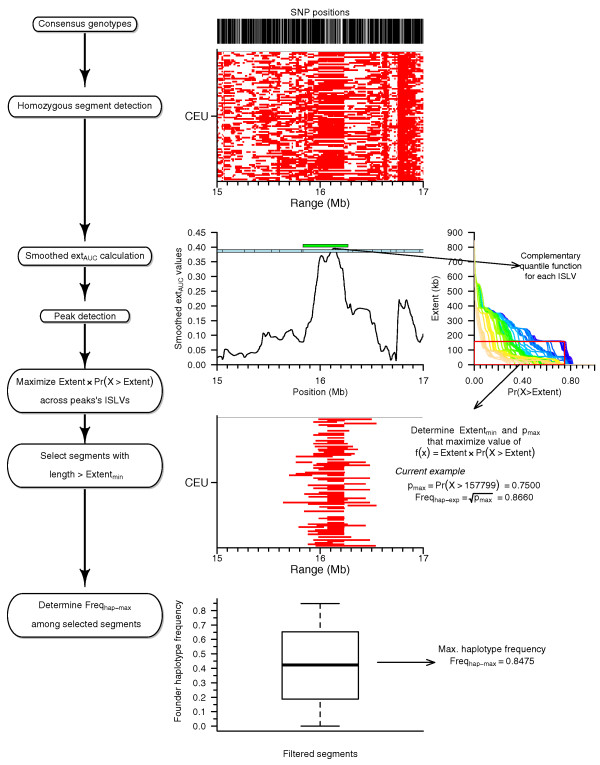
**Diagram of the method for comparing the extent and frequency of homozygous segments with haplotypes underlying ext_AUC _peaks**. From a consensus set of genotypes, homozygous segments were detected, ext_AUC _calculated and smooth spline interpolation performed, and peaks detected. Complementary quantiles (*Pr*(*X *>*Extent*) = (1 - *Pr*(*X *≤ *Extent*)) = (1 - Percentile/100)) were calculated from each ISLV's percentile values underlying a particular peak. Values of *Extent *and *Pr*(*X *>*Extent*) that maximized the value of *Extent *× *Pr*(*X *>*Extent*) were extracted as parameters *Extent_min _*and *p_max_*, segments with length greater than *Extent_min _*selected, and the maximum founder haplotype frequency (*Freq_hap-max_*) obtained across those segments. The expected haplotype frequency (*Freq_hap-exp_*) was calculated as the square-root of *p_max_*.

**Figure 6:**
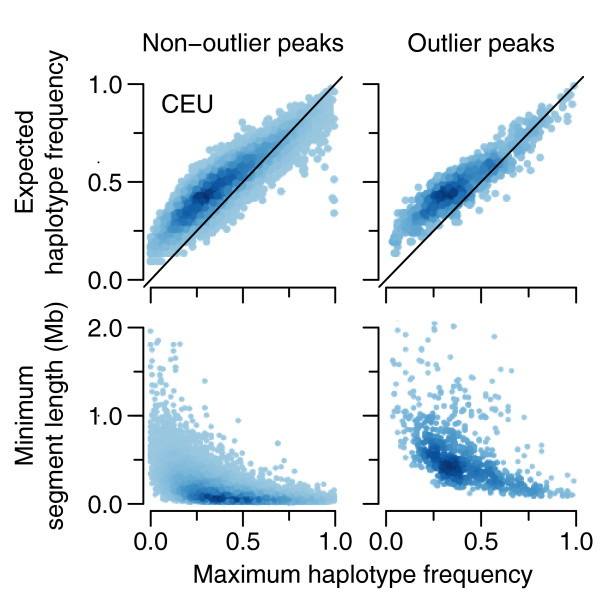
**Comparison of the extent and frequency of homozygous segments with founder haplotypes underlying ext_AUC _peaks**. Minimum segment length (*Extent_min_*), expected haplotype frequency (*Freq_hap-exp_*), and maximum haplotype frequency (*Freq_hap-max_*) were calculated as diagrammed in Figure 5 for peaks dichotomized into non-outlier and outlier peaks. Data points were colored using a two-dimensional density estimate using R's function densCols with nbin = 1,024. Data shown are for CEU with plots for all populations in Additional file 9.

To confirm that peaks, which were detected based on the structure of contiguous homozygosity, were consistent with the frequency and extent of underlying haplotypes, we developed an analytical approach that is diagrammed in Figure 5 (see Materials and methods). Using that approach, we compared three values for each peak: the minimum segment length threshold (*Extent_min_*), the expected haplotype frequency (*Freq_hap-exp_*), and the maximum founder haplotype frequency (*Freq_hap-max_*). The top panels of Figure 6 plot the expected and observed maximum haplotype frequencies for CEU, with peaks dichotomized into non-outlier and outlier peak groups (for all populations, see Additional file 9). For both peak groups, *Freq_hap-max _*and *Freq_hap-exp _*are strongly correlated (0.8863 <*r *< 0.9237 for all populations; Pearson's correlation coefficient), but the slope and intercept of the linear regression (intercept = 0.2110, slope = 0.7162 for CEU non-outliers) indicate that *Freq_hap-max _*values are lower than expected for peaks with values of *Freq_hap-exp _*less than about 0.6. Thus, for those peaks, homozygous segments with length exceeding *Extent_min _*tend to originate more frequently from multiple low-to-intermediate frequency haplotypes. However, peaks with expected frequency >0.6 appear to cluster closer to the unit line and therefore may tend to originate more often from a single higher frequency haplotype. The lower panels in Figure 6, which plot *Extent_min _*versus *Freq_hap-max_*, show that outlier peaks, representing high-ranking ext_AUC _values, tend to harbor longer, higher frequency haplotypes compared to non-outlier peaks. These results provide evidence that our peak detection and processing methods are capable of defining regions of locally restricted haplotype diversity.

**Figure 7:**
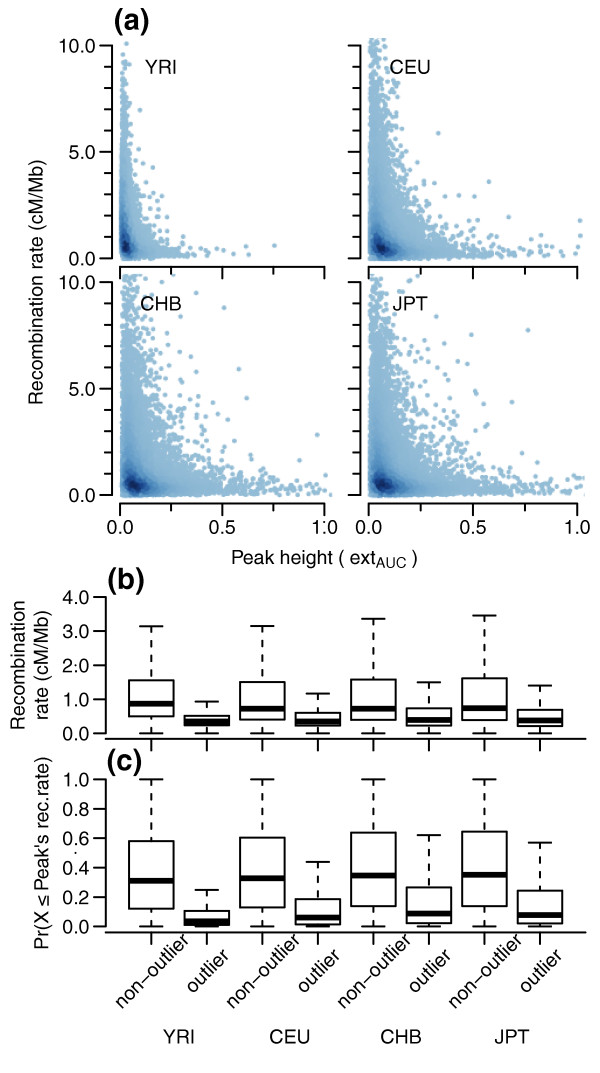
**Genomic regions of high-frequency extended homozygosity possess low recombination rates**. Recombination rate was calculated for the central half-width of each autosomal peak in the merged peak dataset using population-specific genetic maps based on the 1000G pilot data. **(a) **Recombination rate versus ext_AUC _peak height. **(b,c) **Boxplot summaries for non-outlier and outlier peaks for each sample population for (b) recombination rate, and (c) recombination rate transformed into cumulative probabilities and adjusting for peak width.

A related question to haplotype frequency and extent is that of local variation in recombination rate and its impact on the structure of contiguous homozygosity. We used the 1000 Genomes Project pilot data genetic map [22] to calculate population-specific genetic distance and recombination rate across each peak (see Materials and methods). Figure 7a indicates a negative correlation between ext_AUC _peak height and recombination rate (Spearman's rank correlation test rho: -0.14, -0.21, -0.18, -0.19 for YRI, CEU, CHB, and JPT, respectively) and shows that most peaks possessed both low ext_AUC _values and low recombination rates (approximately 1 cM/Mb). While peaks with the highest recombination rates also tended to have low ext_AUC _values, peaks with higher ext_AUC _values displayed much lower recombination rates. These results agree with recent analyses that showed that observable recombination events occur within only a small proportion of the genome [5,22]. Figure 7b makes the difference more clear; genomic regions possessing higher frequency/extended haplotypes (outlier peaks) generally possess much lower recombination rates than small peaks made up of shorter, more heterogeneous haplotypes (non-outlier peaks). Figure 7c, with recombination rates transformed into cumulative probabilities while accounting for peak width (see Materials and methods), confirms that this difference is not simply an indirect association due to peak width differences between the two groups. To calculate coverage by low recombination rate outlier peaks, we selected outlier peaks that had very low recombination rates (rates below the peak width adjusted 25th percentile). The percentage of outlier peaks accounted for by low recombination rates was 74 to 89%, with autosomal coverage of 113.5, 126.7, 130.5, and 139.1 Mb for YRI, CEU, CHB, and JPT, respectively.

#### Population differentiation in genomic regions with high-ranking ext_AUC _values

We then examined how regions of high-ranking ext_AUC _values overlap between the four populations. Within the coordinates of each peak in the dataset, we calculated the maximum value rank for each of the four population's ext_AUC _values. Considering that outlier peaks represent ext_AUC _values with ranks above approximately 0.85 to 0.90, then Additional file 10 shows that the majority (>50%) of outlier peaks in one population intersect with similarly high-ranking ext_AUC _values in the other groups, with more than 75% of outlier peaks in JPT and CHB intersecting with high-ranking ext_AUC _values in the other East Asian population (see Materials and methods).

**Figure 8:**
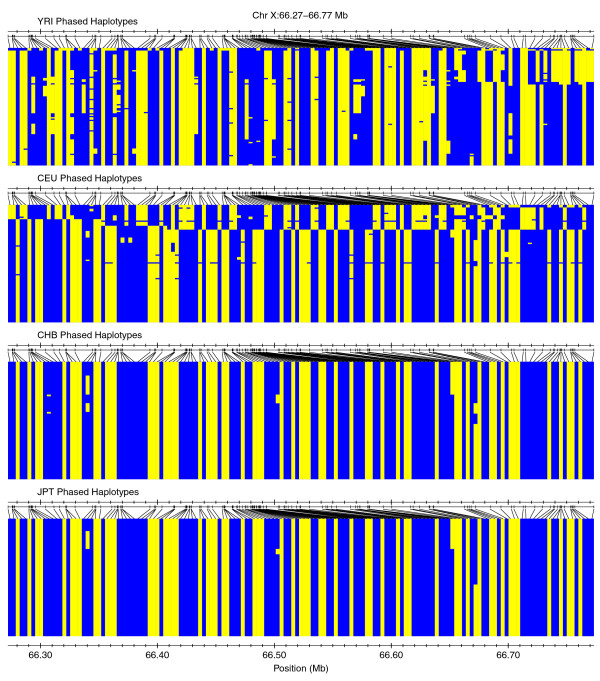
**Peaks with high cross-population homozygosity and high Fst/θ identify genomic regions with dissimilar haplotype structure**. Outlier peaks for each base population were chosen, filtered to include those with high ranking ext_AUC _across all four populations, and sorted based on average Fst/θ between the base population and the other three populations (or with YRI and CEU if the base population was CHB or JPT). Phased haplotypes were plotted for an example peak for YRI with high differentiation (mean Fst/θ = 0.83).

**Figure 9:**
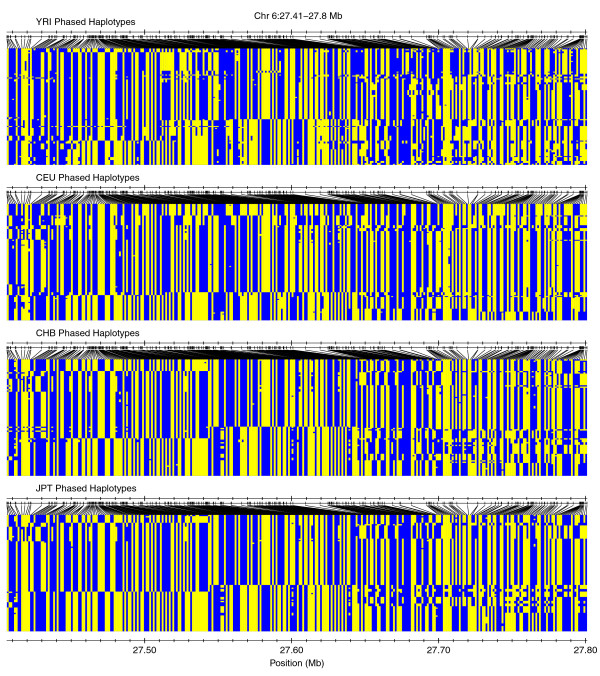
**Peaks with high cross-population homozygosity and low Fst/θ identify genomic regions with similar haplotype structure**. Outlier peaks for each base population were chosen, filtered to include those with high ranking ext_AUC _across all four populations, and sorted based on average Fst/θ between the base population and the other three populations (or with YRI and CEU if the base population was CHB or JPT). The structure of phased haplotypes is plotted for an example peak for JPT with low differentiation (mean Fst/θ = 0.0235).

We then posited that if outlier peaks that overlapped with high-ranking ext_AUC _values in multiple populations were annotated with a measure of population differentiation such as Fst/θ [24,25], then we could identify regions of the genome that are similar or dissimilar for intermediate to high-frequency long ('extended') haplotypes between populations. To illustrate how this combination might be useful for interrogating underlying haplotype structure, we compared phased haplotypes for two different peak categorizations: peaks possessing both high ranking ext_AUC _values and high average Fst/θ between populations (abbreviated as a high/high peak); and peaks with high-ranking ext_AUC _values but low average Fst/θ (a high/low peak). Figure 8 shows a YRI high/high peak at Chr X:66.27-66.77 Mb (mean Fst/θ between other groups and YRI = 0.83) for which most major alleles are completely opposite between the base YRI population and the other three populations. In contrast, the high/low JPT peak at Chr 6:27.41-27.8 Mb (mean Fst/θ with JPT = = 0.0235) in Figure 9 displays a broadly similar haplotype structure across all populations. Two examples of high/high outlier peak regions (Chr X:62.7-67 Mb, mean Fst/θ with YRI = 0.6726; Chr 14:65.4-67 Mb, mean Fst/θ with CEU = 0.3585) are presented in Additional file 11.

Based on those observations, we then used this method to search for a set of the longest genomic regions possessing high haplotype frequency differences between the two East Asian populations, which have often been considered similar enough to combine for analytical purposes. We selected peaks that had both high-ranking ext_AUC _values in the two groups as well as extreme Fst/θ values. Across the set of 70 JPT and 70 CHB peaks shown in Table S5a,b in Additional file 2, there was an average haplotype frequency difference of 15 ± 5% (mean ± SD%) between CHB and JPT. Additional file 12, which shows the top five peaks (after sorting by the proportion of extreme Fst/θ value loci) for CHB and JPT, indicates that the observed structure of phased haplotypes tends to agree with the estimated haplotype frequency differences in Table S5a,b in Additional file 2. For example, the first plot for Chr 1:187.22-187.85 Mb spans 377 loci (minor allele frequency (MAF) >0.01 in JPT or CHB) and shows two distinct haplotypes with an estimated 0.20 frequency difference that extends across the whole 600-kb window. The top JPT region at Chr 22:39.04-39.11 Mb is a region with only 32 SNPs (MAF >0.01 in JPT or CHB), but 83% of them have extreme Fst/θ values. One background haplotype can be seen to increase in frequency from 0.21 in CHB to 0.45 in JPT, representing an estimated 0.23 frequency difference for this region. Such frequency differences may reflect natural selection but also may represent the effects of random genetic drift in allele frequencies since ancestral Japanese populations migrated from the Asian continent.

These results show that detection of outlier peaks/peak regions using hzAnalyzer in conjunction with measures of population differentiation can be used for extracting genomic regions with substantially similar or dissimilar haplotype structure between sample populations from high-density genotyping datasets.

#### Genomic regions containing areas at or approaching fixation

Figure 4 (for example, left panel of CEU, right panels of CHB and JPT) and Additional files 5, 6, 7 and 8 display some peaks that extend across all or almost all percentiles (from the 100th down to the 0th) in PE_mat_, representing genomic regions that are homozygous across all or almost all samples within a population and for which a single haplotype may be at or near fixation. Such regions may represent the impact of past natural selection in the human population, by which selected mutations have been driven to high frequency and the surrounding polymorphims have 'hitch-hiked' [26] as a haplotype to higher frequency. Eventually, other forces such as genetic drift may finally reduce other variation, leaving just a single haplotype to predominate.

**Figure 10:**
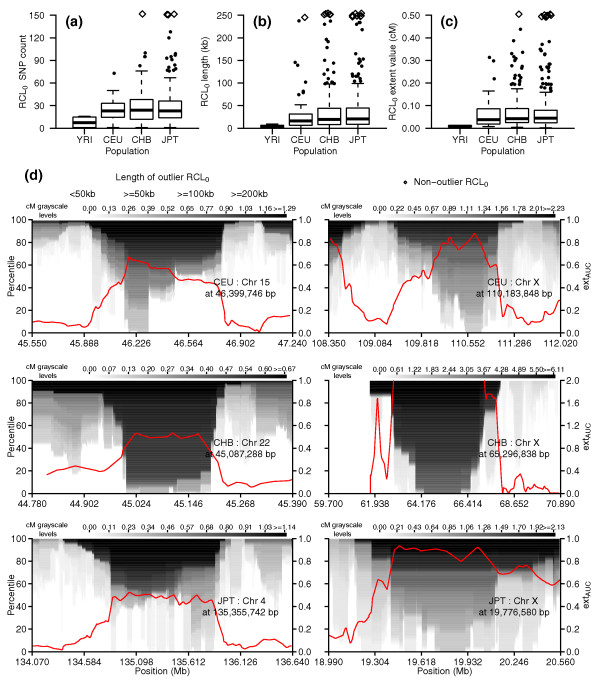
**Peak regions harbouring areas at or approaching fixation**. **(a-c) **Boxplots by population: (a) RCL_0 _SNP counts; (b) RCL_0 _length (kb); (c) RCL_0 _extent value (cM). **(d) **One each of the top autosomal and top chromosome X candidate fixation peak regions from Table S7a-c in Additional file 2 for each population, and the centimorgan values from PE_mat _for the chromosomal area ± 1 peak region width from the center plotted as a grayscale image. Grayscale levels are adjusted relative to the maximum value in the 90th percentile and values above that level set to black; legend key between gray levels and cM is indicated at the top of each panel. Red line: smoothed ext_AUC _values were down-sampled at a 10% frequency and plotted. The left-hand y-axis labels refer to percentile levels of the PE_mat _data, and the right-hand y-axis labels are for the line plot of ext_AUC _values. Outlier points in (a-c) are randomly jittered to reduce overlap and datapoints off the figure plotted as diamonds.

To estimate the extent of fixed regions throughout the genome for each population, we searched PE_mat _for runs of consecutive loci that had measurable homozygous extent in the 0th percentile level (RCL_0_). Figure 10a-c shows the distributions of SNP count, run length (kb), and run extent value (cM) within detected RCL_0_. Based on the first quartile (Q1) of RCL_0 _SNP count and run extent values in Figure 10a,c, we extracted longer RCL_0 _to define a set of candidate fixed areas (see Materials and methods). Summary statistics for the RCL_0 _in the candidate fixed areas are shown in Table 4, while their coordinates are provided in Table S6a-d in Additional file 2. Of the genomic regions of extreme homozygosity that we earlier defined as outlier peak regions, 8, 29, and 40 contained candidate fixed areas in CEU, CHB, and JPT, respectively; the regions that intersect with candidate fixed areas are shown in Table S7a-c in Additional file 2. We picked one example each from among the top autosomal and chromosome X candidate fixation peak regions for each population and plot the PE_mat _and ext_AUC _values in Figure 10d. Besides showing the high/high peak example described earlier, Figure 8 also shows the phased haplotypes in one of the most extreme candidate fixation regions around Chr X:65.3 Mb. Readers can use the plotting functions built into hzAnalyzer [21] if they wish to examine phased haplotype structure in HapMap samples for additional regions in Tables S6 and S7 in Additional file 2, or for other genomic coordinates.

**Table 4:** Summary of RCL_0 _in candidate fixed areas

	Minimum			Maximum			Total			
			
**Pop**.	SNP count	Extent (cM)	Extent (bp)	SNP count	Extent (cM)	Extent (bp)	Run count	SNP count	Extent (cM)	Extent (bp)
YRI	14	0.0128	9,359	14	0.0128	9,359	1	14	0.0128	9,359
CEU	17	0.0188	5,675	73	0.3135	258,163	21	711	2.1398	1,410,226
CHB	13	0.0252	8,033	716	3.0799	1,798,574	139	5,771	16.1761	9,815,425
JPT	15	0.0247	8,544	385	2.0276	872,011	205	8,589	24.9607	13,597,432

Pop., population.

We compared the overlap of our candidate fixed areas and regions with data from five earlier reports that searched for genomic regions that had evidence for positive selection resulting in a selective sweep or that appeared differentially fixed in one population versus another [5,27-30] (Tables S6 and S7 in Additional file 2). To ease examination of the correspondence and overlap between our results and those of other studies, we created a combined set of fixation candidate regions by resolving any overlap between the three sample populations' candidate fixed regions and present those in Table S8 in Additional file 2. This set represents much larger genomic regions compared to the RCL_0 _candidate fixed areas, with genome-wide coverage of 84 Mb for this set versus approximately 11 Mb of RCL_0 _that are embedded within these larger regions. Over half of the candidate fixed areas did not intersect directly with known coding regions, which is in keeping with previous reports that searched for regions experiencing positive selection [28,31]. However, summarization across Table S8 in Additional file 2 shows that over 50% of the combined regions had RCL_0 _that intersected at least one coding region.

Of the combined fixation candidate regions, 87.5% overlapped with regions from at least one of the other studies. Overall, 39.3% of the combined regions overlap Sabeti *et al*.'s [28] results (Sabeti, n = 255), while 85.1% overlap with the Kimura *et al. *[27] report (Kimura, n = 1,379; no chromosome X data). Similar to the Sabeti *et al. *comparison, the regions from Tang *et al. *[29] intersected 44.7% of the combined regions (Tang, n = 604; no chromosome X data), but only 5.4% of our regions intersected those in O'Reilly *et al. *[30] (O'Reilly n = 60).

#### Extending hzAnalyzer analyses to case-control association studies

In addition to the population genetic analyses described above, we have recently developed a methodology that we term agglomerative haplotype analysis (AHA) for detecting genomic regions that possess increased or decreased levels of contiguous homozygosity in a case-control association study approach [32]. For the current version of hzAnalyzer, we provide a supplementary R script on the hzAnalyzer web-site along with a tutorial for performing a comparison between two groups of samples [21]. For researchers wishing to implement these methods, we recommend that population stratification needs to be controlled either through appropriate statistical methods [33] or by genetically matching case and control samples [34]. For the following example, we will use the JPT and CHB population samples as proxies for a real case-control dataset.

**Figure 11:**
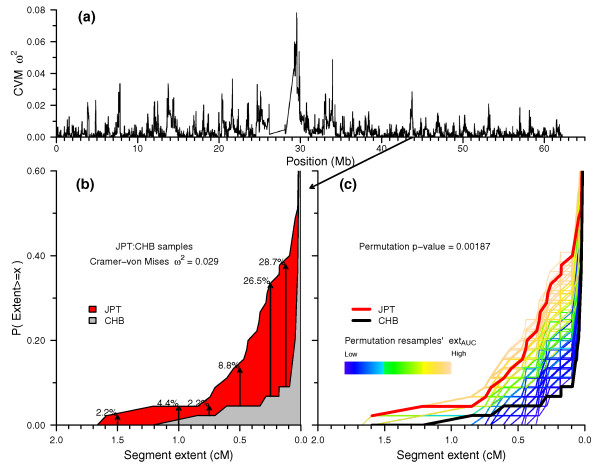
**Agglomerative haplotype analysis: testing for differences between two population samples' homozygous segment length distributions**. We compared the homozygous segment length distributions between JPT and CHB at each locus position in the chromosome 20 masked ISLM_cm _matrix by calculating the Cramer-von Mises two-sample minimum distance statistic ω^2^. A representative position (Chr 20:43.82 Mb) with a high ω^2 ^value was chosen to illustrate the AHA permutation testing procedure. **(a) **ω^2 ^values between JPT and CHB for chromosome 20. **(b) **Segment length ECDFs for JPT and CHB at example peak ω^2 ^position. **(c) **Determination of significance level using permutation test: ECDFs from 100,000 resamples after mixing JPT and CHB data and randomly reassigning group labels. ω^2 ^was calculated for each permutation resample, and the achieved significance level calculated as the number of permutations with ω^2 ^values greater than or equal to the original two-sample ω^2 ^divided by the total number of permutations.

The basis of the AHA method is minimum-distance estimation between two population samples' homozygous segment length distributions at each SNP position. We calculate a version of the two-sample Cramer-von-Mises test statistic ω^2 ^(CVM ω^2^; see Materials and methods) [35-37]; ω^2 ^values for JPT and CHB on chromosome 20 are shown in Figure 11a. Figure 11b shows JPT and CHB segment length ECDFs to visualize the difference between the two sample distributions for an example peak position. The CVM ω^2 ^in Figure 11b provides a measure of the difference between the two distributions, but since normal asymptotic methods are not available for this application, we assess the significance of that difference using a standard permutation test procedure. In Figure 11c, we illustrate the results of calculating CVM ω^2 ^for each of 100,000 resamplings after swapping sample population labels. Implementing the AHA method for actual case-control data involves a number of additional steps that exceed the scope of the current HapMap dataset that is used in this report.

## Discussion

The similarity and dissimilarity of genetic variation between different human populations has been an important ongoing question of genetic analysis [38,39], with recent research addressing how specific populations that have historically been considered relatively homogeneous might still harbor important hidden heterogeneity [14,40-43]. Such questions also tie directly into the topic of relatedness and to what extent haplotypes are shared between individuals within a population compared to between populations. As we have shown here, analysis of contiguous homozygosity can provide a means for estimating the extent and approximate frequency of such shared haplotypes that are present within human population samples. hzAnalyzer provides researchers with a new set of computational tools for performing comprehensive analyses of contiguous homozygosity in high-density genotyping datasets.

We conclude that hzAnalyzer's detection method proved more appropriate than PLINK's for detecting complete autozygous segments and that our algorithm may be less likely to call regions with low but real heterozygosity as homozygous segments. However, we should note that more time spent optimizing PLINK's settings might produce better overlap between the two datasets, although default settings are those that are most likely to be used by normal users. We also note that hzAnalyzer provides the user with finer control over their choices for calling segments across regions with heterogeneous levels of heterozygosity and inter-SNP gaps (see Materials and methods and hzAnalyzer tutorials).

In addition, a number of ROH analyses [6-13,15] have been reported that did not include freely available software for use by outside researchers. Of those, that of Curtis *et al. *[9] is the only one that we know of that included genome-wide graphical output upon which we could base any comments for comparing underlying ROH detection methodologies. Because of their choice not to define any SNP density or inter-SNP gap thresholds, the figures in Curtis *et al. *impart the impression that all centromeres and large gaps possess very high-frequency contiguous homozygosity extending across large genomic distances. However, we do not feel that the amount of information present at those gap's edges supports such conclusions. Curtis *et al. *mentioned that setting a criterion for marker density would have had the effect of excluding the centromeres from analysis. However, our hzAnalyzer results show that it is possible for a detection algorithm to allow segments with a higher likelihood of being truly homozygous to span such large gaps while reducing false positive 'extended' homozygous segments caused by small numbers of homozygous SNPs at a gap's edges.

While we identified a general agreement between detected autozygous segments and low-coverage sequencing data from 1000 Genomes Project (Figure 7), subsequent analyses comparing larger numbers of samples between microarray and next-generation sequencing data would help add to our understanding of how to perform more accurate analyses in structurally heterogeneous regions of the genome. We posit that comparisons of ROH and autozygous segments with genotype data from both low-coverage and high-coverage sequencing data may provide a better means for adapting hzAnalyzer parameters to local, rather than global, error rates (that is, heterozygosity thresholds, SNP density).

In examining and quantifying contiguous homozygosity, chromosome X stood out as possessing much longer homozygous segments compared to similarly sized autosomes, representing higher frequency extended haplotypes and genomic regions with decreased diversity. The different biology and evolutionary history of chromosome X has previously been noted [44], with specific differences reported for mutation rate [45-48], LD [4,5,49], and natural selection [50]. Such differences between autosomes and chromosome X likely relate to the latter's haploid/hemizygous status in human males, wherein natural selection has had a greater chance to work on both beneficial and deleterious recessive mutations [48,50]. On chromosome X, we feel that discussion of positive selection flows naturally into the topic of haplotype fixation, which is perhaps the most extreme result of positive selection, in which a selected variant and the region (haplotype) around it rise in frequency in the population [51] to the point that one single haplotype accounts for all or almost all of that region's genetic variation.

Of all chromosomes, chromosome X was the largest single contributor to the list of combined fixation candidate regions, with 9 (16%) out of the 56 unique non-overlapping regions detailed in Table S8 in Additional file 2. As such, these regions all had high ext_AUC _values and very long extent in at least one of CEU, CHB, or JPT. Six of these regions overlapped with those detected in previous studies: five in Sabeti and colleagues' dataset [5,28] and one in O'Reilly *et al. *[30]. The three regions that appear novel for this report reside at 104.2 to 105.4 Mb, 113.7 to 114.4 Mb, and 126.1 to 127.7 Mb; phased haplotype plots can be found in Additonal file 13. The last listed region appears especially interesting, as in Additional file 13 one can see that it contains a broad region (approximately 1 Mb wide) of loci near or at fixation, but this region contains only a single small candidate gene, which, due to its function, makes an interesting potential target of natural selection. The actin-related protein T1 gene (*ACTRT1*), is only 1,437 bp long, consists of a single exon and is a major component of the calyx, which is a key structure in the perinuclear theca of the mammalian sperm head [52]. Interestingly, one report showed that while many sperm-expressed proteins appear to be evolving more rapidly on chromosome X versus those on autosomes, *ACTRT1 *appeared in the lower end of the spectrum of amino acid substitution rates [53], which suggests that the gene is under strong selective constraint due to the key structural role that this protein plays in sperm function.

One of the combined regions that overlapped the most with the earlier reports contains the exocyst complex component 6B gene (*EXOC6B*; Chr 2:71.9-73.1 Mb), the region of which was reported as a top target of positive selection by Sabeti *et al. *[28] (although the gene was not listed) and included by Kimura *et al. *[27] and Tang *et al. *[29] in supplementary tables (Kimura *et al.*, gene then known as *SEC15L2*; Tang *et al.*, gene listed was the neighboring small *CYP26B1 *gene) [27-29]. *EXOC6B*, a component of the mammalian exocyst complex, participates in active exocytosis [54], and the original discovery report suggested that it may play a role specific to exocyst complexes in nerve terminals [55]. *EXOC6B *has one of the highest peak ext_AUC _values across autosomes in JPT, the highest base-pair coverage by RCL_0 _for any autosomal fixed area, and phased haplotype plots show that it is also nearly fixed in CHB (Additional file 13). Interestingly, that plot shows that the same extended haplotype is at high frequency in CEU, but that YRI possesses the remnant of another very different extended haplotype in this gene. This observation suggests that this gene may have experienced differential selective events during human history.

In comparing our combined fixation candidate regions with those in earlier reports, we observed the greatest overlap with that of Kimura *et al*. This greater overlap was likely due to the similarity between the two methodologies, since they both searched for areas of the genome that are essentially monomorphic in each target population. However, while their method searched for regions that were fixed in a single target population by using another population as reference, the current report's methods made no such explicit comparisons. Nevertheless, our use of HPS_ex _to filter segments prior to calculating ext_AUC _and creating PE_mat _placed an implicit target/reference structure on downstream analyses. In order for any particular segment to have been considered informative enough to include in an analysis such as candidate fixed area detection, then at least one population of those examined must have had homozygosity frequency (Freq_HOM_) values that were informative across enough loci that HPS_ex _could satisfy the 0.01 threshold. The use of HPS_ex _along with our use of JPT and CHB as separate groups (while Kimura *et al. *[27] had combined them) allowed us to detect candidate regions of population-specific fixation between the two East Asian populations. One can search for other candidate regions by filtering Table S8 in Additional file 2 for 'Pops' that include only one of the two East Asian groups. We should also note that while we detected 366 candidate fixed areas, the Kimura *et al. *report [27] contained 1,379 hits, of which only 120 overlapped with our candidate fixed areas and only 47 with our combined candidate fixation regions. Since the average SNP count of Kimura *et al. *hits (mean SNP count = 12.3) was below the minimum used for extracting all of our candidate fixation areas, the overlap of our set and theirs likely represents the longest and highest confidence hits from their dataset. Resolving the small details of the intersection between our fixation candidates and the external datasets lies beyond the scope of this report.

The recombination rate analysis in Figure 7 showed that outlier peaks possess much lower recombination rates compared to regions that are haplotypically more heterogeneous. This is of potential concern, as regions that lack recombination hotspot motifs [56,57] might mimic signatures of natural selection. Since methods that have been used to detect extended haplotype homozygosity may make assumptions about uniform recombination rates across the genome [58,59], this might lead to higher false positive rates than expected. This supports the necessity of using multiple lines of evidence for identifying signals of positive selection, as has been done in a number of major recent studies [22,28]. Conversely, another recent study [30] showed, using simulation, the profound effect of increasing selection coefficients on the ability of the LDhat [60] recombination inference program to detect recombination. This could impact our perception of local recombination rate estimates and hotspot locations. The potential confounding between the two phenomena may be alleviated as sample populations in addition to YRI, CEU, CHB, and JPT are used for recombination rate and hotspot inference.

While population genetic analyses provide us with knowledge and insight that helps guide our development of practical applications such as genome-wide association studies, it is the latter that will provide a measurable benefit to humankind. The predominant model for such studies has been the common disease/common variant hypothesis [61], but other hypotheses have been proposed, such as the multiple rare variant hypothesis [18,62]. To develop methods for detecting variants under these different assumptions, several researchers have proposed methods for combining information across multiple loci that may be able to distinguish shared haplotype segments or clusters of loci representative of underlying rare variants [18,63-65]. Also, several groups have proposed a version of population-based autozygosity mapping, which considers that rare variants generally are younger and reside on haplotypes that are longer than control haplotypes within the same genomic region [15,17]. Disease susceptibility variants that have a recessive mode of inheritance then may be detectable as regions of increased homozygosity/autozygosity in cases versus controls. In this report, we introduced our AHA methodology for applying contiguous homozygosity analysis to case-control association studies. The next version of hzAnalyzer will incorporate additional functions for AHA-based case-control association analysis, such as genetic sample matching [34], to account for population stratification, and additional permutation-based methods to determine genome-wide significance thresholds.

## Conclusions

We provide a comprehensive analysis of the extent of contiguous homozygosity in the four human population samples of the HapMap Phase 2 dataset using hzAnalyzer, our newly described R package. As we show, the local extent of homozygosity varies greatly between both different regions of the genome and different sampled populations, yet shows similarities between populations as well. hzAnalyzer should prove useful for interrogating the local genomic structure of contiguous homozygosity in comparisons of other worldwide populations, large population samples such as the Japan BioBank [66], and for detection of genomic regions harboring recessive disease variants.

## Materials and methods

### hzAnalyzer

hzAnalyzer is an R [19] package that uses Java classes for detecting runs of contiguous homozygous genotypes and R functions for quantifying the frequency and extent of contiguous homozygosity across the genome and visualizing the results. For genotype processing and analysis, we utilized the R package snpMatrix [67]. hzAnalyzer package version 0.1, which this report describes, can be downloaded from the hzAnalyzer homepage [21] and installed as a local source package into R. For filtering potential erroneous genotypes, we included freely available R code that implements an exact test of Hardy-Weinberg equilbrium [68,69]. hzAnalyzer includes a small built-in example dataset for a single 10-Mb region along with example code for detecting and annotating homozygous segments as well as calculating and plotting ext_AUC _values for one population. In addition, the package provides built-in user-guides that can be accessed using R's help facilities. Those are also available from the hzAnalyzer homepage, which also hosts the R scripts to accompany the user-guides, a single chromosome hzAnalyzer example dataset (hzAnalyzer_example_data.tgz), and a number of supplementary data files. The following sections will briefly describe how we contructed the genotyping dataset that we used to illustrate this program's functions and capabilities, followed by a description of our program, methodology, and analytical workflow. hzAnalyzer's scripts and tutorials provide greater detail about most of the methods that are described in the following subsections.

### Genotype dataset

We constructed a consensus set of genotypes from HapMap Phase 2 release 24 non-redundant autosomal and chromosome X data downloaded from the International HapMap Project [20]. The analyzed data included 30 YRI trios, 30 CEU trios, 45 unrelated CHB, and 44 unrelated JPT samples (one of the original 45 JPT samples had incomplete genotyping data). Our consensus release 24 dataset contained 2,816,866 SNPs after applying both HapMap's and our own set of quality control filters, and selecting SNPs with MAF >0.05. Only genotypes for females were used for chromosome X segment detection. Complete details on contructing this dataset are built into the hzAnalyzer documentation.

#### Chromosomal abnormalities, immunoglobulin regions, and copy-number variable regions

We defined chromosomal abnormalities and copy-number variations (CNVs) based on the results of an in-house algorithm for detecting gross chromosomal abnormalities and copy-number segments, and on gain or loss genotypes from the HapMap Phase III CNV genotype dataset that was downloaded from the HapMap website (file: hm3_cnv_submission.txt, 28-May-2010). Documentation built into hzAnalyzer describes the construction of this dataset.

SNPs were excluded from the consensus dataset if they intersected a gain-loss region that had ≥5% frequency or intersected any of the immunoglobulin variable region (IgV) light and heavy chain regions, which were defined as IGL(Chr 22:20,715,572-21,595,082), IGK(Chr 2:88,937,989-89,411,302), and IGHV(Chr 14:105,065,301-106,352,275). Homozygous segments were masked (set as missing) from analysis if greater than 50% of the included loci intersected chromosomal abnormalities and/or CNVs.

### Detection of homozygous segments

The heuristic algorithm used by hzAnalyzer is a modified version of the one developed for the HapMap Phase 2 paper [5] and was designed to detect homozygous segments while intelligently accounting for inter-SNP gaps as well as a low level of heterozygote 'error' [5,6]. hzAnalyzer's multi-step process for detecting and defining homozygous segments consists of: 1) basic detection of runs of homozygous and heterozygous genotypes; 2) joining of neighbouring homozygous segments across regions of low SNP density; 3) modeling of detected homozygous and heterozygous segments to allow for a low level of heterozygous 'error'; and 4) scan-ahead method to examine neighboring segments with heterogeneous gap and/or heterozygosity structure. User-defined parameter arrays allow control of program behavior, with the ability to vary the minimum segment SNP density and minimum segment length as a proportion of inter-segment gaps based upon gap size ranges. This allows the user to define that very long autozygous segments be allowed to span large gaps (for example, centromeres), but that shorter segments be truncated at the gap edges. hzAnalyzer's default parameter array values were used for this report, except for the lowest values of the gapWidthScanThresholds and gapWidthJoinThresholds. Those gap-width threshold values were determined from the distribution of inter-SNP gaps for the particular chromosome and dataset being used. Inter-SNP gap values were log-transformed, a standard R boxplot summary calculated, and the threshold values calculated as the exponential function of the upper whisker (Third quartile + 1.5 × Inter-quartile-range) value.

In addition, the program is controlled by thresholds for the maximum allowed proportion of heterozygotes within segments and a heterozygosity threshold to control the point at which the scan-ahead routine stops execution. For this report, the former threshold was set to 1% and the latter to 2%, which were based on analysis of the proportion of heterozygotes in different bin sizes across known autozygous segments.

### Comparison of hzAnalyzer and PLINK ROH output

We compared the output of our hzAnalyzer ROH detection method with output from PLINK v.1.0.7. We downloaded the PLINK format '.bed' files (hapmap_rel23a.zip) from their website [70], used the list of consensus rsids from our dataset for extracting loci, and detected ROH using PLINK's default settings. The default settings return ROH >1 Mb long, so for comparison, we extracted segments from our dataset using that length threshold. We used hzAnalyzer's get.generic.intersection function to intersect the PLINK (>1 Mb) set with our complete segments dataset filtered for segments with more than 50 SNPs. We then intersected our hzAnalyzer (>1 Mb) set with the PLINK (>1 Mb) set for the reverse comparison.

### Homozygosity probability score

To filter out spurious segments that were more likely due to chance, we calculated the homozygosity probability score (HPS) for each detected segment. We introduced HPS in a previous report [5], but modified it for this report's purposes to facilitate comparison between populations at local positions across the genome. We previously calculated HPS as the product of a segment's constituent loci's observed homozygosity frequencies (Freq_HOM_) from within the population to which a respective segment's sample belonged. However, that version of HPS is strongly influenced by allele frequencies in regions of the genome that have experienced population-specific fixation. Since we were interested in detecting and analyzing such fixed regions, we decided to use Freq_HOM _values calculated across the four populations' samples to calculate a population external version of the HPS statistic. We denote the modified version as HPS_ex _and the previous version as HPS_in_.

### Minimum inclusive segment length

To extract sets of higher confidence segments, we calculated what we here term a minimum inclusive segment length (MISL), which we had previously introduced [5] to more accurately compare sample populations on a genome-wide basis. Here we calculated MISL specific to each chromosome (MISL_chr_) by finding the longest segment in each individual for each chromosome and then choosing for each chromosome the lowest observed value. The genome-wide MISL (MISL_gw_) was simply the smallest value among the MISL_chr_. The MISL_chr _values for this current dataset are shown in Table S1 in Additional file 2. For analysis of chromosomes 7, 8, and X, we calculated the MISL_chr7,8,X _specific to this group of chromosomes from the lowest MISL_chr _value among them.

### Chromosome-specific coverage by homozygous segments

To calculate chromosome-specific coverage, we selected homozygous segments with SNP density >0.2 SNP/kb (1 SNP every 5 kb) and HPS_ex _≤0.01 and then calculated the cumulative sum of segment length across each chromosome for each individual, interpolated values for set length cutoffs, and then used the median values across each population sample at those cutoffs for plotting. We then calculated chromosomal coverage as the proportion of mappable chromosome length by dividing those median values by the mappable chromosome length, which we defined for each chromosome as Position_last SNP _- Position_first SNP _- Sum_length of gaps >500 kb_. An individual's data were excluded if the total SNP count contained in segments intersecting chromosomal abnormalities or CNVs (as defined above) was greater than 10% of a chromosome's total SNP count. Homozygous segments intersecting chromosomal abnormalities for individual samples that fell below this threshold were individually excluded from analysis.

### Intersecting segment length matrix

hzAnalyzer's segment detection process also outputs two matrices that we have termed intersecting segment length matrices (ISLMs). The ISLM matrix rows and columns correspond to individual samples and chromosomal SNP positions, respectively. Each ISLM cell contains the length of any segment in an individual that intersects a particular SNP position. The two matrices, ISLM_bp _and ISLM_cm_, are provided in base pair or centimorgan units, respectively, with centimorgan units calculated using chromosomal arm-specific average recombination rates based on the Rutger's second generation genetic map [71], which we downloaded from the project's website [72]. We additionally abbreviate each column in an ISLM as an ISLV. After segment detection and ISLM creation, we create copies that are masked (values replaced with NA = 'missing') for intersecting chromosomal abnormalities and CNVs.

#### Identifying putative autozygous segments

Since autozygous segments should be much longer than common homozygous segments within the same genomic coordinates, any measures that attempt to summarize the distribution of common homozygosity may be skewed by their presence. To ameliorate such effects, we developed a multi-locus method to quantify the extremeness of a segment's length compared to other samples' segments in the same coordinates. ISLVs underlying a particular target segment were extracted, and redundant ISLVs combined into a single representative ISLV. For each vector, the median and median absolute deviation were calculated across the non-zero ISLV values (ISLV_nz_), and a robust MAD score calculated as:

The median of a segment's ISLVs' MAD scores was assigned as that segment's MAD score. Putative autozygous segments for masking purposes were defined as those with segment MAD scores >10, while for autozygosity analysis they were defined as those with MAD scores >10 and founder haplotype frequency equal to zero.

#### Founder haplotype frequency estimation

To estimate the frequency of founder haplotypes underlying each homozygous segment, we first created a consensus phased haplotype dataset for UCSC hg18 genome build coordinates using the release 22 (2007-08_rel22) phased haplotypes for autosomes and the release 21 (2006-07 phase II files) phased haplotypes for chromosome X from the HapMap website [73]; the release 21 data were converted to hg18 coordinates using liftOver [74,75] with the hg17Tohg18.over.chain.files. We determined hg18 phased haplotypes for chromosome X for CEU and YRI trios using functions that we built into hzAnalyzer to perform deterministic phasing (see hzAnalyzer documentation). We used R's dist function to calculate the manhattan distance between each segment's founder haplotypes and the other phased haplotypes in the population sample and estimated the frequency of a segment's founder haplotypes as the proportion that were nearly matching (≤1% dissimilarity or ≤1 SNP difference, whichever was greatest).

### Calculating a measure of variation in the local extent of contiguous homozygosity

#### Masking segments and ISLM for putative autozygous segments

To reduce the effects of extremely long segments on the homozygous extent measures that we describe in this section, we extracted putative autozygous segments (segment length MAD score >10) and masked the corresponding samples and coordinates in the ISLM_bp _and ISLM_cm_. In contrast to how we masked chromosomal abnormalities and CNVs (in which corresponding cells were set to missing), we adjusted an extreme segment's value in a particular ISLV to that of the highest non-autozygous length in that vector.

#### Percentile-extent matrix

For each sample population and chromosome, we calculated the percentile values across each ISLV_bp _or ISLV_cm _using R's quantile function evaluated for probabilities 0 to 1 in 0.01 increments. In this report, we refer to the combined matrix of loci (rows) versus percentiles (columns) as the percentile-extent matrix (PE_mat_).

#### ext_AUC_: a variable derived by integrating homozygous extent

We reversed the sign of each ISLV's masked values in ISLM_cm _and then split each vector based on population sample. Each population's subvector was passed to R's ecdf function to calculate an ECDF object for each population's values within the ISLV. The sign reversal at the beginning of this process was meant to orient the values such that the less frequent longer segments represented the starting point for calculating the ECDF. We then integrated (using R's integrate function) the area under the curve of each ECDF. The lower and upper boundaries of the interval for integration were determined from the reversed sign ISLV values before splitting; the lower boundary was the lowest value after reversing sign (longest masked extent value) and the upper boundary was the closest value below zero. We used the closest value to zero as opposed to zero itself, since the area between the two had no measured values and appeared to add noise to the final ext_AUC _values. This process is diagrammed in Figure 3a-c.

#### Peak detection for delineation of ext_AUC _value extrema

To delineate the extremes of local variation in ext_AUC _values, we divided each population's values by determining local peaks and valleys in the data. To determine inflection points in the values, we first smoothed the data using R's smooth.spline function and calculated points at which the first derivative of the smooth spline function changed sign. The number of knots used by the smooth.spline function was allowed to vary as 3% of the loci that required smoothing. This parameter value allowed definition of peaks at an optimal resolution while reducing overfitting of the data. We then merged together neighboring peaks that possessed similar peak characteristics using an iterative process whereby two peaks were merged if the peak heights and intervening valley differed by less than 10%. We refer to the sets of peaks before and after merging as the complete peaks set and merged peaks set, respectively. Reference to processing 'peaks' in the next section or in the main text refers to the merged peaks set.

#### Definition of outlier peaks and peak regions

To extract peaks that appeared extreme for each of the populations, we determined outliers at the high end of the distribution of ext_AUC _peak heights using standard R boxplot statistics (>upper whisker; >Third quartile + 1.5 × Inter-quartile range). This process was performed for each population with outlier peaks for each chromosome calculated separately. We performed an additional round of peak merging to merge together any directly adjoining outlier peaks that lacked clear separation (Intervening valley >0.5 × Peaks' heights) into a set of outlier peak regions.

#### Comparison of the extent and frequency of homozygous segments with haplotypes underlying ext_AUC _peaks

For each peak, outlier peak, and outlier peak region, we analyzed the correspondence between the percentile distribution of segment length summarized within PE_mat _and the frequency of haplotypes within each population sample. Figure 5 diagrams the analytical procedure. For each of a peaks' ISLVs, we transformed the PE_mat _percentile values into their complementary quantile values such that:

We then defined a peak's optimal parameters for *Extent *and *Pr*(*X *>*Extent*) as those that maximized the value of *Extent *× *Pr*(*X *>*Extent*), as illustrated by the red rectangle in the right-center panel of Figure 5. We label those optimized values of *Pr*(*X *>*Extent*) and *Extent *as *p_max _*and *Extent_min_*. If a single haplotype is responsible for a peak's observed contiguous homozygosity with length greater than *Extent_min_*, then its frequency should be close to the square-root of *p_max_*, which we use as the expected haplotype frequency (*Freq_hap-exp_*). As a haplotype frequency estimate for a particular peak (*Freq_hap-max_*), we used the maximum of the estimated haplotype frequencies among segments with length greater than *Extent_min_*.

#### Intersection of peaks with canonical coding regions

To determine genes underlying each peak or peak region, we intersected their coordinates with a set of genes and coding regions. We downloaded the hg18 11-May-2009 versions of the kgXref, kgTxInfo, and knownCanonical tables [74] from the sequence and annotation page of the UCSC Genome Browser website [76]. We joined these tables together using the kgID, name, or transcript fields, respectively, and filtered the resulting table to include only data with the categories of 'coding' or 'antibodyParts'. Entries with the same geneSymbol that had overlapping coordinates were merged together.

#### ext_AUC _rank value calculation

In order to quantify how peak delineated ext_AUC _values in one population compared with ext_AUC _values in the others, we transformed ext_AUC _values into a rank value using the empirical cumulative distribution function (with each population and chromosome performed separately). Using R's ecdf function, this simply converted each of the ext_AUC _values into its cumulative probability; for an ext_AUC _value *x*, F(*x*) = P(X ≤ *x*). We then assigned each peak the maximum rank value across the loci between its lower and upper valleys and also annotated each peak with the highest rank value for each of the other populations' ext_AUC _values within the peak's coordinates.

#### Definition of genomic regions containing areas of contiguous loci at or near fixation

To detect candidate fixed areas, we examined PE_mat _for runs of contiguous loci that had non-zero extent values in the 0th percentile (RCL_0_). As such, these should represent areas of the genome that possess overlapping homozygous segments in all of a population's individuals. To remove the shortest runs with small numbers of loci, we defined candidate fixed areas as RCL_0 _that had centimorgan extent values and SNP counts greater than the first quartile within each population. We intersected each candidate fixed area with the canonical coding region gene set described above as well as with the set of outlier peak regions. We merged the CEU, CHB, and JPT candidate region tables and resolved overlaps between the regions to create a set of combined candidate fixed regions. Candidate fixed areas, regions, and the combined regions were intersected with data from several earlier reports that had investigated selective sweeps or fixation using HapMap data [5,27-30].

#### Calculation of Fst/θ combined across loci underlying outlier peaks

For each peak, we quantified differentiation between the peak's sample population and each of the other populations using a standard Fst measure. For this comparison, we used Weir and Hill's genotype frequency-based moment estimator of θ that includes terms to adjust for differences in population sample size [24,25].

We extracted peaks for either CHB or JPT that had maximum ext_AUC _values in the top 10% of both populations' ext_AUC _value distribution and that had Fst/θ values exceeding 0.0360 for autosomes and 0.0538 for chromosome X. For each of those peaks, we extracted high Fst/θ SNPs underlying each outlier peak and estimated the haplotype frequency as the average allele frequency across those highly differentiated loci (after orienting the allele frequencies so that the minor allele was considered the *A *allele).

#### Concordance of putative autozygosity with 1000 Genomes Project genotypes

The 1000 Genomes Project November release genotype data (file All.2of4intersection.20100804.genotypes.vcf.gz) [77] were downloaded and then processed using VCFtools [78]. Of 231 HapMap Phase 2 samples with autozygous segments, 140 were also present in 1000G, and 413 of 636 putative autozygous segments were available for comparison. Within each segment's coordinates, we calculated the proportion of heterozygous genotypes in each 1000G sample as well as a z-score and rank comparing the segment's sample heterozygosity with that in its corresponding sample population.

### Analysis of ext_AUC _peak height and 1000 Genomes Project recombination rate

We downloaded recombination rate/genetic map data (file 1000G_LC_Pilot_genetic_map_b36_genotypes_10_2010.tar.gz) for the 1000 Genomes Project pilot data [79] and created population-specific genetic maps using the 1000G population-specific recombination rate maps (CHB and JPT are combined). The available genetic map data only included autosomes, so we could not analyze chromosome X peaks in our dataset. We calculated the genetic map distance (centimorgans) and recombination rate across both the full-width as well as central half-width for each peak in the merged peak dataset. Peak central half-width was defined as the midpoint of the upper 25% of the peak's ext_AUC _values ± one-half of the peak's width. Recent reports showing that only a small fraction of the genome accounts for most recombination [5,21] suggest that the distribution of recombination rate values varies depending on the size of the region examined. To account for this, we transformed the peaks' recombination rate values into cumulative probabilities (that is, percentile ranks), with each peak width/half-width recombination rate matched with values averaged across bins of similar size (bin widths: 5 kb, every 10 kb from 10 kb to 100 kb, every 100 kb from 200 kb to 500 kb, 750 kb, 1,000 kb, 5,000 kb, 10,000 kb) for the same chromosome and population.

#### Extending hzAnalyzer to case-control association analysis

To compare the distribution of segment length for a particular genomic position between two population samples, we calculated a version of the two-sample CVM test statistic ω^2 ^[35-37], the integral of the squared differences between the two samples' ECDFs evaluated at each of the *x *values across the combined set of samples. For the example in this report, we used the JPT and CHB data from the ISLM_cm _for chromosome 20, split the data for the two samples, and created two function objects using R's ecdf function at each SNP position. The functions were then evaluated using the unique length values across the combined set of JPT and CHB values at a particular position. To determine the significance of a particular position's ω^2 ^value, we performed a permutation test [80] by randomly rearranging the two samples' labels and recalculating ω^2 ^for enough replications that an accurate approximate achieved significance level could be calculated.

### Data visualization

hzAnalyzer includes functions to assist in constructing several types of complex figures for comparing populations as well as different regions of the genome. Phased haplotype plots used the phased haplotype data described under the section 'Founder haplotype frequency estimation'. All samples' haplotype data for a plotted region was ordered using R's agnes function (cluster package), which performs agglomerative hierarchical clustering, and each group's haplotypes plotted separately. Documentation on the hzAnalyzer website [21] can assist in producing some of the figures described in this report.

## Abbreviations

1000G: 1000 Genomes Project dataset; AHA: agglomerative haplotype analysis; bp: base pairs; CEU: Utah residents with ancestry from northern and western Europe; CHB: Han Chinese in Beijing, China; Chr: chromosome; cM: centimorgan; CNV: copy-number variation; CVM: Cramer-von Mises statistic; ECDF: empirical cumulative distribution function; ext_AUC_: extent-area under the curve; HPS: homozygosity probability score; HPS_ex_: population external homozygosity probability score; ISLM: intersecting segment length matrix; ISLV: intersecting segment length vector; JPT: Japanese in Tokyo, Japan; LD: linkage disequilibrium; MAD: median absolute deviation; MAF: minor allele frequency; MISL: minimum inclusive segment length; PE_mat_: percentile-extent matrix; RCL_0_: run of consecutive loci in the 0th percentile of PE_mat_; ROH: runs of homozygosity; SD: standard deviation; SNP: single nucleotide polymorphism; YRI: Yoruba from Ibadan, Nigeria.

## Authors' contributions

TAJ: research plan development, programming, data analysis, figures, and manuscript preparation. YoN: advisor on genomic analysis, data analysis, and programming and manuscript preparation. HT: advisor on bioinformatics development. YN: advisor on human genetics and research plan development. TT: principal advisor for research plan development, data analysis, programming, figures and manuscript preparation.

## Supplementary Material

Additional file 1 **Figure S1**. Genome-wide plot of greater confidence homozygous segments. The chromosomal positions of homozygous segments with length ≥MISL_chr _were plotted for all 269 samples (arrayed along the y-axis). The relative SNP density compared to the maximum for that chromosome is plotted at the top of each panel. Homozygous segments were color-coded depending on different status types. Red lines, homozygous segments ≥MISL_chr_; green lines, putative autozygous segments (MAD score >10); yellow lines, ≤0.2 SNP/kb; blue line, high missingness (no-call rate >0.05); orange lines, sample level CNVs. Click here for file

Additional file 2 **Supplementary Tables 1 to 8**. Table S1: minimum inclusive segment lengths (bp) calculated for each chromosome for each population and across all populations. Table S2a-d: putative autozygous segment coordinates for YRI, CEU, CHB, and JPT, respectively. Segment coordinates and parameters are listed for segments with a segment length median MAD score greater than 10. Table S3a-d: tables of all detected outlier peaks and derived peak statistics for YRI, CEU, CHB, and JPT, respectively. Outlier peaks by peak height (ext_AUC_) were determined separately for each population and chromosome and statistics extracted for different peak features. Table S4a-d: tables of all detected outlier peak regions and summary of underlying peak statistics for YRI, CEU, CHB, and JPT, respectively. Outlier peaks that were directly adjacent to each other and were not well separated (Valleys > 0.5 × Peak height) were merged to define peak regions, and statistics were summarized across the underlying peaks. Table S5a,b: peaks with high-ranking ext_AUC _and high Fst/θ between CHB and JPT represent extended haplotypes with high frequency differences. Peaks were extracted that had both high-ranking ext_AUC _values and extreme Fst/θ values (autosomes >0.0360 or chromosome X >0.0538) in both/between CHB and JPT. For each peak, approximate haplotype frequencies were estimated using the allele frequencies of the differentiated loci. Table S6a-d: candidate fixed areas: genomic areas of contiguous loci with evidence for fixation. RCL_0 _were selected using thresholds set as the first quartiles of RCL_0 _SNP counts and RCL_0 _centimorgan extent values across all populations. Selected RCL_0 _were then intersected with a dataset of genes and canonical coding regions as well as data from previous reports by Kimura *et al. *[27], Sabeti and colleagues [5,28], Tang *et al. *[29], and O'Reilly *et al. *[30]. Table S7a-c: outlier peak regions intersecting candidate fixed areas in CEU, CHB, or JPT. Outlier peak regions were intersected with the set of candidate fixed areas presented in Table S6a-d. These peak regions were then intersected with a dataset of genes and canonical coding regions as well as data from previous reports by Kimura *et al. *[27], Sabeti and colleagues [5,28], Tang *et al. *[29], and O'Reilly *et al. *[30]. Table S8: combined candidate fixed regions. Coordinates of candidate fixed peak regions from Table S7a-c that overlapped were merged into a set of combined candidate fixed regions. These regions were annotated with genes that directly intersected any candidate fixed areas as well as the number of overlapping detected regions in each of the previous reports by Kimura *et al. *[27], Sabeti and colleagues [5,28], Tang *et al. *[29], and O'Reilly *et al. *[30]. Click here for file

Additional file 3 **Figure S2**. Chromosome profiles of percent coverage by putative autozygous segments. Putative autozygous segments were defined as homozygous segments with length-based MAD score >10 and the percent coverage of each chromosome calculated for each sample. Pages are labelled with population name and gender at the top. Sample profiles on each page are ordered by increasing genome-wide coverage. The y-axis maximum limit is set to 5.0%. For coverage values ≥5.0%, the plotted points extend off the top of the plot and the percent value is printed underneath the peak. Click here for file

Additional file 4 **Figure S3**. The local centimorgan extent of homozygosity across the genome at the 75th percentile. Homozygous extent values are plotted in centimorgans for the 75th percentile for each sample population. Physical distance (base pairs) was converted into genetic distance (centimorgans) using chromosome arm averaged recombination rates. To reduce the large number of plotted datapoints, we smoothed these values using smooth splines and then down-sampled the predicted values. The y-axis is set dynamically to the highest observed peak for a particular chromosome. Click here for file

Additional file 5 **Figure S4a**. Genome-wide visualization of PE_mat _and ext_AUC _values for YRI. PE_mat _(cM) matrix values were scaled based on a maximum value of 2 cM, converted into grayscale levels, and plotted by chromosome. Cells with values ≥2 cM were set to black to compress and standardize the dynamic range. Red line: smoothed ext_AUC _values were down-sampled. The scale for ext_AUC _values is set separately to the maximum value observed across all autosomes or on chromosome X. Chromosomes are ordered by chromosomal base-pair length. Click here for file

Additional file 6 **Figure S4b**. Genome-wide visualization of PE_mat _and ext_AUC _values for CEU. PE_mat _(cM) matrix values were scaled based on a maximum value of 2 cM, converted into grayscale levels, and plotted by chromosome. Cells with values ≥2 cM were set to black to compress and standardize the dynamic range. Red line: smoothed ext_AUC _values were down-sampled. The scale for ext_AUC _values is set separately to the maximum value observed across all autosomes or on chromosome X. Chromosomes are ordered by chromosomal base pair length. Click here for file

Additional file 7 **Figure S4c**. Genome-wide visualization of PE_mat _and ext_AUC _values for CHB. PE_mat _(cM) matrix values were scaled based on a maximum value of 2 cM, converted into grayscale levels, and plotted by chromosome. Cells with values ≥2 cM were set to black to compress and standardize the dynamic range. Red line: smoothed ext_AUC _values were down-sampled. The scale for ext_AUC _values is set separately to the maximum value observed across all autosomes or on chromosome X. Chromosomes are ordered by chromosomal base pair length. Click here for file

Additional file 8 **Figure S4d**. Genome-wide visualization of PE_mat _and ext_AUC _values for JPT. PE_mat _(cM) matrix values were scaled based on a maximum value of 2 cM, converted into grayscale levels, and plotted by chromosome. Cells with values ≥2 cM were set to black to compress and standardize the dynamic range. Red line: smoothed ext_AUC _values were down-sampled. The scale for ext_AUC _values is set separately to the maximum value observed across all autosomes or on chromosome X. Chromosomes are ordered by chromosomal base pair length. Click here for file

Additional file 9 **Figure S5**. Comparison of the extent and frequency of homozygous segments with haplotypes underlying ext_AUC _peaks. Analysis of the consistency of the homozygous extent distribution and length and frequency of haplotypes for ext_AUC _peaks in YRI, CEU, CHB, and JPT. Minimum segment length (*Extent_min_*), expected haplotype frequency (*Freq_hap-exp_*), and maximum haplotype frequency (*Freq_hap-max_*) were calculated as diagrammed in Figure 5 for peaks dichotomized into non-outlier and outlier peaks. Data points were colored using a two-dimesnional density estimate using R's function densCols with nbin = 1,024. Click here for file

Additional file 10 **Figure S6**. Majority of outlier peaks intersect with similarly high-ranking ext_AUC _values in other populations. Each population's chromosome's ext_AUC _values were used as input to R's ecdf function to substitute a rank value for each locus's ext_AUC _value. For each outlier peak, locus positions were extracted, the maximum observed ext_AUC _rank value for those positions in each of the other populations determined, and the distribution of those rank values summarized using boxplot statistics. Outlier points are randomly jittered from left to right to reduce overlap. Click here for file

Additional file 11 **Figure S7**. Phased haplotype plots for two regions with both high-ranking ext_AUC _and high Fst/θ values between populations. Phased haplotypes were plotted for two example regions exhibiting high cross-population ext_AUC _values as well as high population differentiation: page 1, Chr X:62.7-67Mb; page 2, Chr 14:65.4-67 Mb. Click here for file

Additional file 12 **Figure S8**. High-ranking ext_AUC _values and high Fst/θ between East Asian population samples identify peaks intersecting multi-locus haplotypes with high frequency differences. Peaks were selected that had high-ranking ext_AUC _values in the two groups (≥90th percentile) as well as extreme Fst/θ values (Chr X Fst/θ >0.0538, autosome Fst/θ >0.0360). The peaks were sorted in decreasing order using the proportion of loci with extreme Fst/θ values. The top five peaks for CHB and JPT are shown. Click here for file

Additional file 13 **Figure S9**. Phased haplotype plots in combined fixation candidate regions. Phased haplotypes were plotted for combined fixation candidate regions that are mentioned in the Discussion. These include three regions on chromosome X that were not reported in the other examined datasets: page 1, Chr X:104.2-105.5 Mb; page 2, Chr X:113.7-114.4 Mb; page 3, Chr X:126.1-127.7 Mb, and one region in JPT intersecting the *EXOC6B *gene; page 4, Chr 2:71.9-73.1 Mb. Click here for file
