# Supplementary figures and images for: hzAnalyzer: detection, quantification, and visualization of contiguous homozygosity in high-density genotyping datasets
Source: Genome Biol. 2011 Mar 11;12(3):R21. doi: 10.1186/gb-2011-12-3-r21 (PMC3129671; doi:10.1186/gb-2011-12-3-r21)

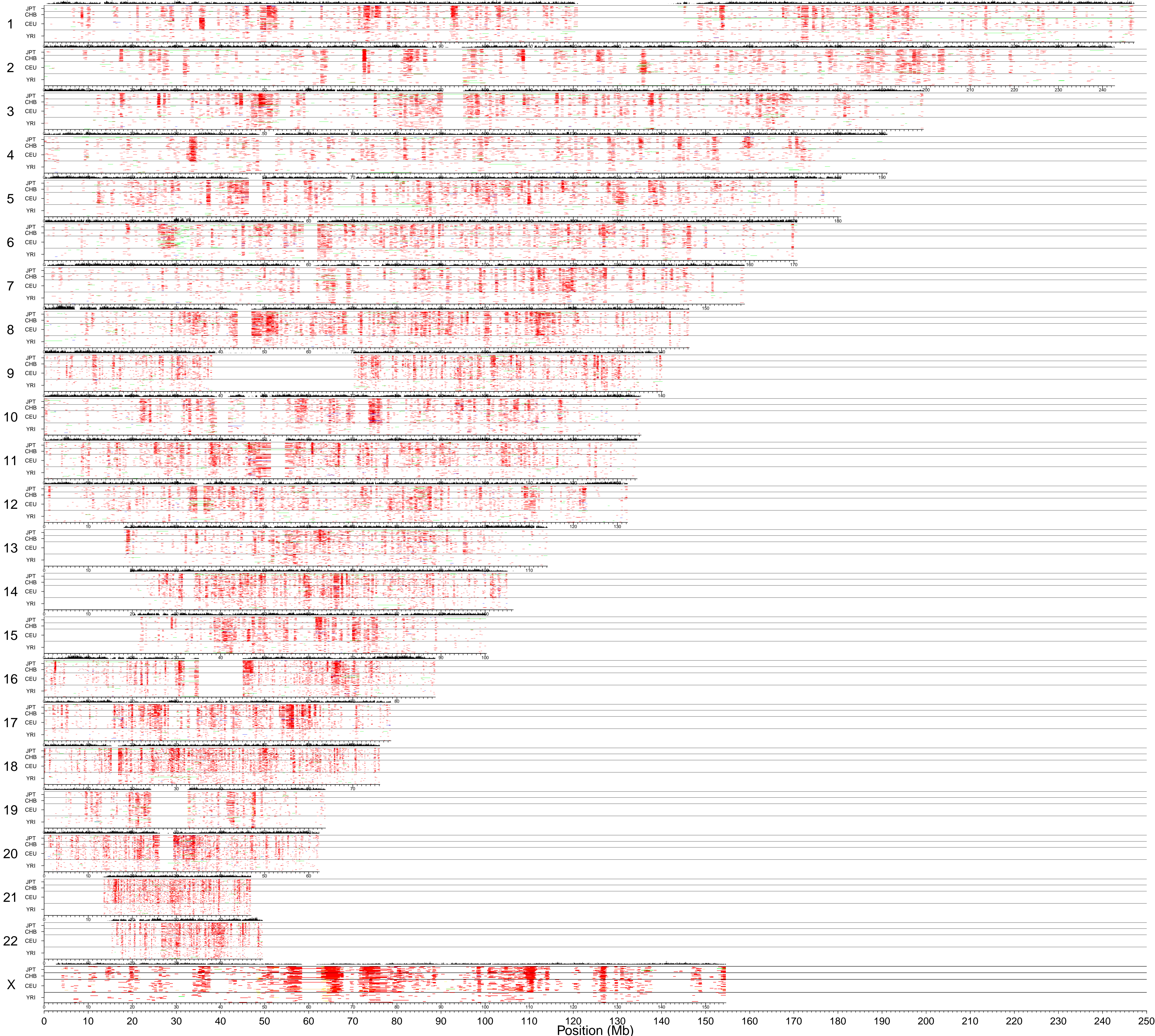

Supplement: Additional file 1 — Figure S1. Genome-wide plot of greater confidence homozygous segments. The chromosomal positions of homozygous segments with length ≥MISLchr were plotted for all 269 samples (arrayed along the y-axis). The relative SNP density compared to the maximum for that chromosome is plotted at the top of each panel. Homozygous segments were color-coded depending on different status types. Red lines, homozygous segments ≥MISLchr; green lines, putative autozygous segments (MAD score >10); yellow lines, ≤0.2 SNP/kb; blue line, high missingness (no-call rate >0.05); orange lines, sample level CNVs. [file gb-2011-12-3-r21-S1.PDF]

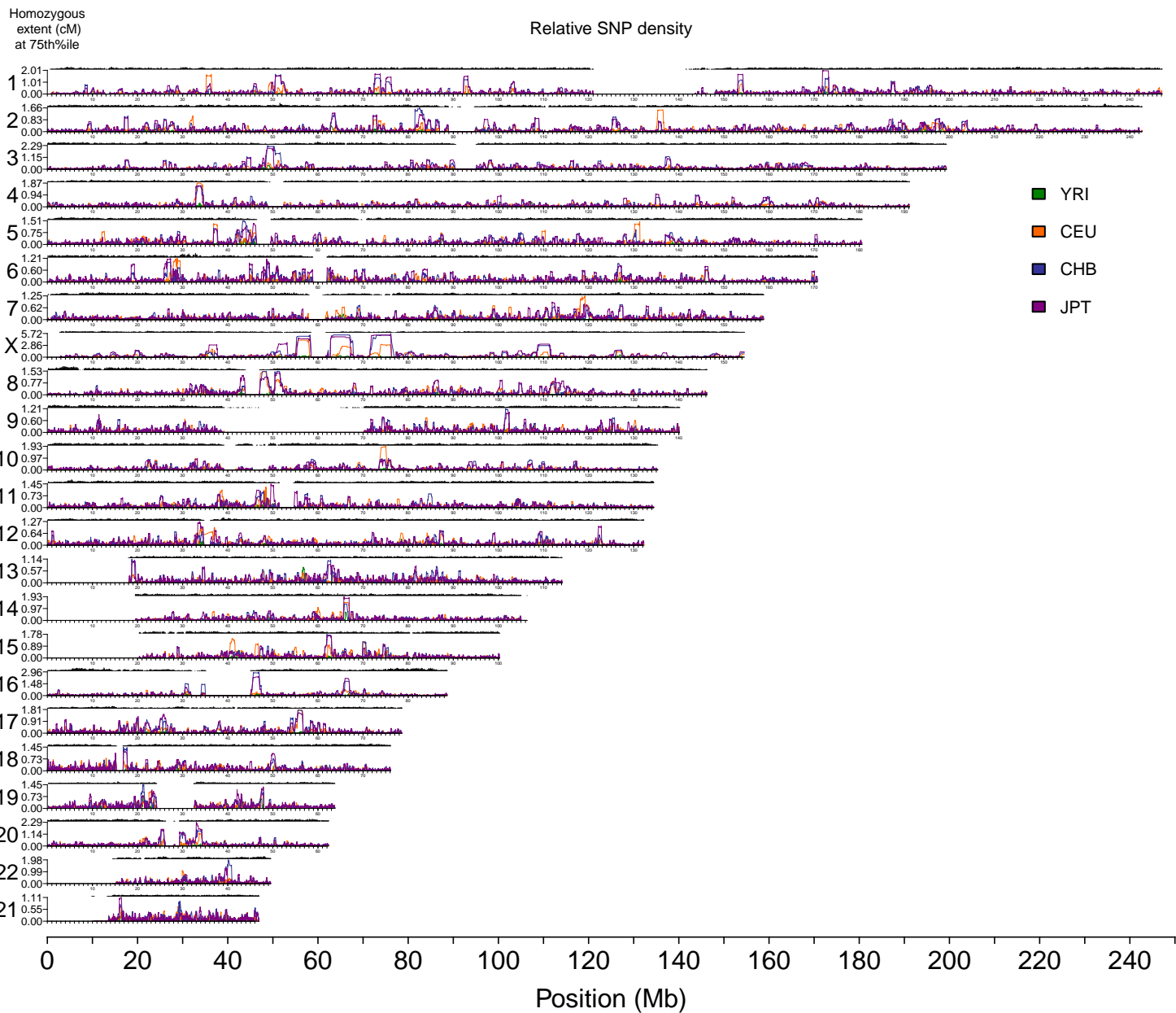

Supplement: Additional file 4 — Figure S3. The local centimorgan extent of homozygosity across the genome at the 75th percentile. Homozygous extent values are plotted in centimorgans for the 75th percentile for each sample population. Physical distance (base pairs) was converted into genetic distance (centimorgans) using chromosome arm averaged recombination rates. To reduce the large number of plotted datapoints, we smoothed these values using smooth splines and then down-sampled the predicted values. The y-axis is set dynamically to the highest observed peak for a particular chromosome. [file gb-2011-12-3-r21-S4.PDF]

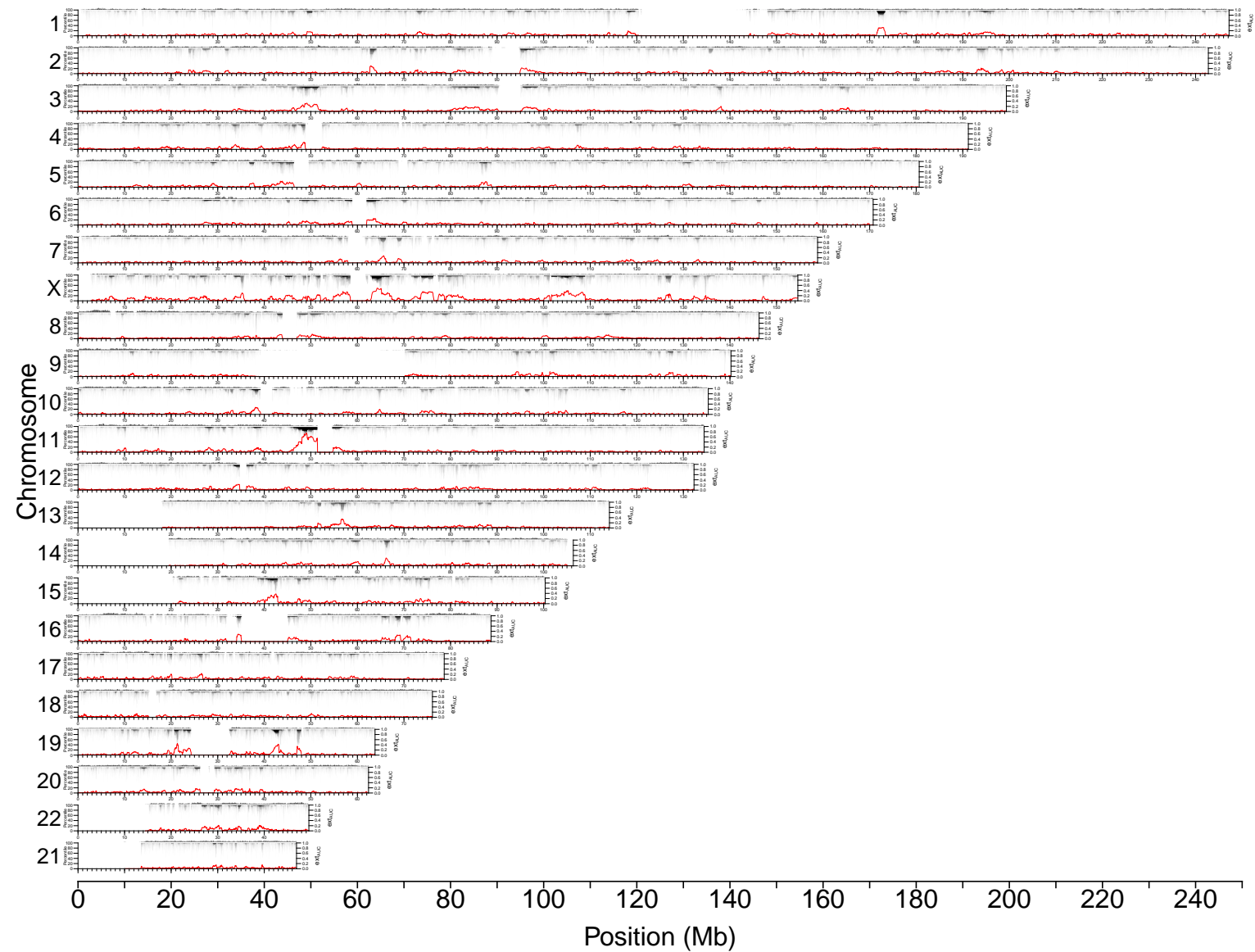

Supplement: Additional file 5 — Figure S4a. Genome-wide visualization of PEmat and extAUC values for YRI. PEmat (cM) matrix values were scaled based on a maximum value of 2 cM, converted into grayscale levels, and plotted by chromosome. Cells with values ≥2 cM were set to black to compress and standardize the dynamic range. Red line: smoothed extAUC values were down-sampled. The scale for extAUC values is set separately to the maximum value observed across all autosomes or on chromosome X. Chromosomes are ordered by chromosomal base-pair length. [file gb-2011-12-3-r21-S5.PDF]

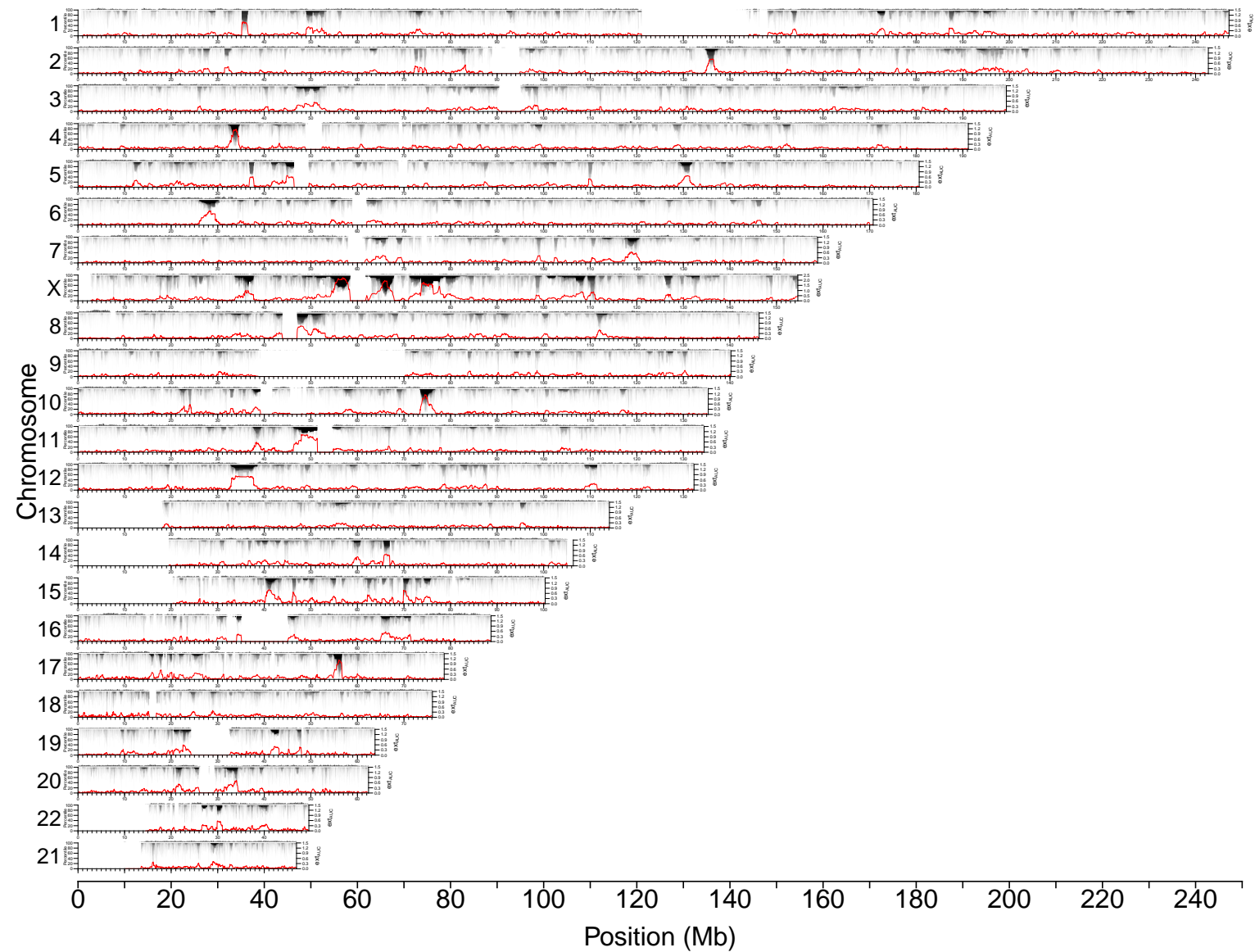

Supplement: Additional file 6 — Figure S4b. Genome-wide visualization of PEmat and extAUC values for CEU. PEmat (cM) matrix values were scaled based on a maximum value of 2 cM, converted into grayscale levels, and plotted by chromosome. Cells with values ≥2 cM were set to black to compress and standardize the dynamic range. Red line: smoothed extAUC values were down-sampled. The scale for extAUC values is set separately to the maximum value observed across all autosomes or on chromosome X. Chromosomes are ordered by chromosomal base pair length. [file gb-2011-12-3-r21-S6.PDF]

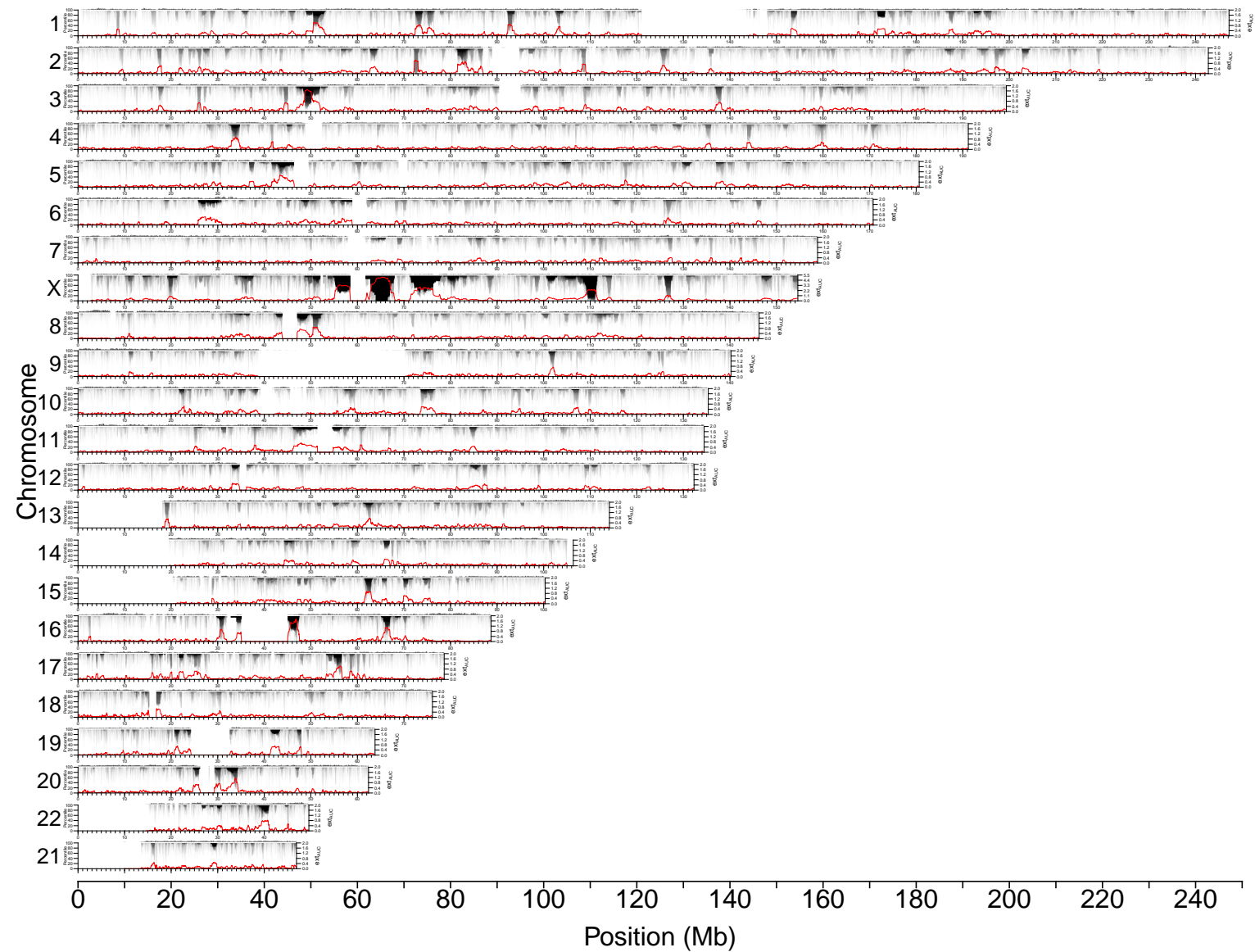

Supplement: Additional file 7 — Figure S4c. Genome-wide visualization of PEmat and extAUC values for CHB. PEmat (cM) matrix values were scaled based on a maximum value of 2 cM, converted into grayscale levels, and plotted by chromosome. Cells with values ≥2 cM were set to black to compress and standardize the dynamic range. Red line: smoothed extAUC values were down-sampled. The scale for extAUC values is set separately to the maximum value observed across all autosomes or on chromosome X. Chromosomes are ordered by chromosomal base pair length. [file gb-2011-12-3-r21-S7.PDF]

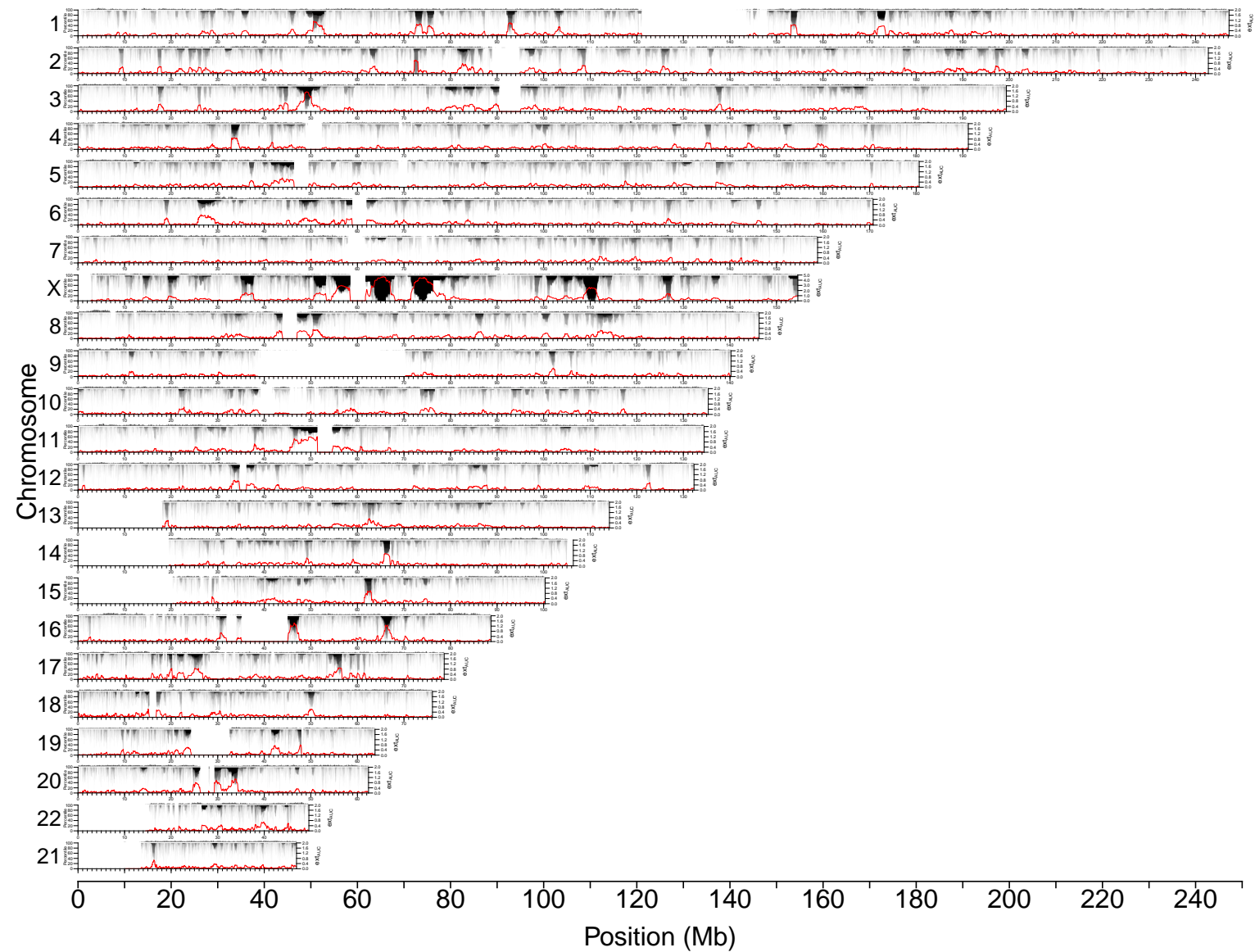

Supplement: Additional file 8 — Figure S4d. Genome-wide visualization of PEmat and extAUC values for JPT. PEmat (cM) matrix values were scaled based on a maximum value of 2 cM, converted into grayscale levels, and plotted by chromosome. Cells with values ≥2 cM were set to black to compress and standardize the dynamic range. Red line: smoothed extAUC values were down-sampled. The scale for extAUC values is set separately to the maximum value observed across all autosomes or on chromosome X. Chromosomes are ordered by chromosomal base pair length. [file gb-2011-12-3-r21-S8.PDF]

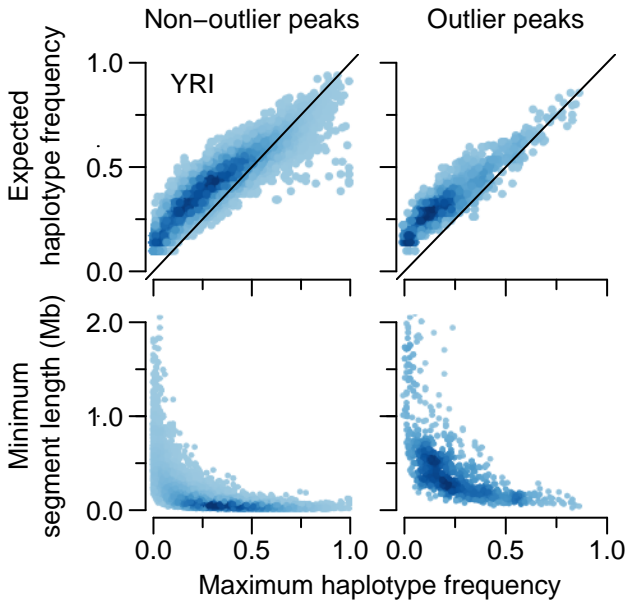

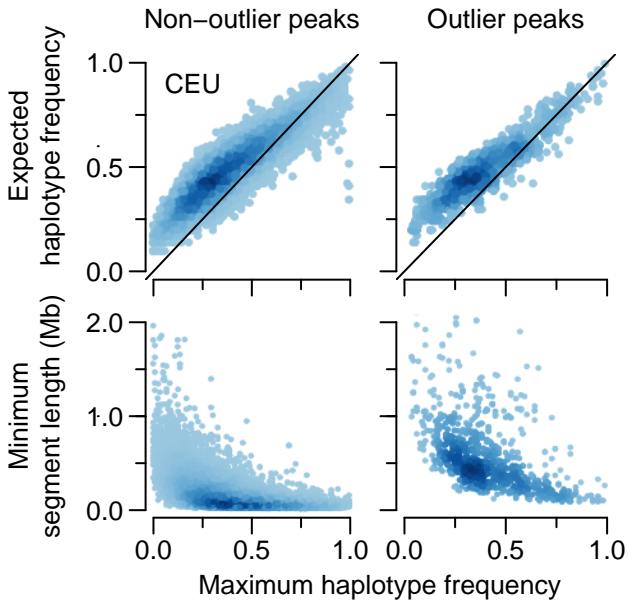

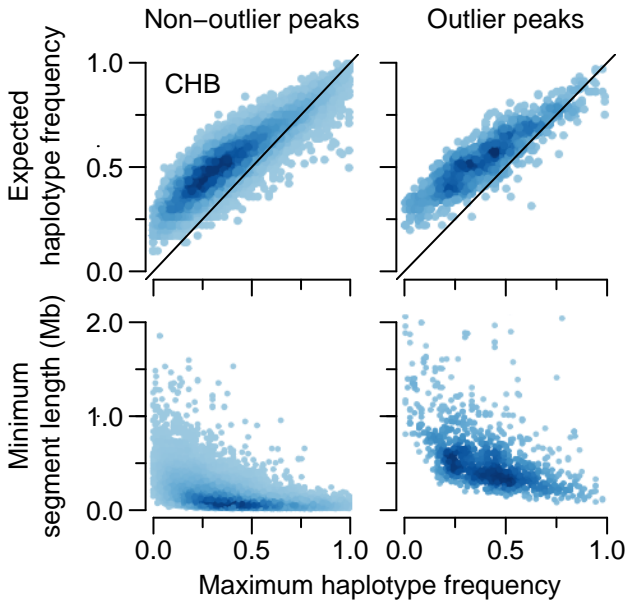

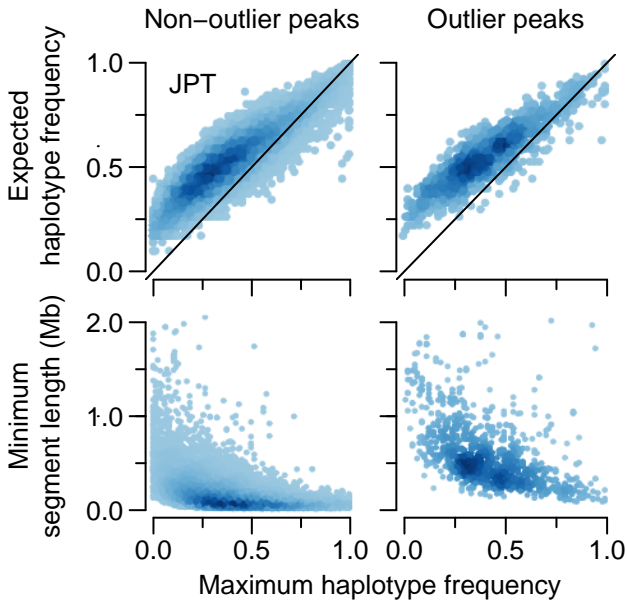

Supplement: Additional file 9 — Figure S5. Comparison of the extent and frequency of homozygous segments with haplotypes underlying extAUC peaks. Analysis of the consistency of the homozygous extent distribution and length and frequency of haplotypes for extAUC peaks in YRI, CEU, CHB, and JPT. Minimum segment length (Extentmin), expected haplotype frequency (Freqhap-exp), and maximum haplotype frequency (Freqhap-max) were calculated as diagrammed in Figure 5 for peaks dichotomized into non-outlier and outlier peaks. Data points were colored using a two-dimesnional density estimate using R's function densCols with nbin = 1,024. [file gb-2011-12-3-r21-S9.PDF]

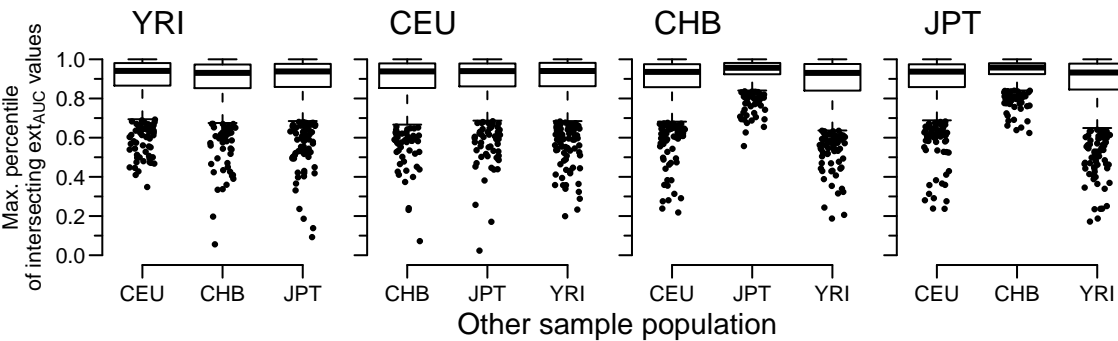

Supplement: Additional file 10 — Figure S6. Majority of outlier peaks intersect with similarly high-ranking extAUC values in other populations. Each population's chromosome's extAUC values were used as input to R's ecdf function to substitute a rank value for each locus's extAUC value. For each outlier peak, locus positions were extracted, the maximum observed extAUC rank value for those positions in each of the other populations determined, and the distribution of those rank values summarized using boxplot statistics. Outlier points are randomly jittered from left to right to reduce overlap. [file gb-2011-12-3-r21-S10.PDF]
