# Supplementary material for: hzAnalyzer: detection, quantification, and visualization of contiguous homozygosity in high-density genotyping datasets
Source: Genome Biol. 2011 Mar 11;12(3):R21. doi: 10.1186/gb-2011-12-3-r21 (PMC3129671; doi:10.1186/gb-2011-12-3-r21)
Supplement: Additional file 3 — Figure S2. Chromosome profiles of percent coverage by putative autozygous segments. Putative autozygous segments were defined as homozygous segments with length-based MAD score >10 and the percent coverage of each chromosome calculated for each sample. Pages are labelled with population name and gender at the top. Sample profiles on each page are ordered by increasing genome-wide coverage. The y-axis maximum limit is set to 5.0%. For coverage values ≥5.0%, the plotted points extend off the top of the plot and the percent value is printed underneath the peak. [file gb-2011-12-3-r21-S3.PDF]

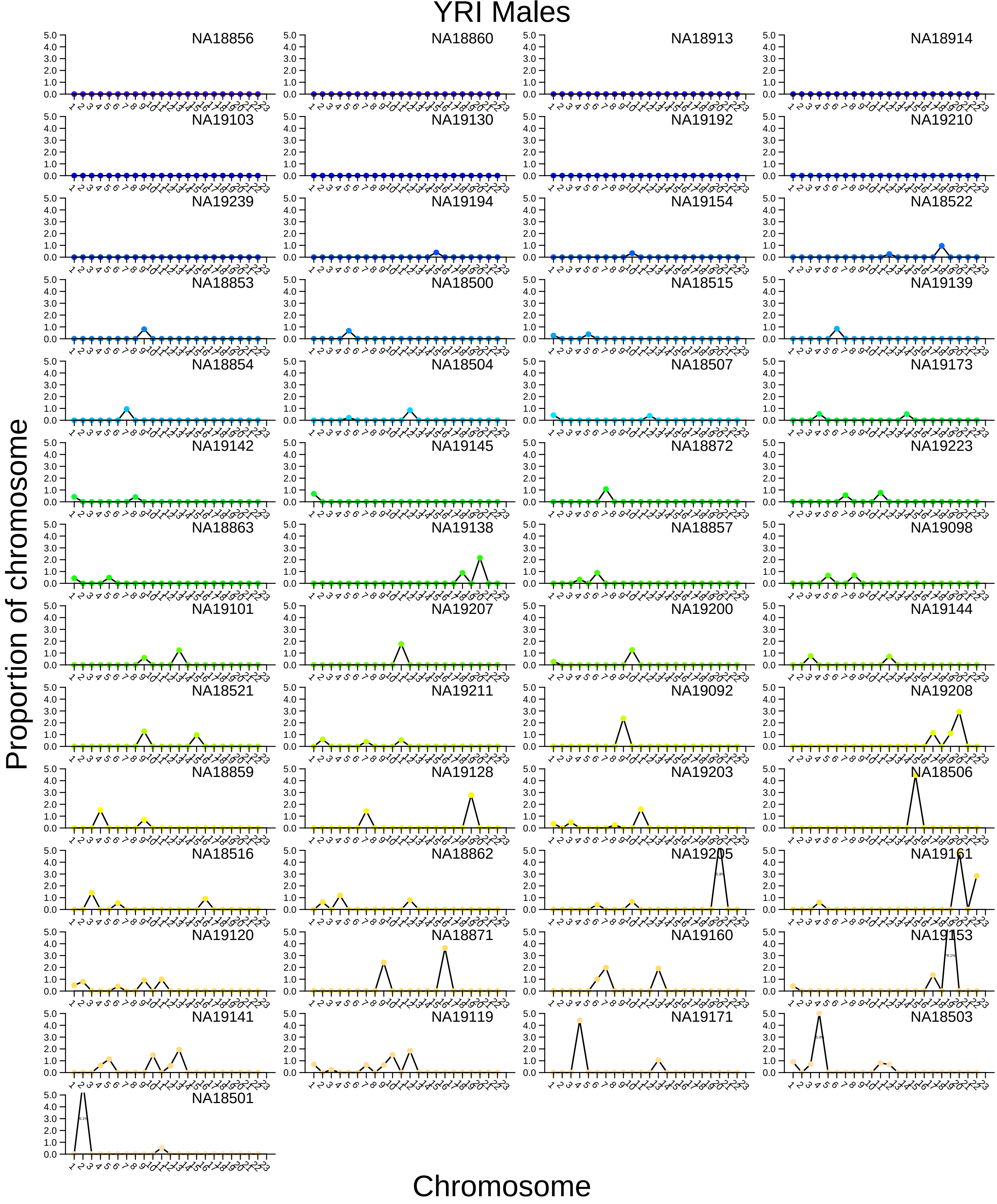
YRI Males
Proportion of chromosome
Chromosome
NA18856
NA18860
NA18913
NA18914
NA19103
NA19130
NA19192
NA19210
NA19239
NA19194
NA19154
NA18522
NA18853
NA18500
NA18515
NA19139
NA18854
NA18504
NA18507
NA19173
NA19142
NA19145
NA18872
NA19223
NA18863
NA19138
NA18857
NA19098
NA19101
NA19207
NA19200
NA19144
NA18521
NA19211
NA19092
NA19208
NA18859
NA19128
NA19203
NA18506
NA18516
NA18862
NA19205
NA19161
NA19120
NA18871
NA19160
NA19153
NA19141
NA19119
NA19171
NA18503
NA18501

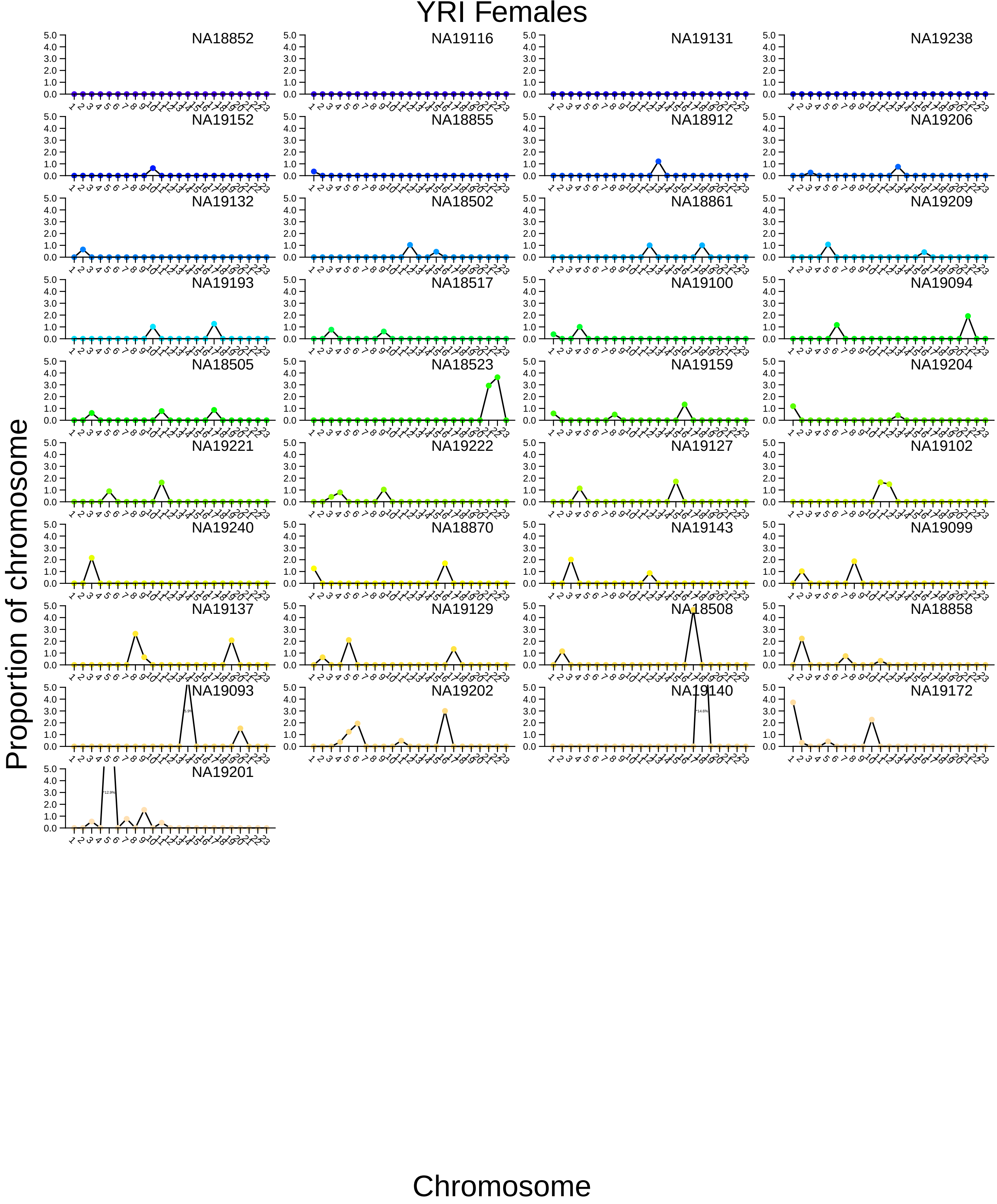

YRI Females
Proportion of chromosome
Chromosome
NA18852
NA19116
NA19131
NA19238
NA19152
NA18855
NA18912
NA19206
NA19132
NA18502
NA18861
NA19209
NA19193
NA18517
NA19100
NA19094
NA18505
NA18523
NA19159
NA19204
NA19221
NA19222
NA19127
NA19102
NA19240
NA18870
NA19143
NA19099
NA19137
NA19129
NA18508
NA18858
NA19093
NA19202
NA19140
NA19172
NA19201

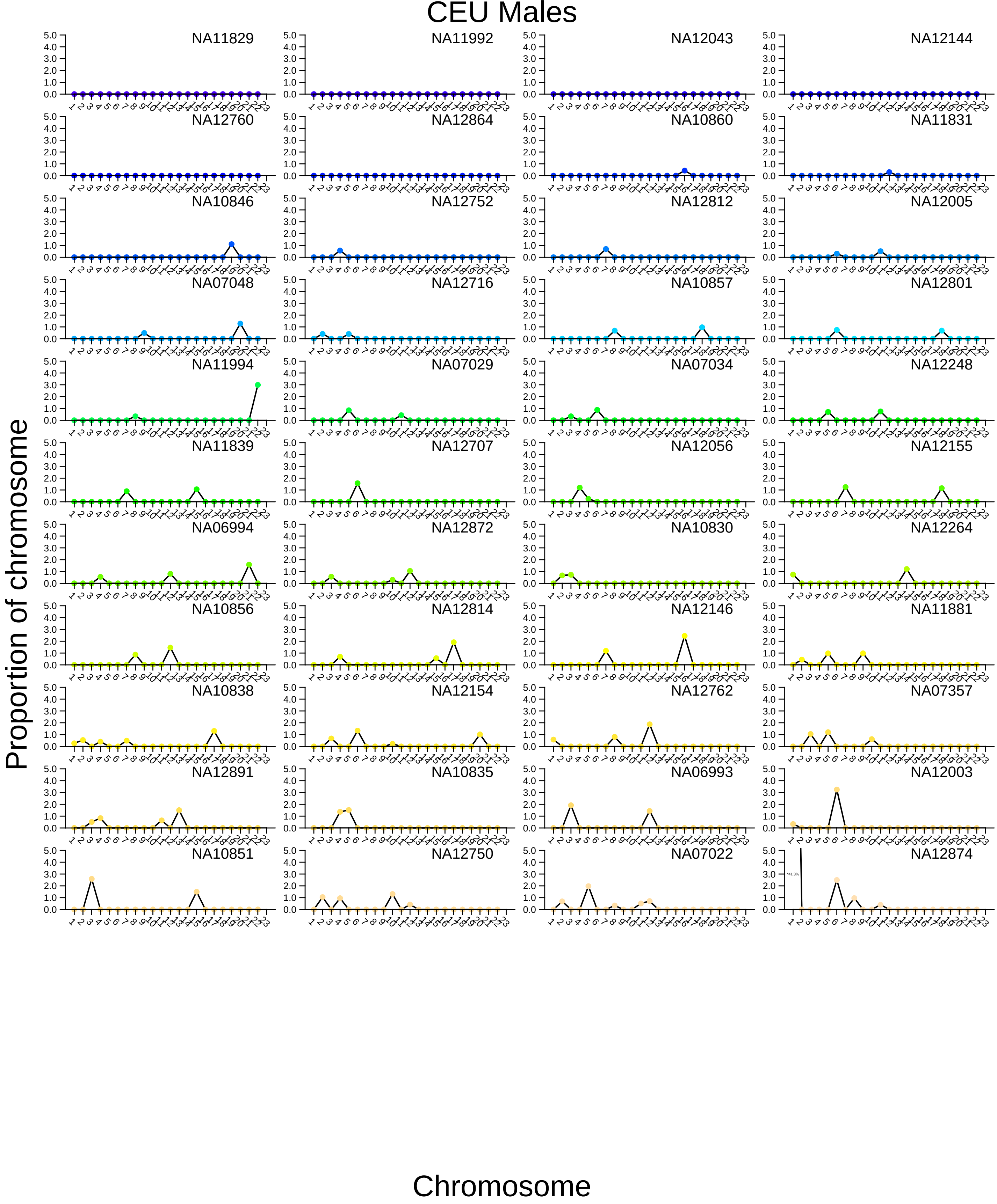
CEU Males
NA11829
NA11992
NA12043
NA12144
NA12760
NA12864
NA10860
NA11831
NA10846
NA12752
NA12812
NA12005
NA07048
NA12716
NA10857
NA12801
NA11994
NA07029
NA07034
NA12248
NA11839
NA12707
NA12056
NA12155
NA06994
NA12872
NA10830
NA12264
NA10856
NA12814
NA12146
NA11881
NA10838
NA12154
NA12762
NA07357
NA12891
NA10835
NA06993
NA12003
NA10851
NA12750
NA07022
NA12874
Proportion of chromosome
Chromosome

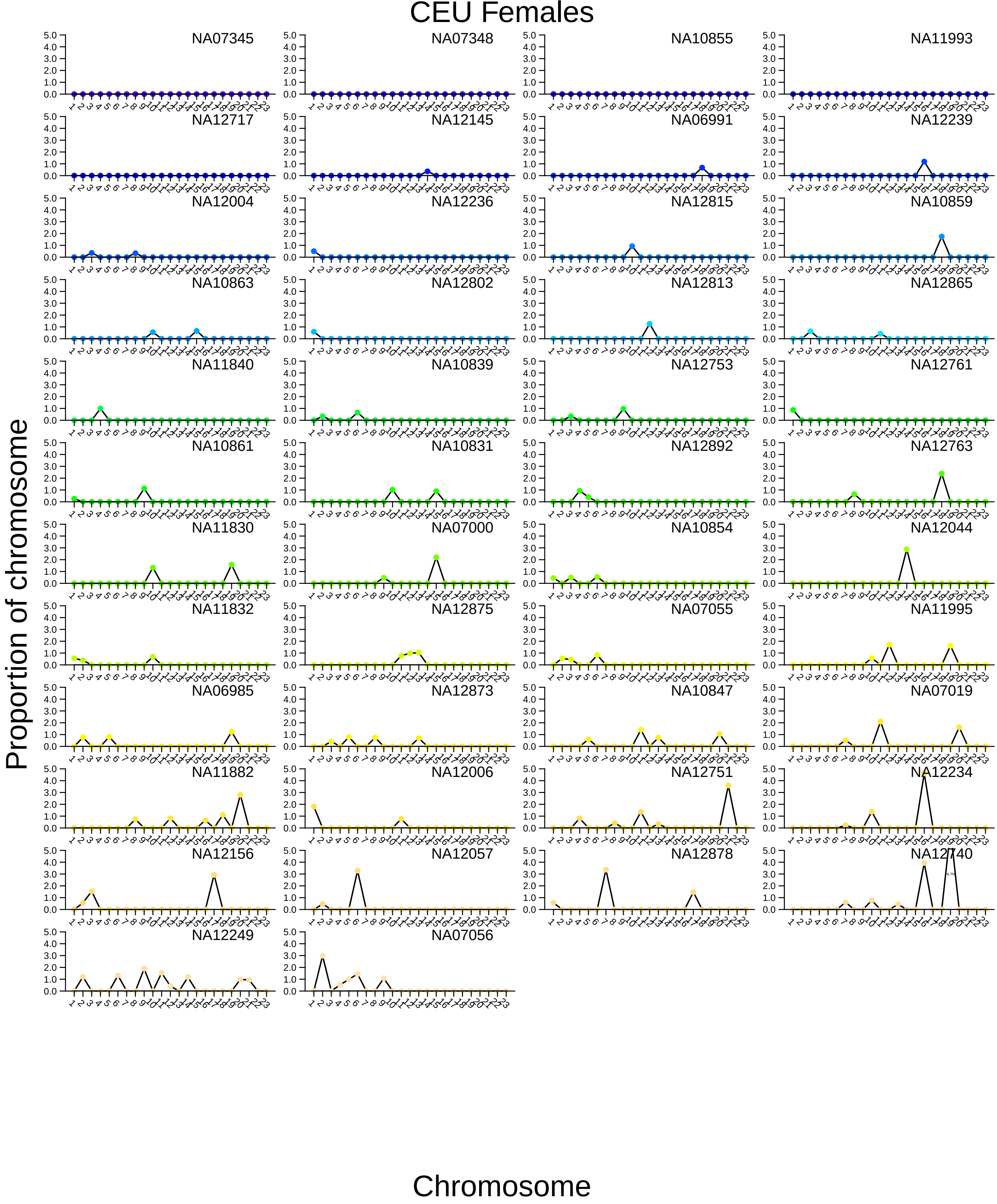
CEU Females
NA07345
NA07348
NA10855
NA11993
NA12717
NA12145
NA06991
NA12239
NA12004
NA12236
NA12815
NA10859
NA10863
NA12802
NA12813
NA12865
NA11840
NA10839
NA12753
NA12761
NA10861
NA10831
NA12892
NA12763
NA11830
NA07000
NA10854
NA12044
NA11832
NA12875
NA07055
NA11995
NA06985
NA12873
NA10847
NA07019
NA11882
NA12006
NA12751
NA12234
NA12156
NA12057
NA12878
NA12740
NA12249
NA07056
Proportion of chromosome
Chromosome

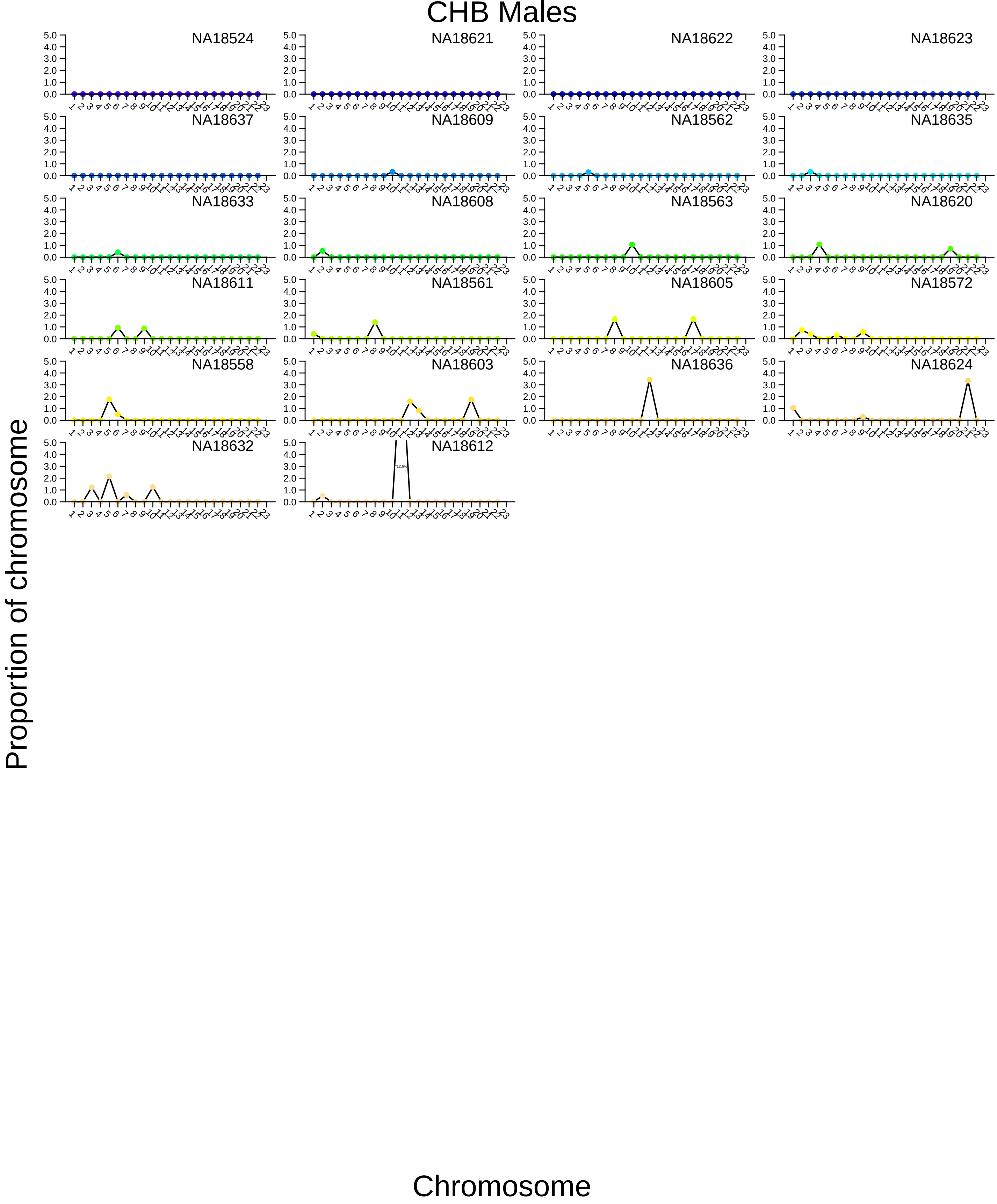
CHB Males
NA18524
NA18621
NA18622
NA18623
NA18637
NA18609
NA18562
NA18635
NA18633
NA18608
NA18563
NA18620
NA18611
NA18561
NA18605
NA18572
NA18558
NA18603
NA18636
NA18624
NA18632
NA18612
Proportion of chromosome
Chromosome

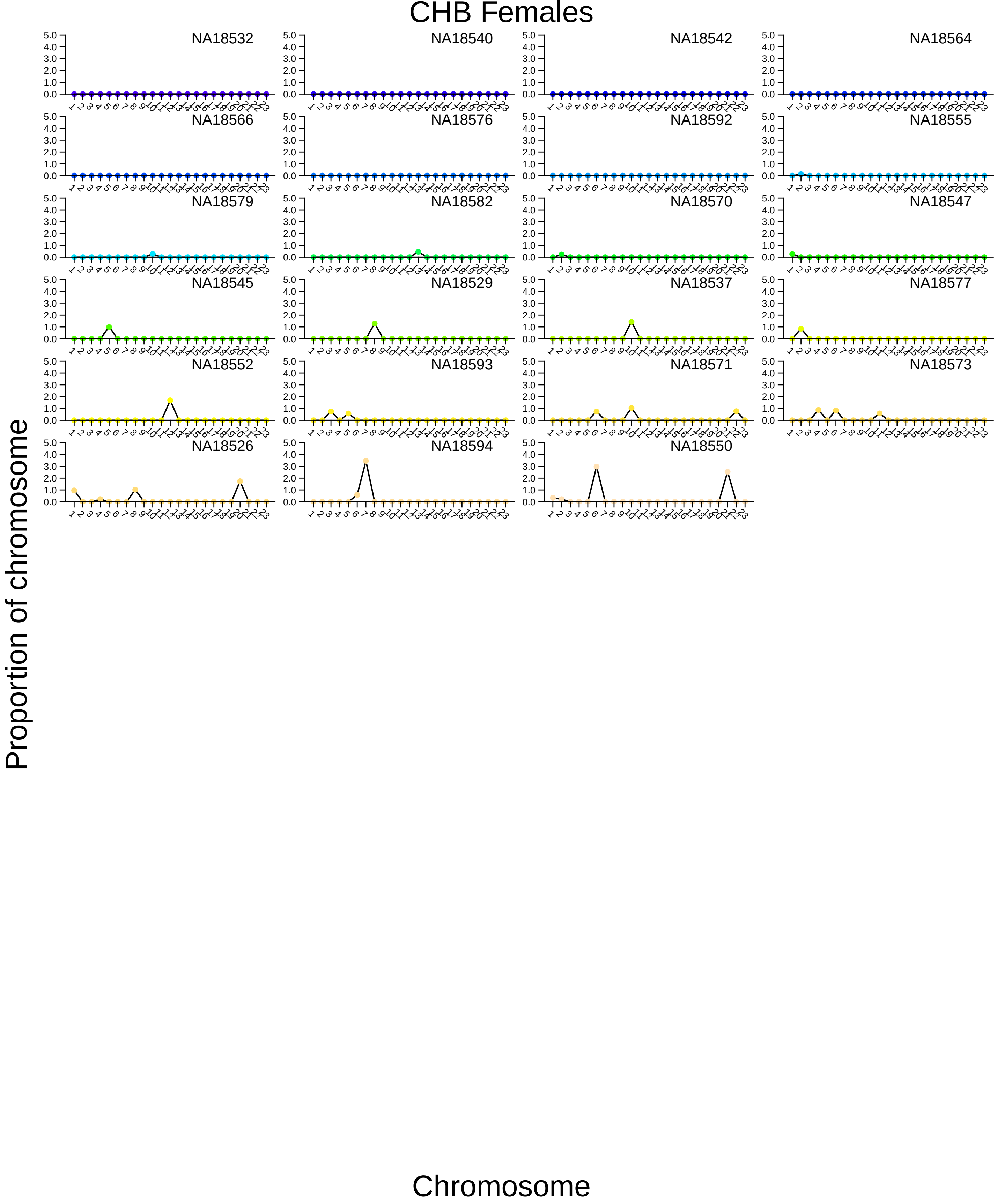
CHB Females
NA18532
NA18540
NA18542
NA18564
NA18566
NA18576
NA18592
NA18555
NA18579
NA18582
NA18570
NA18547
NA18545
NA18529
NA18537
NA18577
NA18552
NA18593
NA18571
NA18573
NA18526
NA18594
NA18550
Proportion of chromosome
Chromosome

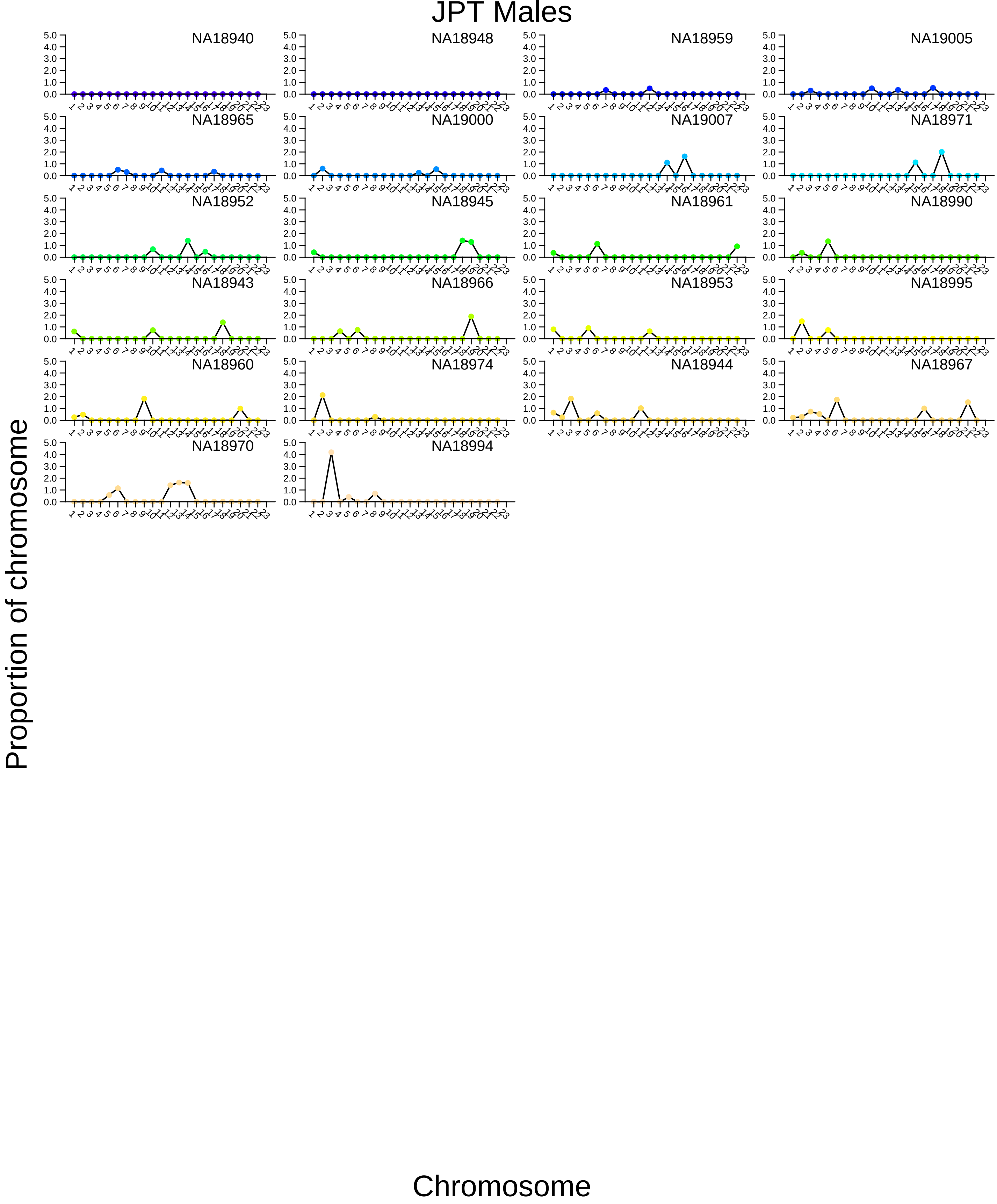
JPT Males
NA18940
NA18948
NA18959
NA19005
NA18965
NA19000
NA19007
NA18971
NA18952
NA18945
NA18961
NA18990
NA18943
NA18966
NA18953
NA18995
NA18960
NA18974
NA18944
NA18967
NA18970
NA18994
Proportion of chromosome
Chromosome

JPT Females

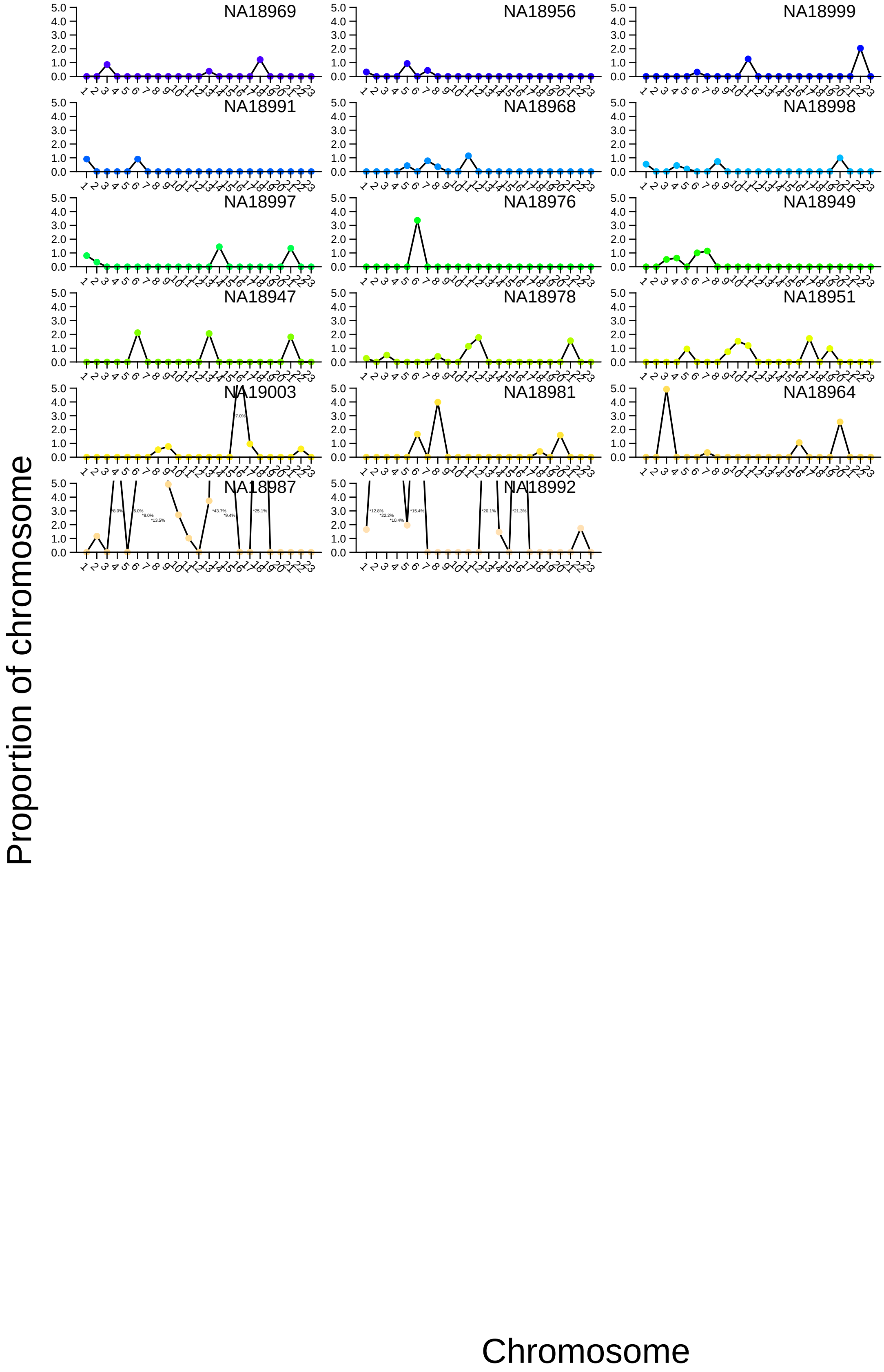
NA18969
NA18956
NA18999
NA18973
NA18991
NA18968
NA18998
NA18942
NA18997
NA18976
NA18949
NA18980
NA18947
NA18978
NA18951
NA18975
NA19003
NA18981
NA18964
NA18972
NA18987
NA18992
Proportion of chromosome
Chromosome
