# Supplementary material for: hzAnalyzer: detection, quantification, and visualization of contiguous homozygosity in high-density genotyping datasets
Source: Genome Biol. 2011 Mar 11;12(3):R21. doi: 10.1186/gb-2011-12-3-r21 (PMC3129671; doi:10.1186/gb-2011-12-3-r21)
Supplement: Additional file 11 — Figure S7. Phased haplotype plots for two regions with both high-ranking extAUC and high Fst/θ values between populations. Phased haplotypes were plotted for two example regions exhibiting high cross-population extAUC values as well as high population differentiation: page 1, Chr X:62.7-67Mb; page 2, Chr 14:65.4-67 Mb. [file gb-2011-12-3-r21-S11.PDF]

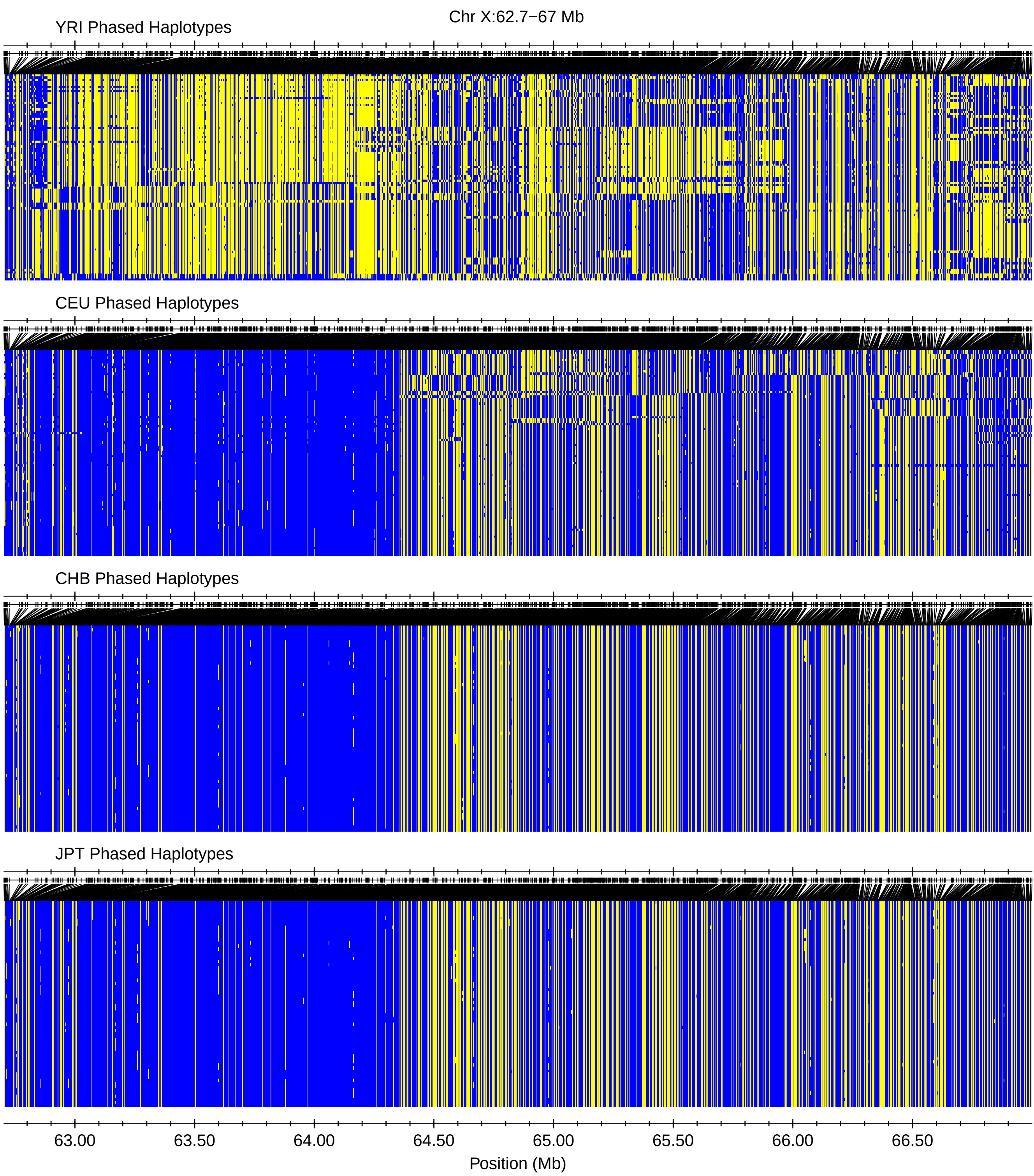
Chr X:62.7−67 Mb
YRI Phased Haplotypes
CEU Phased Haplotypes
CHB Phased Haplotypes
JPT Phased Haplotypes
63.00
63.50
64.00
64.50
65.00
65.50
66.00
66.50
Position (Mb)

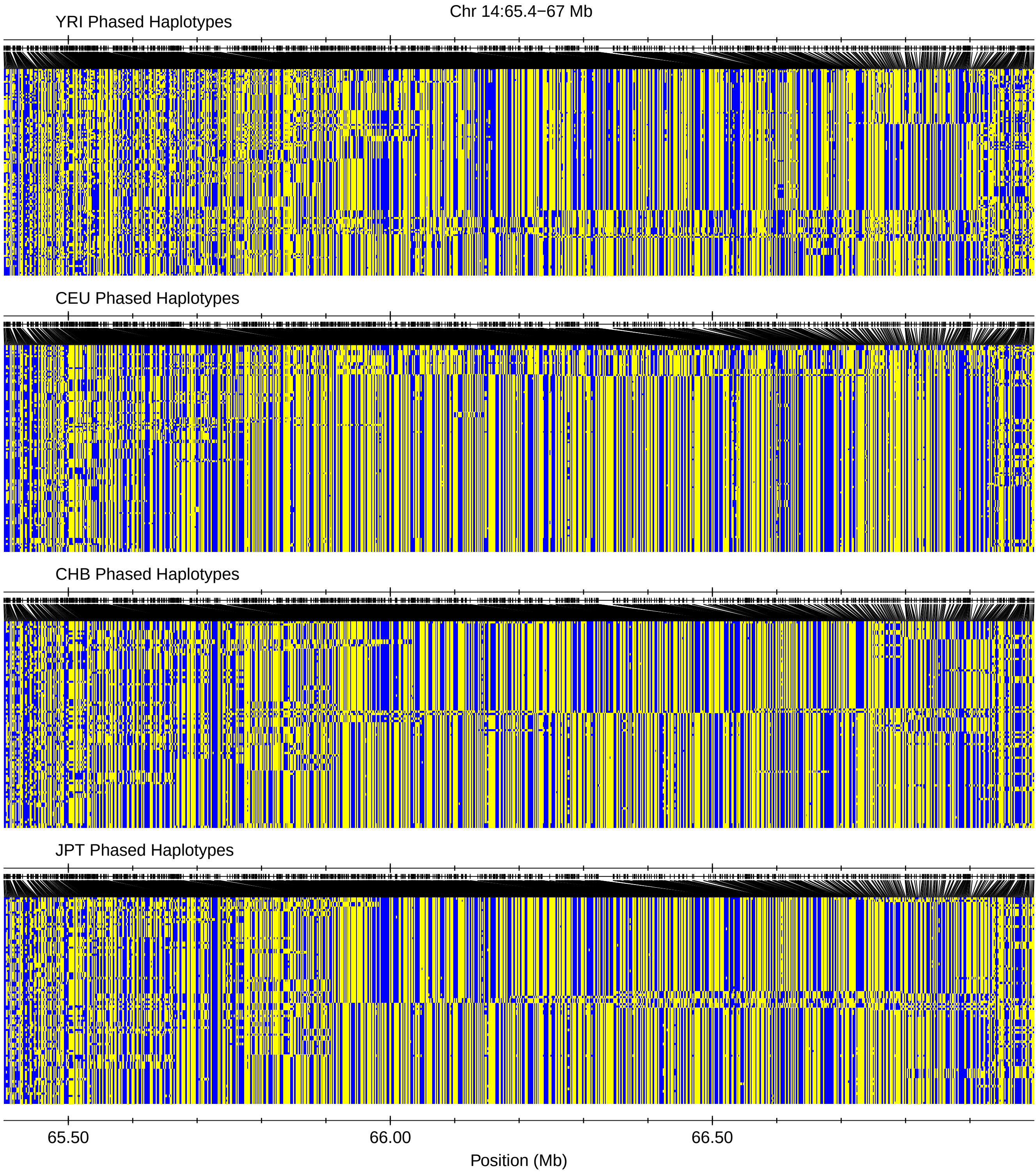

Chr 14:65.4−67 Mb
YRI Phased Haplotypes
CEU Phased Haplotypes
CHB Phased Haplotypes
JPT Phased Haplotypes
65.50
66.00
66.50
Position (Mb)
