# Supplementary material for: hzAnalyzer: detection, quantification, and visualization of contiguous homozygosity in high-density genotyping datasets
Source: Genome Biol. 2011 Mar 11;12(3):R21. doi: 10.1186/gb-2011-12-3-r21 (PMC3129671; doi:10.1186/gb-2011-12-3-r21)
Supplement: Additional file 12 — Figure S8. High-ranking extAUC values and high Fst/θ between East Asian population samples identify peaks intersecting multi-locus haplotypes with high frequency differences. Peaks were selected that had high-ranking extAUC values in the two groups (≥90th percentile) as well as extreme Fst/θ values (Chr X Fst/θ >0.0538, autosome Fst/θ >0.0360). The peaks were sorted in decreasing order using the proportion of loci with extreme Fst/θ values. The top five peaks for CHB and JPT are shown. [file gb-2011-12-3-r21-S12.PDF]

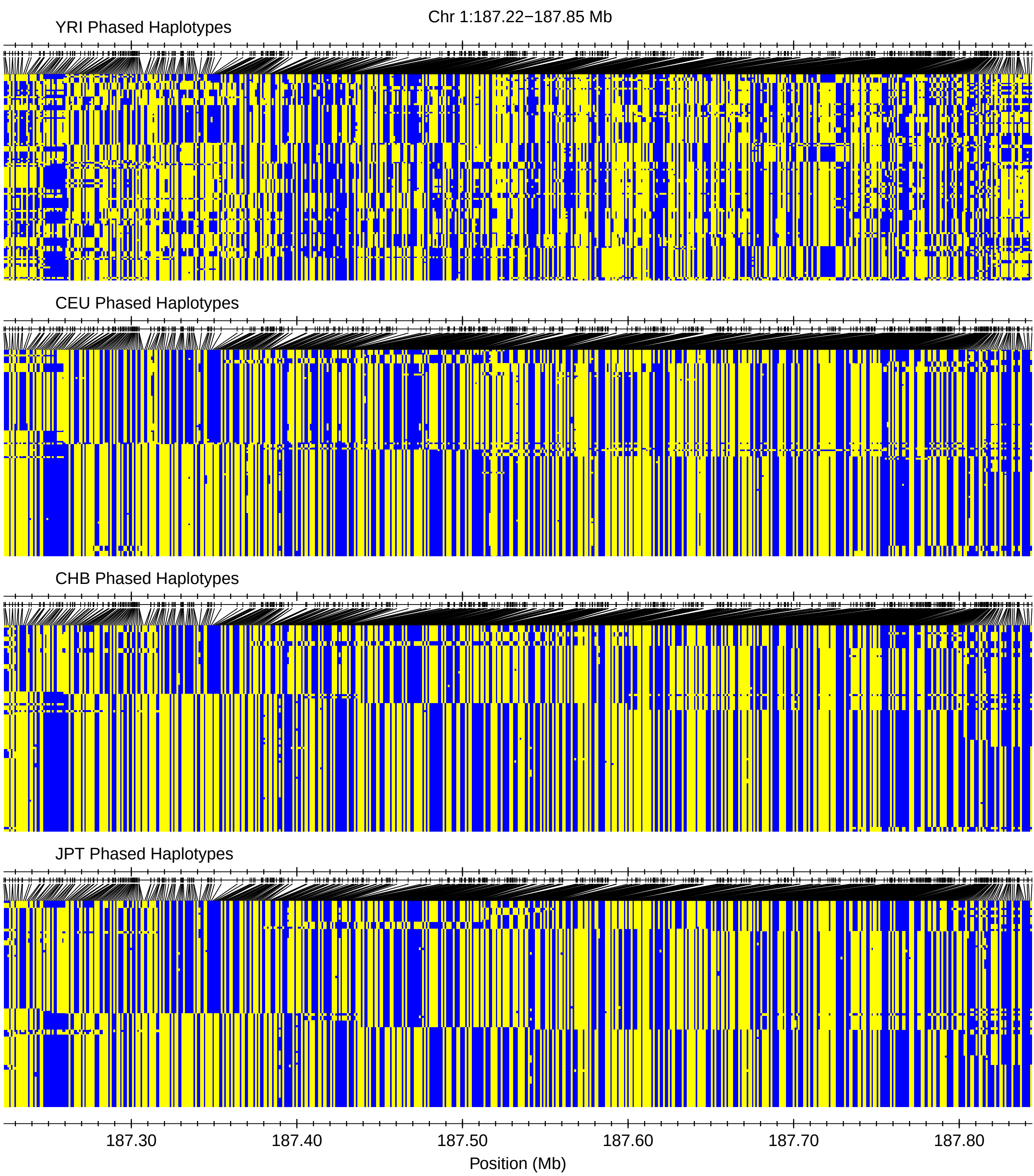

Chr 1:187.22−187.85 Mb
YRI Phased Haplotypes
CEU Phased Haplotypes
CHB Phased Haplotypes
JPT Phased Haplotypes
187.30
187.40
187.50
187.60
187.70
187.80
Position (Mb)

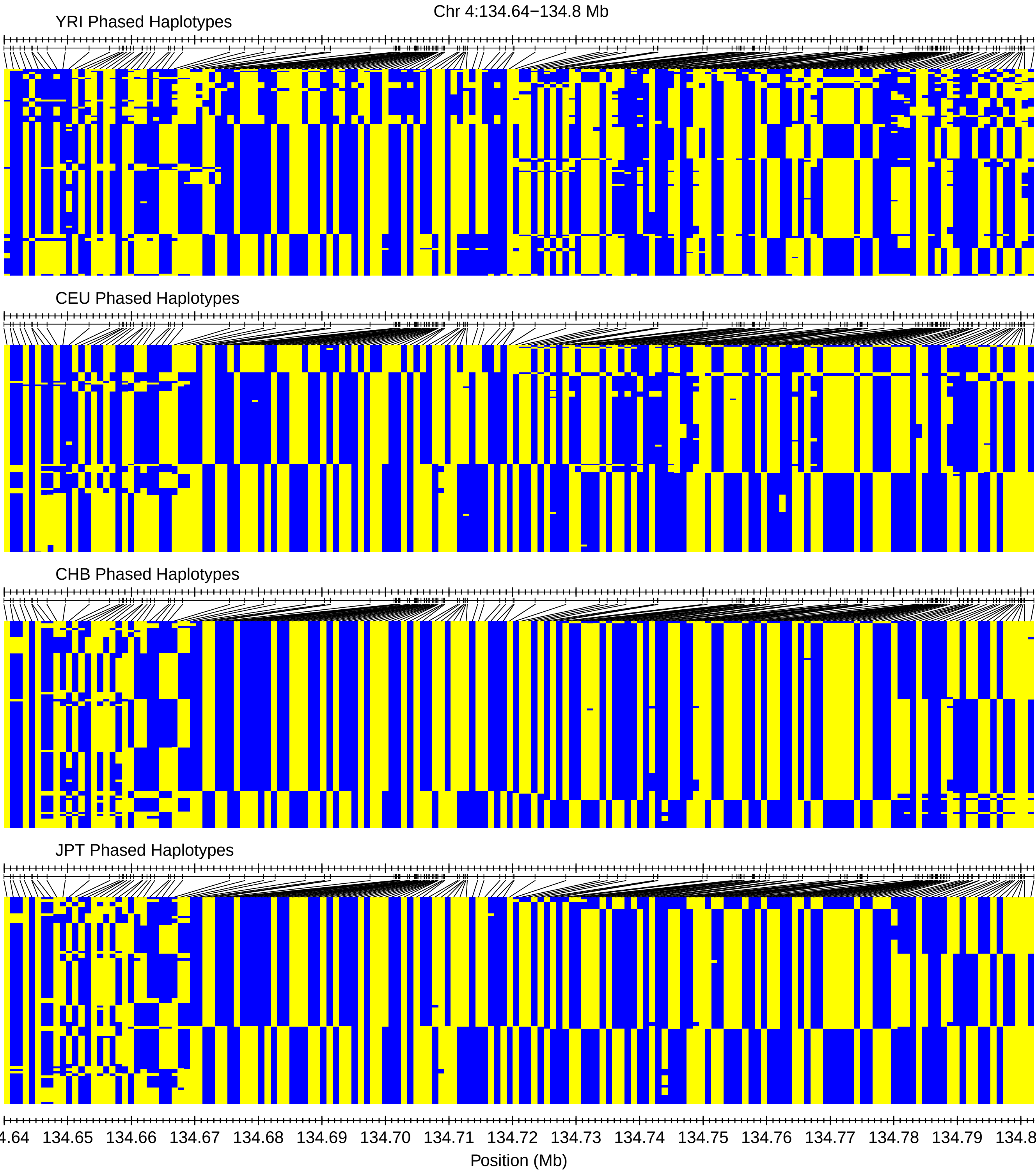
Chr 4:134.64−134.8 Mb
YRI Phased Haplotypes
CEU Phased Haplotypes
CHB Phased Haplotypes
JPT Phased Haplotypes
134.64
134.65
134.66
134.67
134.68
134.69
134.70
134.71
134.72
134.73
134.74
134.75
134.76
134.77
134.78
134.79
134.8
Position (Mb)

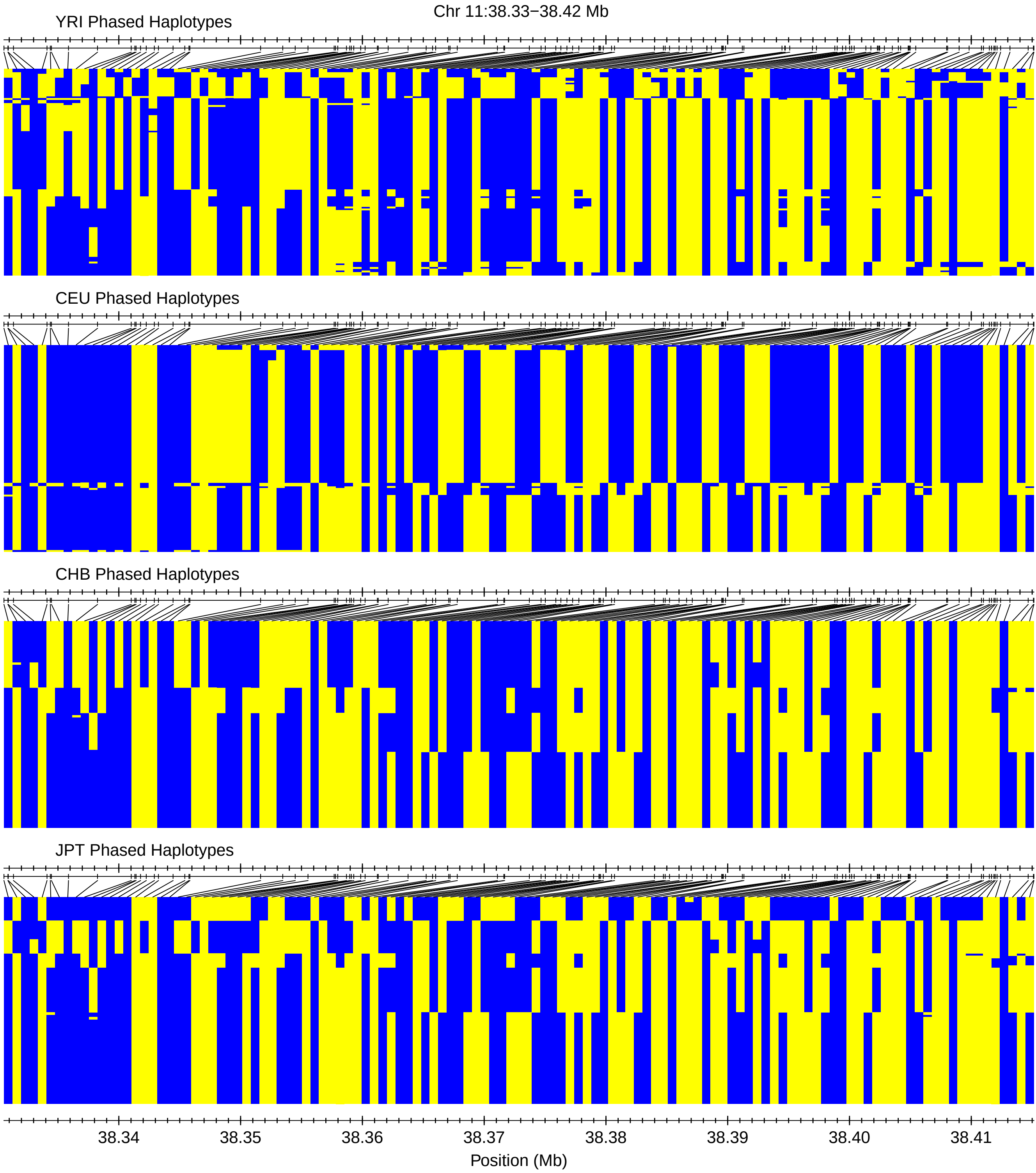
Chr 11:38.33−38.42 Mb
YRI Phased Haplotypes
CEU Phased Haplotypes
CHB Phased Haplotypes
JPT Phased Haplotypes
38.34
38.35
38.36
38.37
38.38
38.39
38.40
38.41
Position (Mb)

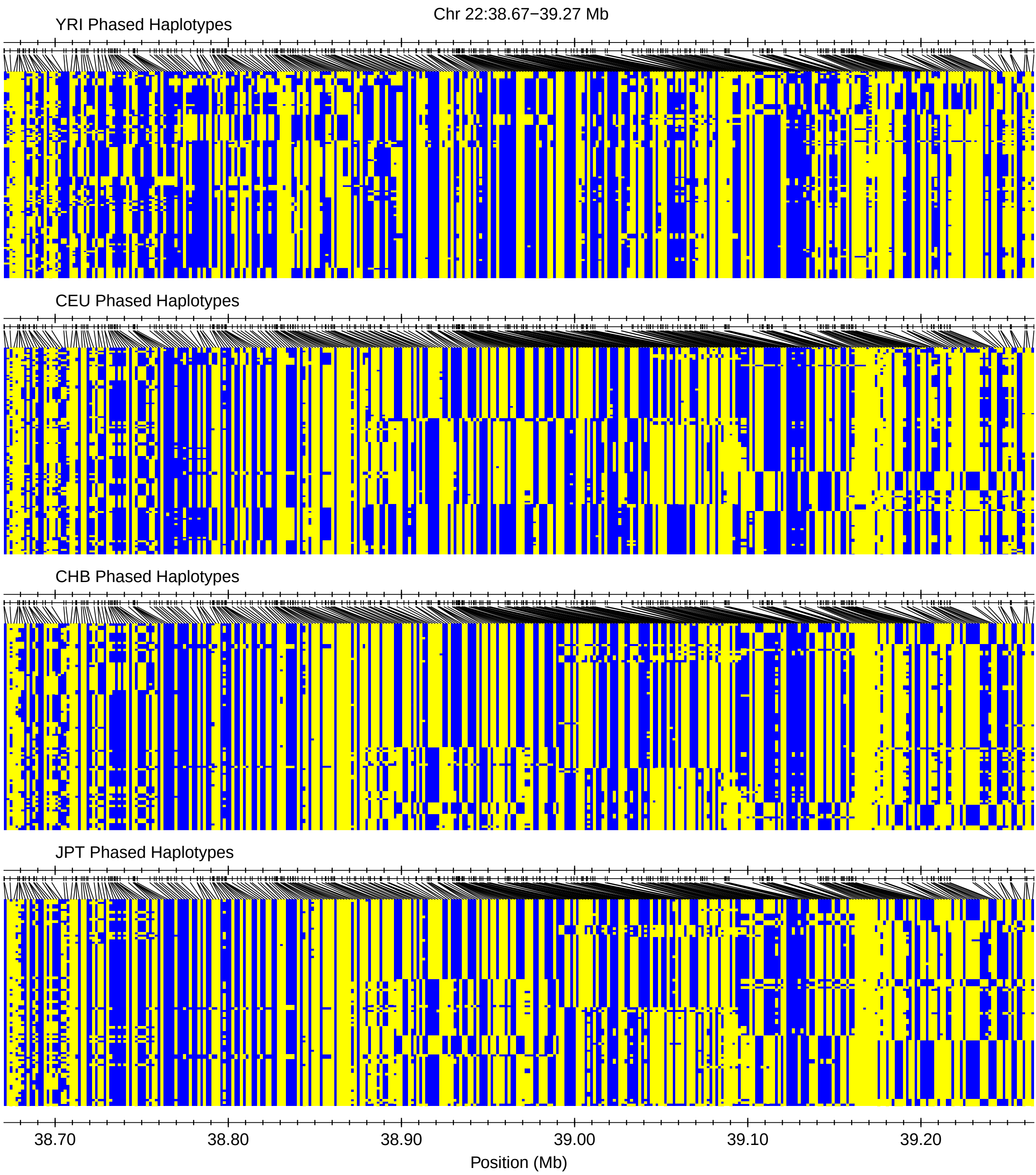

Chr 22:38.67−39.27 Mb
YRI Phased Haplotypes
CEU Phased Haplotypes
CHB Phased Haplotypes
JPT Phased Haplotypes
38.70
38.80
38.90
39.00
39.10
39.20
Position (Mb)

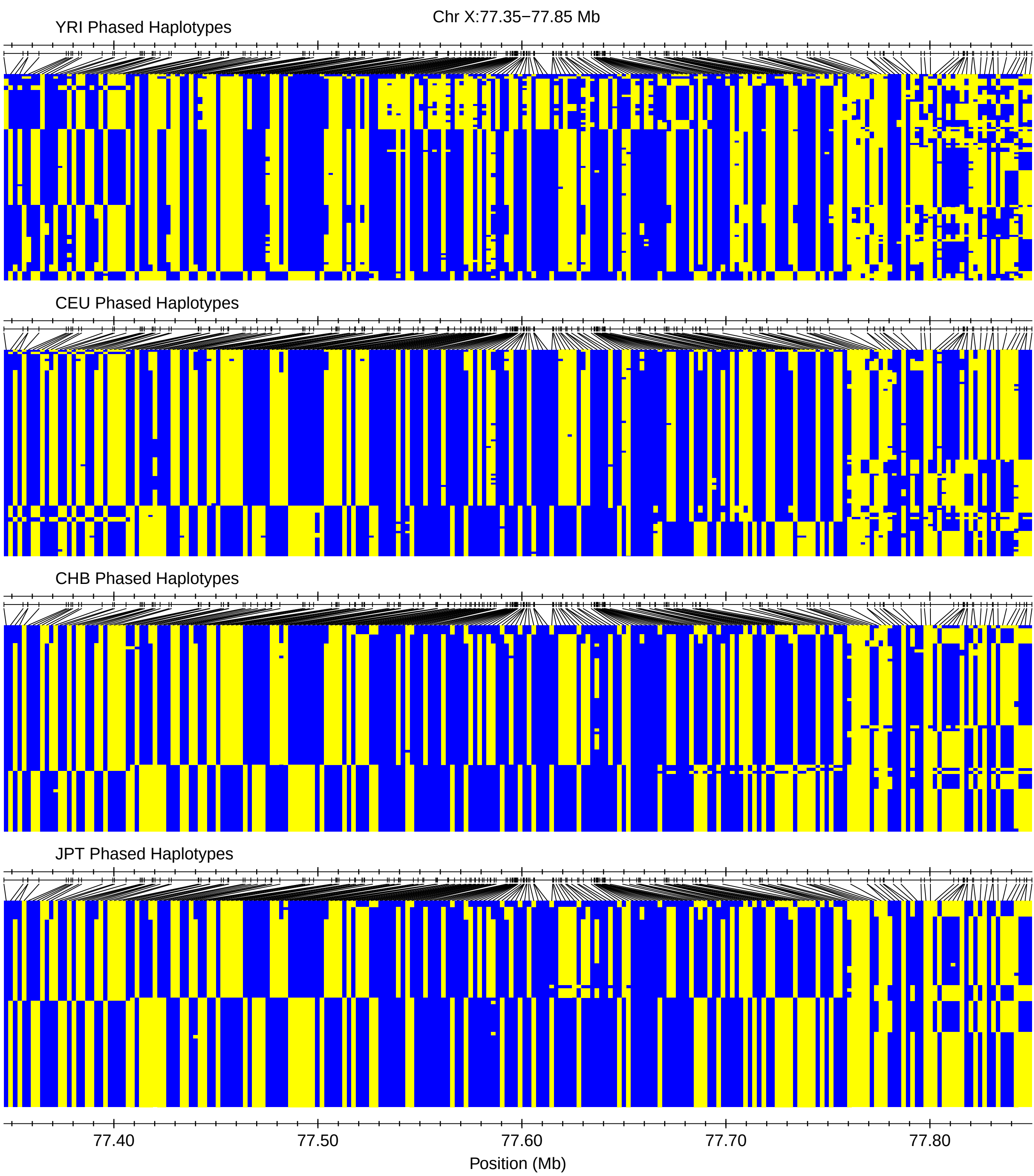
Chr X:77.35−77.85 Mb
YRI Phased Haplotypes
CEU Phased Haplotypes
CHB Phased Haplotypes
JPT Phased Haplotypes
77.40
77.50
77.60
77.70
77.80
Position (Mb)

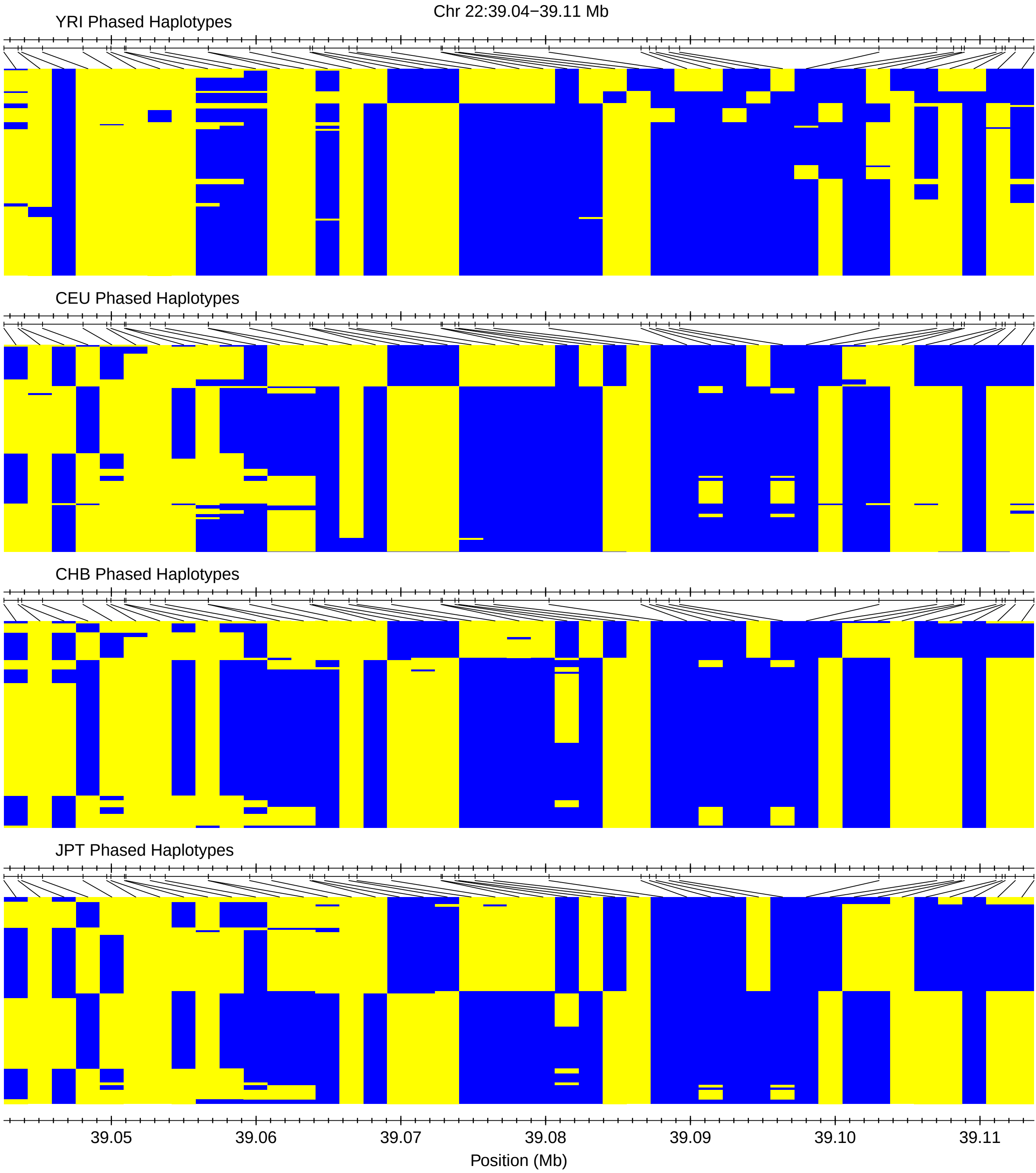

Chr 22:39.04−39.11 Mb
YRI Phased Haplotypes
CEU Phased Haplotypes
CHB Phased Haplotypes
JPT Phased Haplotypes
39.05
39.06
39.07
39.08
39.09
39.10
39.11
Position (Mb)

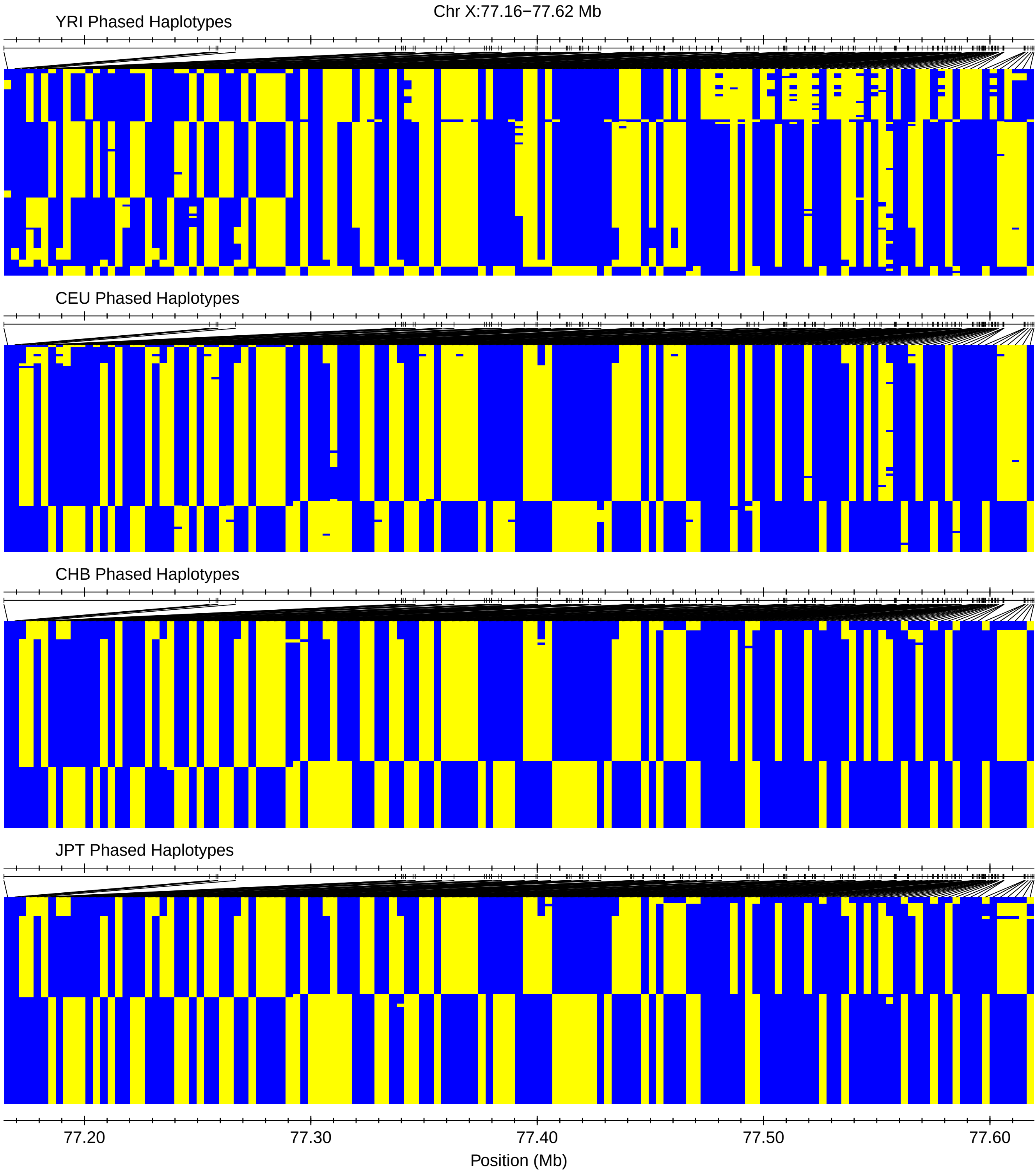
Chr X:77.16−77.62 Mb
YRI Phased Haplotypes
CEU Phased Haplotypes
CHB Phased Haplotypes
JPT Phased Haplotypes
77.20
77.30
77.40
77.50
77.60
Position (Mb)

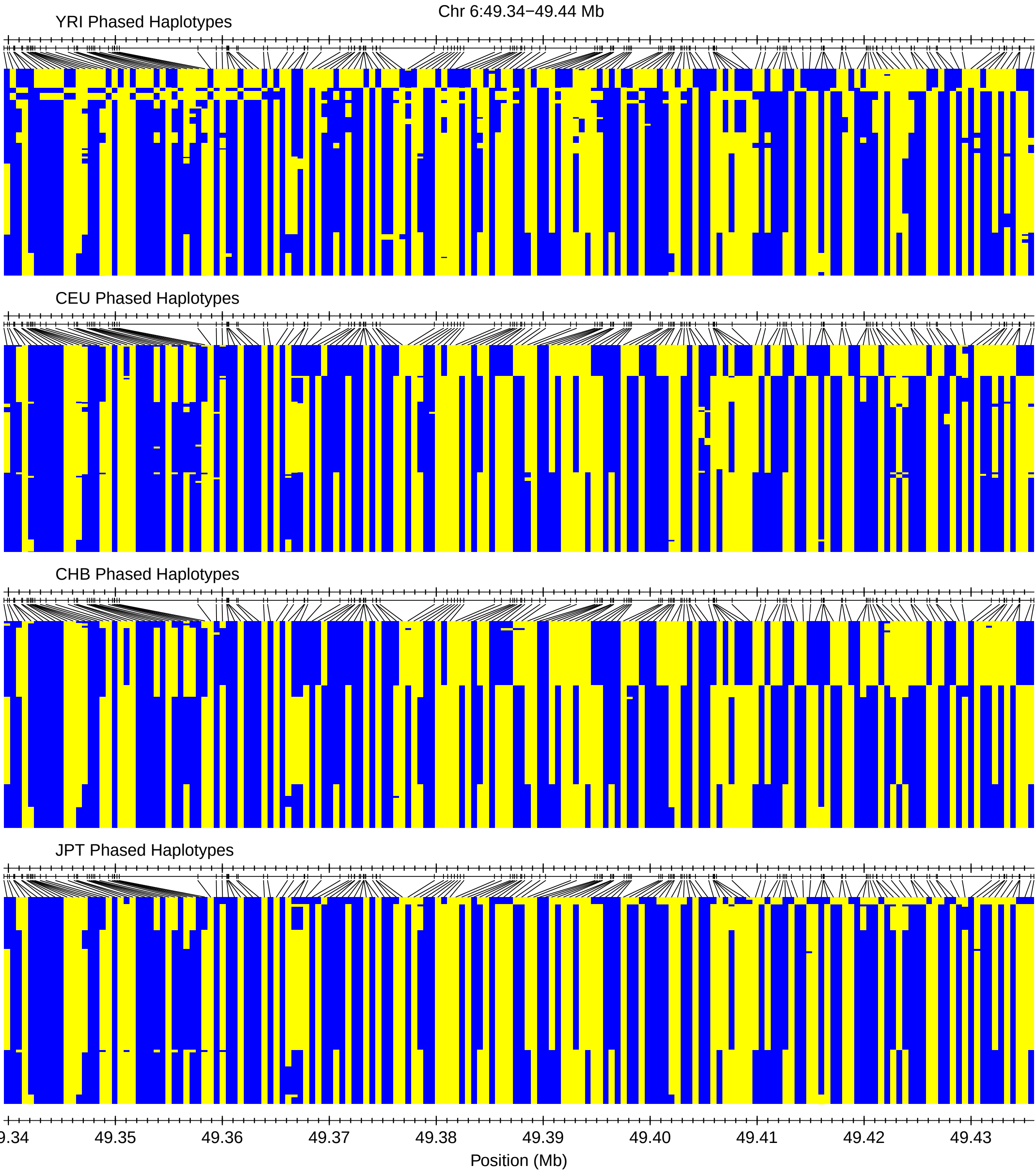

Chr 6:49.34−49.44 Mb
YRI Phased Haplotypes
CEU Phased Haplotypes
CHB Phased Haplotypes
JPT Phased Haplotypes
49.34
49.35
49.36
49.37
49.38
49.39
49.40
49.41
49.42
49.43
Position (Mb)

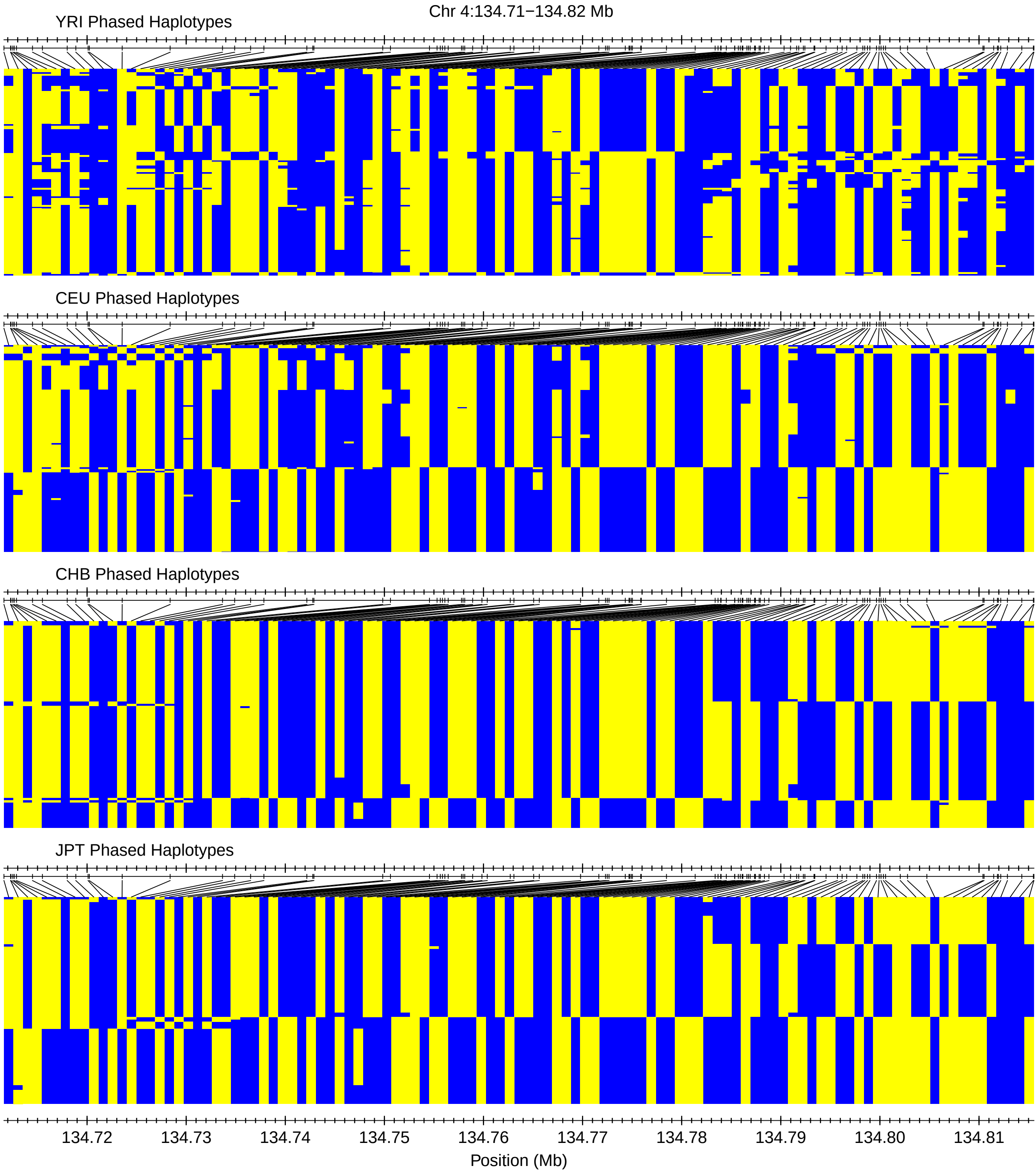
Chr 4:134.71−134.82 Mb
YRI Phased Haplotypes
CEU Phased Haplotypes
CHB Phased Haplotypes
JPT Phased Haplotypes
134.72
134.73
134.74
134.75
134.76
134.77
134.78
134.79
134.80
134.81
Position (Mb)

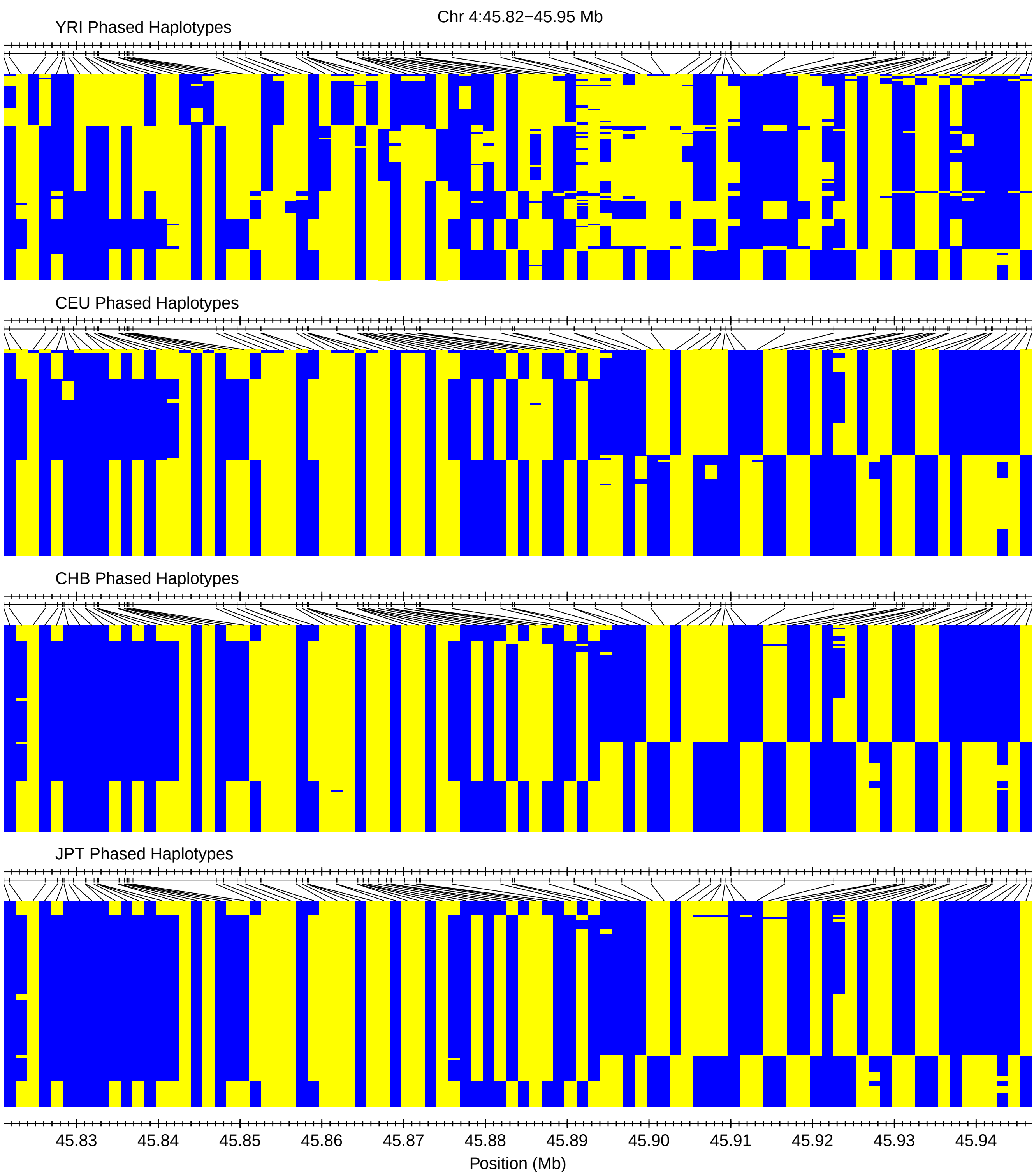
Chr 4:45.82−45.95 Mb
YRI Phased Haplotypes
CEU Phased Haplotypes
CHB Phased Haplotypes
JPT Phased Haplotypes
45.83
45.84
45.85
45.86
45.87
45.88
45.89
45.90
45.91
45.92
45.93
45.94
Position (Mb)
