# Supplementary material for: hzAnalyzer: detection, quantification, and visualization of contiguous homozygosity in high-density genotyping datasets
Source: Genome Biol. 2011 Mar 11;12(3):R21. doi: 10.1186/gb-2011-12-3-r21 (PMC3129671; doi:10.1186/gb-2011-12-3-r21)
Supplement: Additional file 13 — Figure S9. Phased haplotype plots in combined fixation candidate regions. Phased haplotypes were plotted for combined fixation candidate regions that are mentioned in the Discussion. These include three regions on chromosome X that were not reported in the other examined datasets: page 1, Chr X:104.2-105.5 Mb; page 2, Chr X:113.7-114.4 Mb; page 3, Chr X:126.1-127.7 Mb, and one region in JPT intersecting the EXOC6B gene; page 4, Chr 2:71.9-73.1 Mb. [file gb-2011-12-3-r21-S13.PDF]

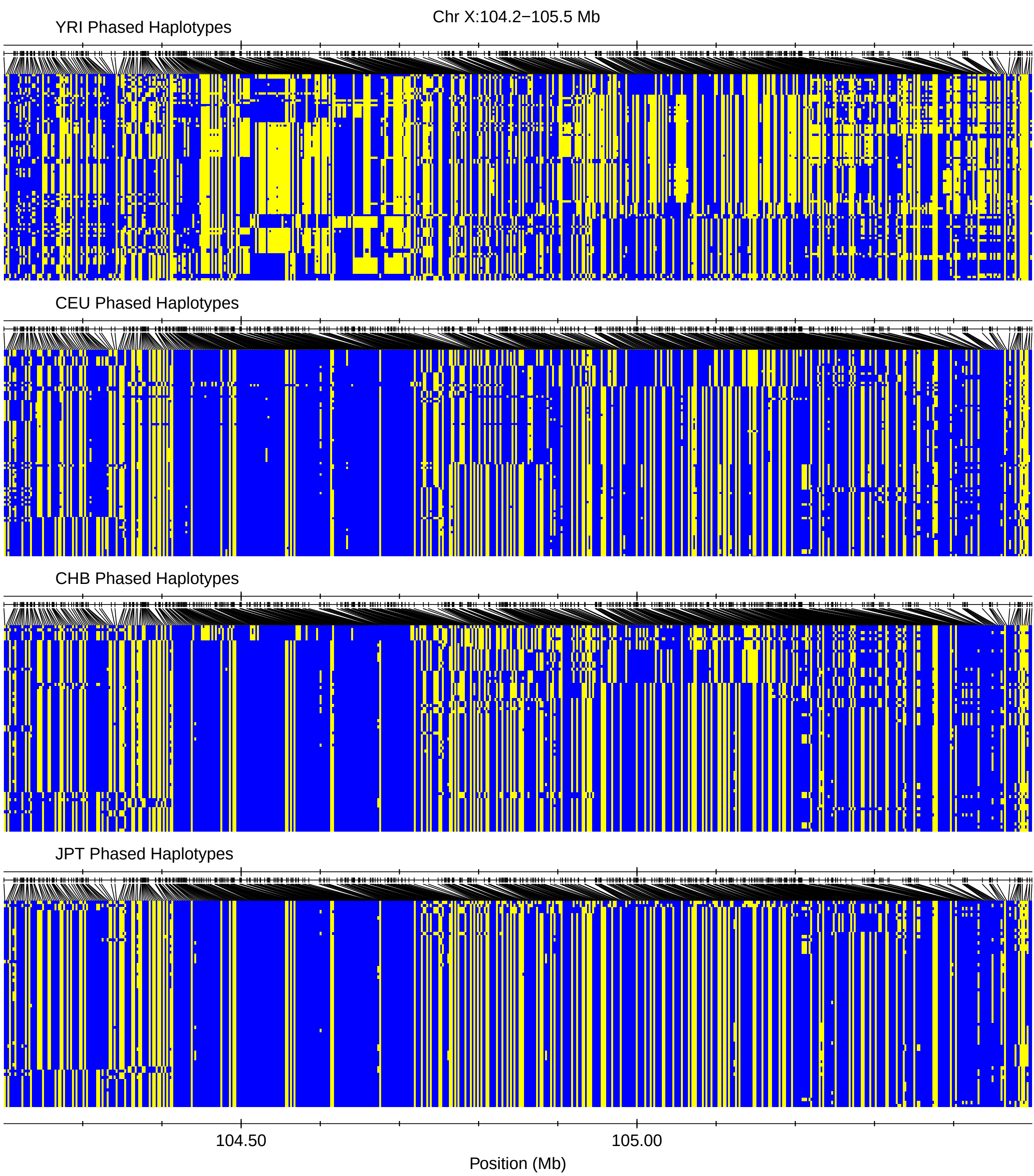

Chr X:104.2−105.5 Mb
YRI Phased Haplotypes
CEU Phased Haplotypes
CHB Phased Haplotypes
JPT Phased Haplotypes
104.50
105.00
Position (Mb)

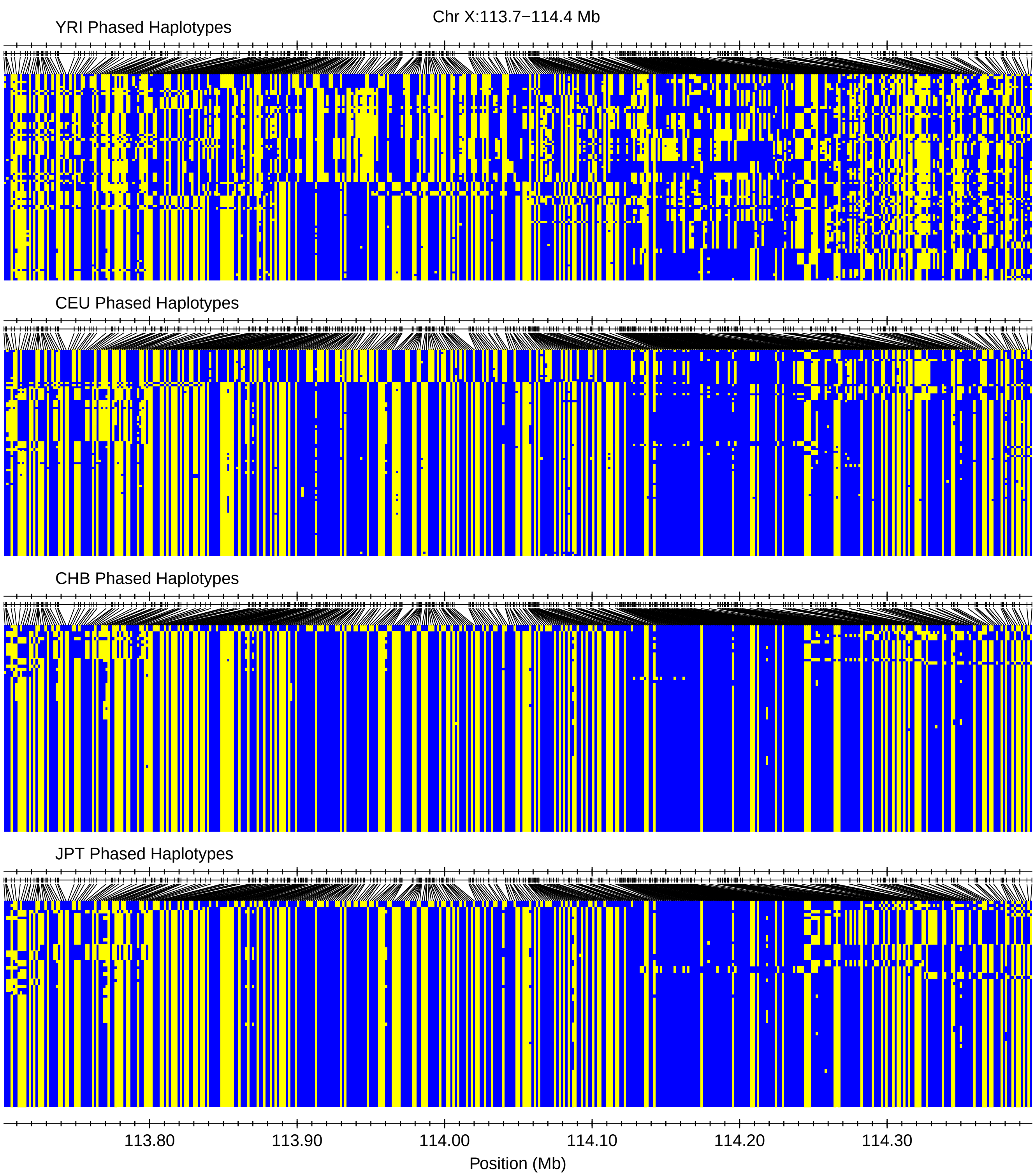

Chr X:113.7−114.4 Mb
YRI Phased Haplotypes
CEU Phased Haplotypes
CHB Phased Haplotypes
JPT Phased Haplotypes
113.80
113.90
114.00
114.10
114.20
114.30
Position (Mb)

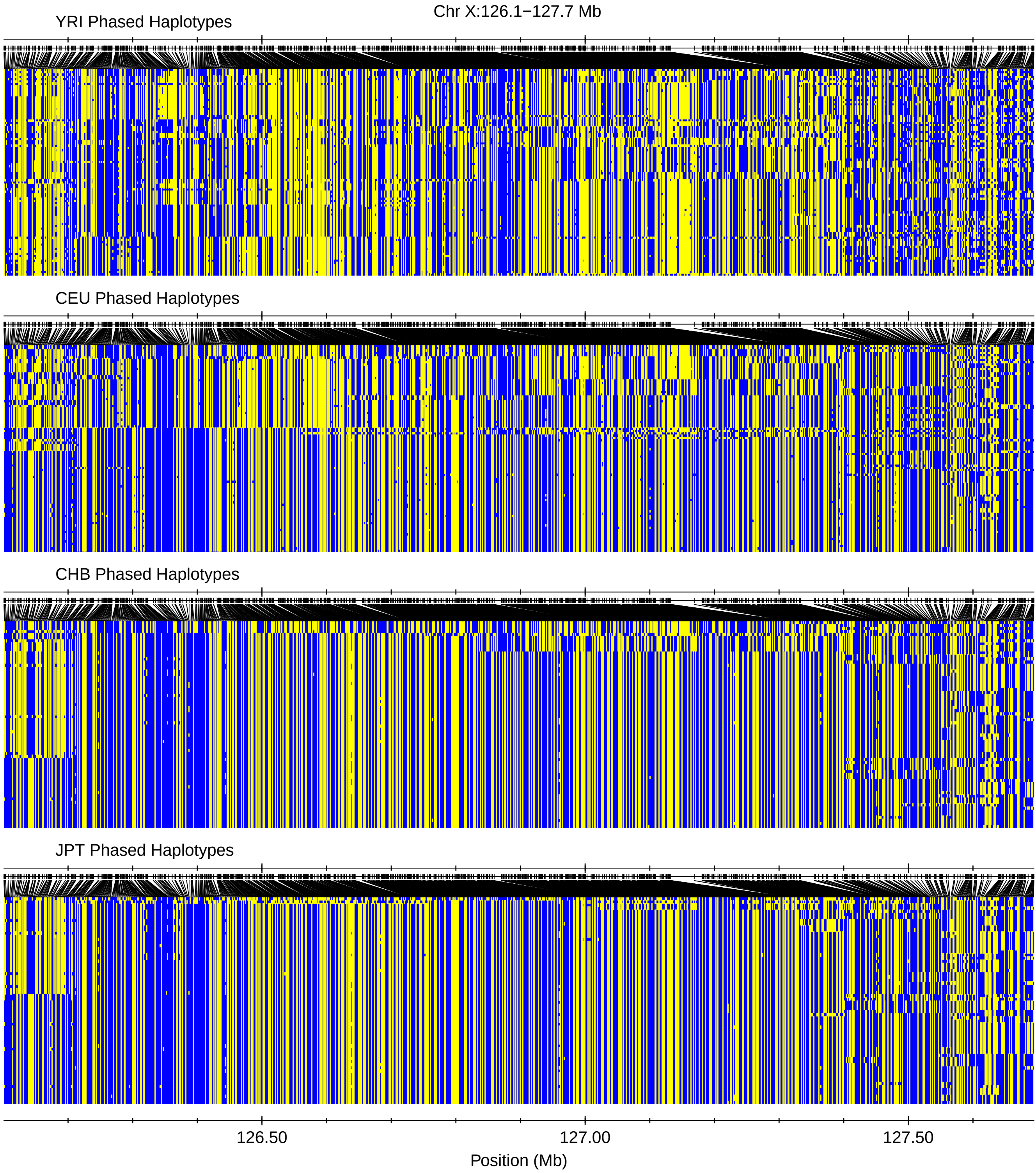

Chr X:126.1−127.7 Mb
YRI Phased Haplotypes
CEU Phased Haplotypes
CHB Phased Haplotypes
JPT Phased Haplotypes
126.50
127.00
127.50
Position (Mb)

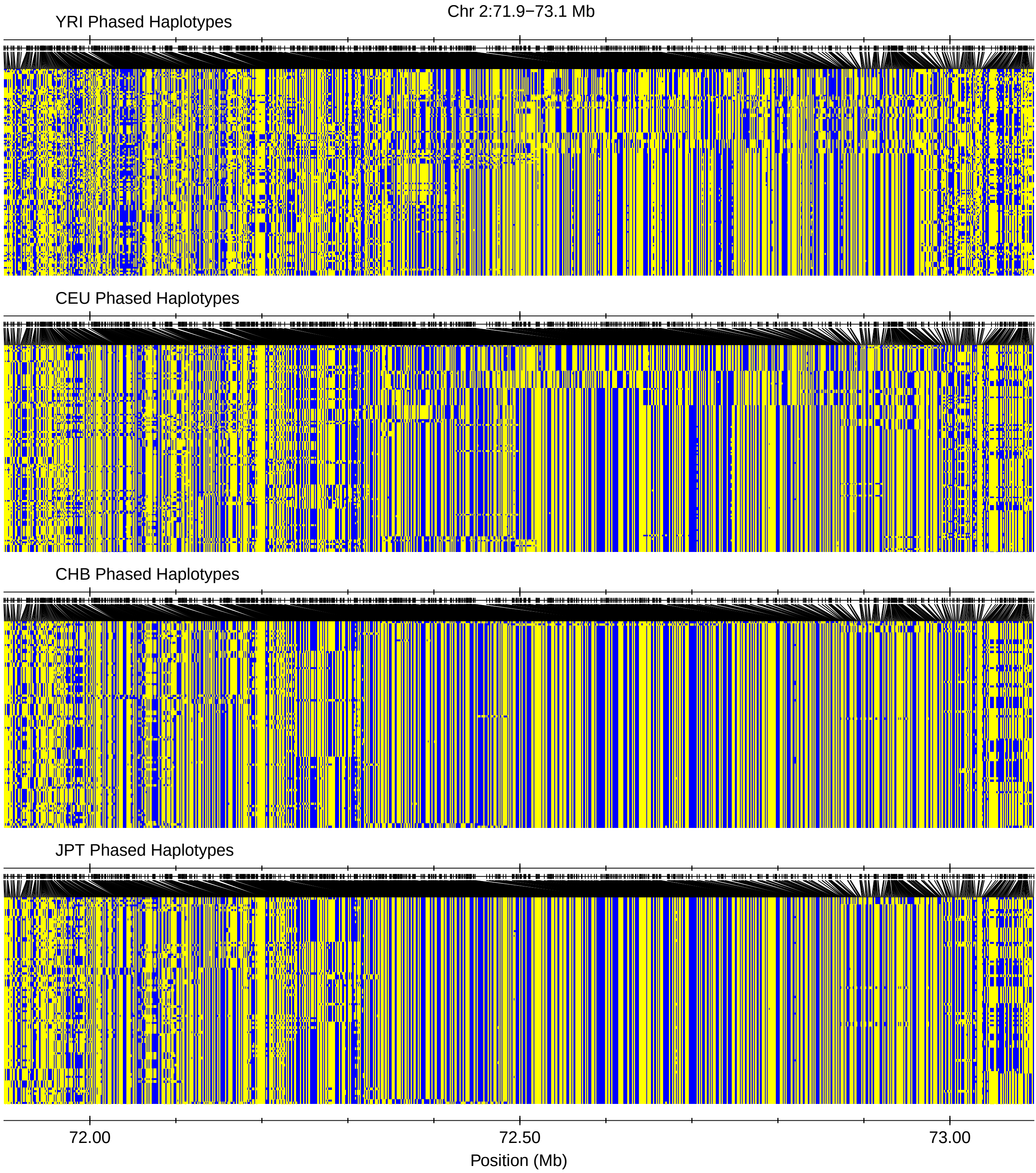
Chr 2:71.9−73.1 Mb
YRI Phased Haplotypes
CEU Phased Haplotypes
CHB Phased Haplotypes
JPT Phased Haplotypes
72.00
72.50
73.00
Position (Mb)
